# SRSF protein kinase 1 modulates RAN translation and suppresses CGG repeat toxicity

**DOI:** 10.15252/emmm.202114163

**Published:** 2021-09-20

**Authors:** Indranil Malik, Yi‐Ju Tseng, Shannon E Wright, Kristina Zheng, Prithika Ramaiyer, Katelyn M Green, Peter K Todd

**Affiliations:** ^1^ Department of Neurology University of Michigan Ann Arbor MI USA; ^2^ Cellular and Molecular Biology Graduate Program University of Michigan Ann Arbor MI USA; ^3^ Neuroscience Graduate Program University of Michigan Ann Arbor MI USA; ^4^ Ann Arbor Veterans Administration Healthcare Ann Arbor MI USA

**Keywords:** C9orf72 ALS, CGG repeats, FXTAS, RAN translation, SRSF, Genetics, Gene Therapy & Genetic Disease, Neuroscience, Translation & Protein Quality

## Abstract

Transcribed CGG repeat expansions cause neurodegeneration in Fragile X‐associated tremor/ataxia syndrome (FXTAS). CGG repeat RNAs sequester RNA‐binding proteins (RBPs) into nuclear foci and undergo repeat‐associated non‐AUG (RAN) translation into toxic peptides. To identify proteins involved in these processes, we employed a CGG repeat RNA‐tagging system to capture repeat‐associated RBPs by mass spectrometry in mammalian cells. We identified several SR (serine/arginine‐rich) proteins that interact selectively with CGG repeats basally and under cellular stress. These proteins modify toxicity in a *Drosophila* model of FXTAS. Pharmacologic inhibition of serine/arginine protein kinases (SRPKs), which alter SRSF protein phosphorylation, localization, and activity, directly inhibits RAN translation of CGG and GGGGCC repeats (associated with C9orf72 ALS/FTD) and triggers repeat RNA retention in the nucleus. Lowering SRPK expression suppressed toxicity in both FXTAS and C9orf72 ALS/FTD model flies, and SRPK inhibitors suppressed CGG repeat toxicity in rodent neurons. Together, these findings demonstrate roles for CGG repeat RNA binding proteins in RAN translation and repeat toxicity and support further evaluation of SRPK inhibitors in modulating RAN translation associated with repeat expansion disorders.

## Graphical abstract

Fragile X‐associated tremor/ataxia syndrome (FXTAS) is an adult‐onset neurodegenerative disease caused by a CGG trinucleotide repeat expansion in FMR1. This study identified CGG repeat RNA binding proteins and their kinases that regulate repeat associated non‐AUG (RAN) translation and neurotoxicity.

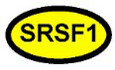

**The paper explained**

**Problem**

Over 50 different nucleotide repeat expansions cause neurodevelopmental and neurodegenerative disorders in humans. These repeats as RNA interact with specific RNA binding proteins to elicit toxicity through protein sequestration as well as repeat‐associated non‐AUG‐initiated translation. However, accurate catalogues of which RNA binding proteins bind these repeats in different cellular compartments and how they influence their biology is unclear.

**Results**

Using an innovative in‐cell CGG repeat capture technique, this study identified SR proteins and SR protein kinases (SRPKs) as key regulators of repeat RNA behavior, RAN translation, and repeat toxicity across model systems. Pharmacological targeting of SRPK1 alleviated toxicity and enhanced survival in CGG repeat expressing neurons by both suppressing repeat RNA translocation to the cytoplasm and directly inhibiting RAN translation.

**Impact**

This study establishes SRPK1 as a potential modifier of repeat toxicity and RAN translation across multiple repeats. These results provide a foundation for the development of new therapeutic approaches for additional repeat expansion disorders.

## Introduction

Fragile X‐associated Tremor/Ataxia Syndrome (FXTAS) is an age‐related neurodegenerative disorder, which impacts approximately 1/5,000 people (Jacquemont *et al*, 2004; Tassone *et al*, 2012). It results from a transcribed CGG repeat expansion in the 5’ UTR of the fragile X gene, *FMR1* (Hagerman *et al*, 2001). Normally healthy humans harbor approximately 30 repeats, which get expanded to 55–200 repeats in case of FXTAS (Hagerman & Hagerman, 2015). Patients develop imbalance, dementia, parkinsonism, and tremors starting in their 50's or 60's (Jacquemont *et al*, 2003). Pathologically, the condition is associated with diffuse neuronal loss and brain atrophy as well as accumulation of ubiquitinated inclusions within neurons and glia throughout the brain (Greco *et al*, 2002, 2006; Ariza *et al*, 2016). FXTAS is an inexorably progressive and fatal condition without effective treatment. Thus, identifying targetable factor(s) involved in CGG repeat expansion‐associated toxicity may help in the development of new therapeutics.

CGG repeats are thought to elicit toxicity through two non‐exclusive mechanisms (Glineburg *et al*, 2018). Expanded repeat RNAs can elicit gain‐of‐function (GOF) toxicity by sequestering essential RNA‐binding proteins (RBPs) and forming RNA–RNA and RNA–protein complex condensates (Jazurek *et al*, 2016; Jain & Vale, 2017; Glineburg *et al*, 2018). This pathologic process is best exemplified by the sequestration of muscleblind (MBNL) proteins by CUG repeat RNA in myotonic dystrophy type 1 (DM1) (Taneja *et al*, 1995; Miller *et al*, 2000). The repeat mRNA and MBNL protein avidly colocalize into RNA foci in both patient tissues and in model systems (Miller *et al*, 2000). Moreover, DM1 patients have splicing defects and clinical phenotypes that mimic those seen with genetic ablation of MBNL1 and upregulation of MBNL1 suppresses relevant disease phenotypes in DM1 disease models (Mankodi *et al*, 2000; Kanadia *et al*, 2003, 2006; Wang *et al*, 2019). At CGG repeat expansions that cause FXTAS, *in vitro* RNA pull‐down assays identified Pur alpha, hnRNP A2/B1, Sam68, and DROSHA/DGCR8 as potential repeat RNA targets (Jin *et al*, 2007; Sofola *et al*, 2007; Sellier *et al*, 2010, 2013). Overexpression of some of these factors in *Drosophila* can suppress CGG repeat elicited phenotypes (Jin *et al*, 2007; Sofola *et al*, 2007). However, direct manipulation of these factors has not yet recapitulated (in their absence) or suppressed (in their overexpression) disease relevant phenotypes in rodent or human neuronal model systems.

Repeat RNAs also support translational initiation in the absence of AUG start codons through repeat‐associated non‐AUG (RAN) translation (Zu *et al*, 2011). RAN translation is supported by many repeat expansions including CGG repeats associated with FXTAS and GGGGCC repeats associated with ALS/FTD (Ash *et al*, 2013; Mori *et al*, 2013; Todd *et al*, 2013; Bañez‐Coronel *et al*, 2015; Ishiguro *et al*, 2017; Zu *et al*, 2017; Soragni *et al*, 2018). RAN translation occurs across multiple reading frames of the same repeat to produce toxic repetitive polypeptides that accumulate in patient neurons and tissues (Gendron *et al*, 2013; Zu *et al*, 2013, 2017; Krans *et al*, 2016; Soragni *et al*, 2018). Existing experimental evidence for multiple repeat expansions suggests these RAN‐translated peptides may play an active role in disease pathogenesis. For example, expression of dipeptide repeat products resulting from C9 ALS/FTD GGGGCC RAN translation are sufficient to elicit toxicity in model systems even in the absence of repetitive RNA (May *et al*, 2014; Mizielinska *et al*, 2014; Wen *et al*, 2014; Jovičić *et al*, 2015; Lee *et al*, 2016; Zhang *et al*, 2018, 2019).

In FXTAS, RAN translation in the GGC reading frame generates a polyglycine‐containing peptide termed FMRpolyG, which accumulates into ubiquitinated inclusions in both model systems and patient tissues (Todd *et al*, 2013). Induced expression of repeats that support FMRpolyG synthesis elicit toxicity in heterologous cells, rodent neurons, flies, and transgenic mice (Todd *et al*, 2013; Buijsen *et al*, 2014; Sellier *et al*, 2017). Moreover, sequence manipulations that suppress RAN translation of FMRpolyG largely preclude CGG repeat‐associated toxicity in overexpression systems (Todd *et al*, 2013; Sellier *et al*, 2017). RAN translation is selectively activated by cellular stress response pathways that typically preclude translational initiation, suggesting that specific translational factors or alternative mechanisms may underlie RAN translation and its contributions to repeat‐associated toxicity (Green *et al*, 2017; Cheng *et al*, 2018; Sonobe *et al*, 2018; Westergard *et al*, 2019). Indeed, unbiased and targeted genetic approaches have identified potential factors that preferentially modulate RAN translation including ribosomal protein RPS25 and RNA helicase DDX3X (Cheng *et al*, 2019; Linsalata *et al*, 2019; Yamada *et al*, 2019).

One potential confounder from studies of repeat RNA binding proteins in FXTAS and other repeat expansion disorders to date is their reliance on *in vitro* repeat RNA capture methodologies (Jazurek *et al*, 2016). As most RNAs come to interact with specific RBPs during transcription, export, and/or translation as part of their normal life cycle in the cell, we reasoned that critical factors involved in RAN translation, repeat RNA transport, and RNA foci formation/RBP sequestration might be missed with *in vitro* assays, which do not capture these interactions with great fidelity. It is also possible that interactions of specific factors with CGG repeat RNA may only occur in particular cellular states, such as after activation of cellular stress pathways. Dynamic RNA–protein and RNA–RNA interactions change under cellular stress (Van Treeck & Parker, 2018; Matheny *et al*, 2020) and repeat RNAs such as ALS/FTD‐associated C9ORF72 GGGGCC repeats can partition into stress granules and interact with specific proteins (Fay *et al*, 2017). Thus, capturing context‐specific repeat RNA–protein interactions might reveal novel modulators of repeat RNA biology and pathogenesis.

To define the roles played by CGG repeat RNA binding proteins in both RAN translation and FXTAS pathogenesis *in vivo*, we developed a repeat RNA‐tagging system, which allows for the unbiased identification of RNA‐binding proteins inside cells (Harlen & Churchman, 2017). We fused a pathogenic CGG repeat expansion containing reporter with PP7 viral stem‐loops, which bind to viral coat protein with high affinity (Chao *et al*, 2008). By co‐expressing an epitope‐tagged coat‐binding protein, PCP, we were able to isolate CGG repeat RNAs and associated RBPs. This modular system can be utilized in the context of cellular perturbations such as stress induction or drug treatment. Using this technique, we found that multiple serine/arginine‐rich splicing factor (SRSF) proteins interact with CGG repeat RNAs under normal and stress conditions. Genetic targeting of serine/arginine proteins suppresses rough eye phenotypes and extends survival in a *Drosophila* model of FXTAS. Moreover, genetic or chemical targeting of serine/arginine protein kinases (SRPK1) that regulate SRSF1 function and cellular distribution selectively suppress RAN translation and toxicity in fly and rodent neuronal models of FXTAS through both direct effects on translation and through nuclear CGG repeat RNA retention. Taken together, these data present a novel approach to identify repeat RNA‐binding proteins *in vivo* and establish SR protein kinases as a possible target to modulate RAN translation and repeat expansion‐associated toxicity.

## Results

### Development of a CGG repeat RNA‐tagging system

**Figure EV1:**
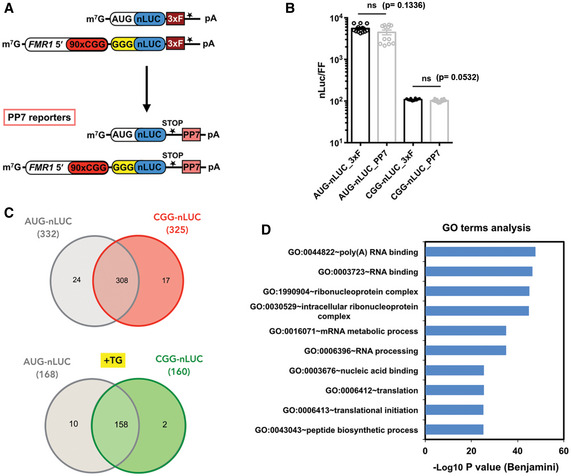
Reporters used for RNA‐tagging experiments and proteins identified by mass spectrometry ASchematic of PP7‐tagged RNA reporters used in this study and the previously published 3x FLAG (3xF)‐tagged reporters that were used to develop the PP7 reporters.BRelative nanoluciferase (nLuc) expression from PP7‐tagged reporters compared to the primary 3x FLAG (3xF)‐tagged reporters showed no significant differences in translational efficiency in HEK293T cells (*n* = 12 biological replicates). Two‐tailed Student’s *t*‐test with Welch’s correction is used for statistical analysis.CVenn diagrams indicate total number of proteins identified for AUG‐nLuc and CGG‐nLuc RNA‐tagging reporters without or with TG treatment (TG^+^).DGO term analysis of manually curated differentially enriched CGG‐interacting proteins. Source data are available online for this figure.

**Figure 1:**
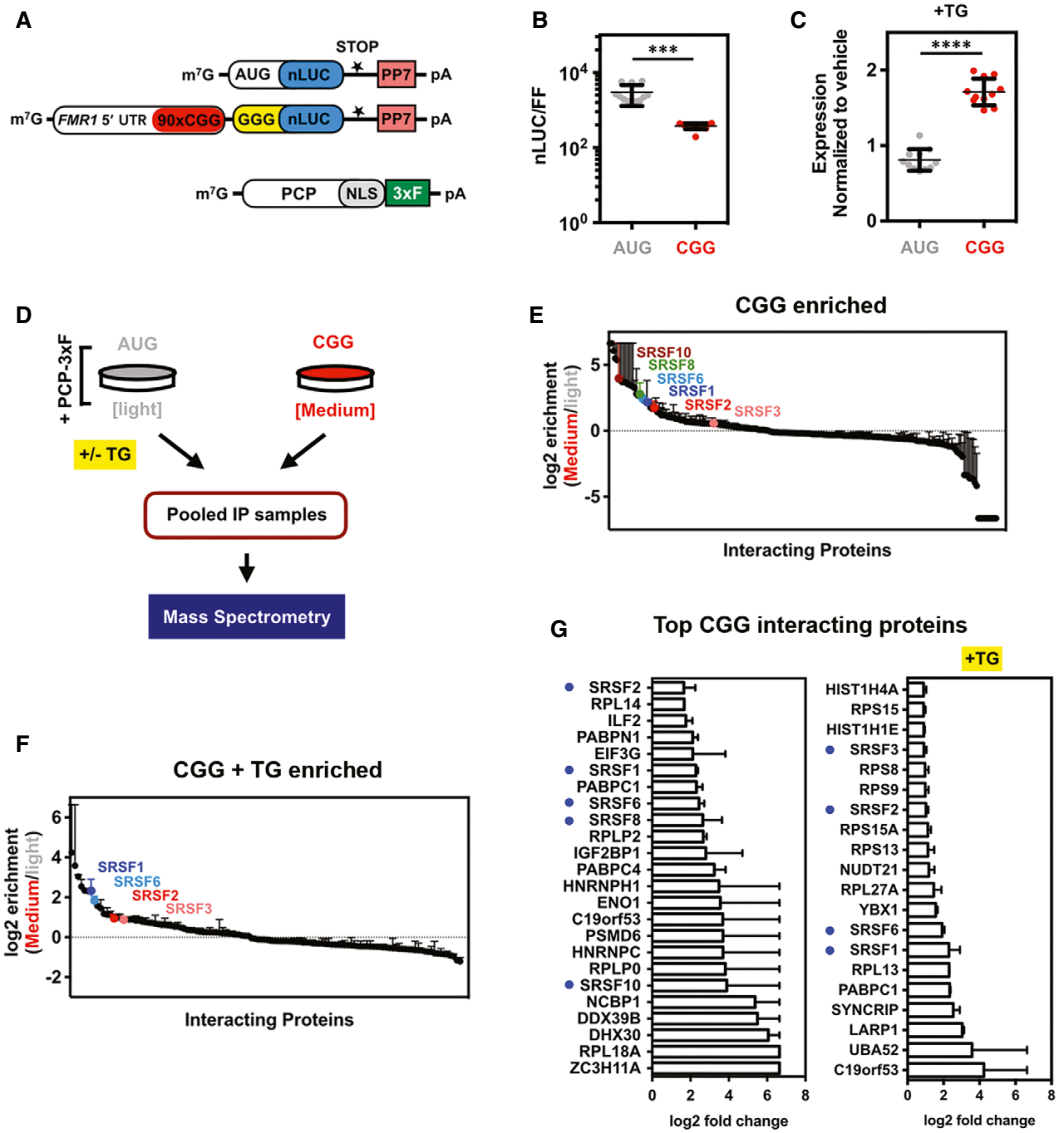
Repeat RNA‐tagging system enables identification of CGG repeat specific RNA‐binding proteins within cells ASchematic of PP7‐tagged RNA reporters and PCP‐NLS‐FLAG constructs used in this study. Details of reporter sequences are described in Appendix Table S1.BRelative protein expression from PP7‐tagged CGG‐nLuc reporters compared to AUG‐driven reporters in HEK293T cells (*n* = 12 biological replicates).CExpression of AUG‐control and CGG‐nLuc RAN translation reporters in HEK293T cells treated with the ER stress agent thapsigargin (TG, 2 μM) (*n* = 11 biological replicates) normalized to vehicle (DMSO).DSchematic of immunoprecipitation and mass spectrometry experiments aimed at identifying CGG repeat RNA‐interacting proteins (see Materials and Methods for details). Growth media, containing different isotopes of lysine and arginine for SILAC labeling, are indicated as light (Lys‐0 and Arg‐0) and medium (Lys‐4 and Arg‐6).E, FLog_2_ fold‐change of the CGG‐interacting protein enrichment compared AUG reporter (*n* = 2 independent experiments; error bars represent range between repeats) under normal (E) and after integrated stress response activation by TG (F).GList of top proteins enriched in CGG‐PP7 RNA interaction compared to AUG in mass spectrometry experiments without or with TG treatment (TG^+^). Bar graphs represent average of two biological replicates ± range. Enriched SR proteins are marked with blue dots. Data information: For graphs in (B and C), error bar represents mean ± SD. Statistical analysis was performed using two‐tailed Student’s *t*‐test with Welch’s correction, ***P < 0.001; ****P < 0.0001. Source data are available online for this figure.

Cellular RNA‐binding proteins (RBPs) play critical roles in RNA gain‐of‐function toxicity in repeat expansion disorders. To identify RBPs that interact with expanded CGG repeat RNAs and that may modulate RAN translation in a FXTAS disease model, we designed an RNA‐tagging system that allowed isolation and identification of CGG repeat RNA‐binding proteins inside cells (Harlen & Churchman, 2017). To accomplish this, we modified previously characterized CGG RAN translation‐specific nanoluciferase (nLuc) reporters by inserting PP7 viral stem‐loops after the stop codon (Fig EV1A, Appendix Table S1 and Fig 1A) (Kearse *et al*, 2016). This reporter system allows translation of the CGG RAN reporter, while keeping the PP7 stem‐loop structures unperturbed to interact with the PP7 coat protein (PCP) (Figs EV1B and 1B and C). While testing the translation efficiency of this PP7‐tagged reporter we found that, consistent with previous findings, CGG RAN translation was significantly less efficient than AUG‐driven canonical translation (Fig 1B) (Kearse *et al*, 2016). Moreover, as expected, CGG RAN translation was enhanced with activation of integrated stress response by thapsigargin (TG) treatment (Fig 1C) (Green *et al*, 2017). Of note, addition of PP7 stem‐loops did not perturb the predicted secondary structure formed by the expanded CGG repeat RNA construct used in this study, indicating that the PP7 stem‐loops should not preclude RBPs from interacting with expanded CGG repeats (Appendix Fig S1). This PP7‐tagged construct was co‐transfected with PP7 coat‐binding protein containing a nuclear localization signal and a 3xFLAG epitope tag (PCP‐NLS‐3xFLAG), which facilitated immunoprecipitation (IP) using anti‐FLAG antibody (Fig 1D).

To quantitatively identify ribonucleoprotein complexes formed by the CGG‐nLuc reporter, we used SILAC (stable isotope labeling by amino acids in cell culture) with HEK293T cells grown in light and medium amino acids before IP (Fig 1D) (Ong *et al*, 2002). We used an AUG‐nLuc –PP7 as control and a GGGGCC‐nLuc‐PP7 for comparative analysis (see Methods and for details). All these reporters have been characterized extensively for their RAN translation efficiency earlier (Kearse *et al*, 2016; Green *et al*, 2017; Linsalata *et al*, 2019). Here, we used these reporters as bait to capture specific proteins that may interact with these reporters and involved in RNA export and/or translation processes. Importantly, for this study we mainly analyzed the CGG interactome, as the GGGGCC interactome will be the focus of a separate manuscript (see Methods and Data Set EV1). For SILAC, AUG‐nLuc‐PP7 reporter was grown in light and CGG‐nLuc‐PP7 reporter was grown in medium amino acids containing HEK293T cells and both of them were co‐transfected with the 3xFLAG tagged PCP (Fig 1D). In parallel, to determine RBPs that may interact with these reporters after ISR activation, cells were treated with 2 μM TG 5 h before the IP. The SILAC analysis provided more than 300 protein interactors with quantification of their binding preferences for AUG and CGG reporters (Figs 1E and EV1C). Fewer interactors were identified after ISR activation with TG treatment (Figs 1F and EV1C). A reduction in RNA–protein interaction during integrated stress response might be a result of perturbation in normal RNA metabolism during cellular stress (Bond, 2006).

### SR proteins selectively interact with CGG repeat RNAs

Gene ontology (GO) enrichment analysis of manually curated CGG‐enriched protein interactors using the Database for Annotation, Visualization and Integrated Discovery (DAVID) yielded top functional categories related to poly(A) RNA‐binding, RNA‐binding, ribonucleoprotein complexes, mRNA metabolic processes, RNA processing, and translation (Fig EV1D), indicating that this RNA‐tagging IP successfully captured RBPs that may differentially interact with CGG repeat RNA during its synthesis, transport, and translation (Dennis *et al*, 2003). As expected, previously identified CGG interactors hnRNPA2B1 or Sam68 (KHDRBS1) interacted with our CGG reporter (Data Set EV1). Both AUG‐initiated and CGG‐repeat constructs formed similar ribonucleoprotein complexes and exhibited significant overlap in their interactomes (Fig EV1C). However, several RBPs including multiple serine/arginine‐rich domain (SR) proteins preferentially interacted with CGG repeat reporters compared to the AUG reporter (Fig 1E–G).

**Figure 2:**
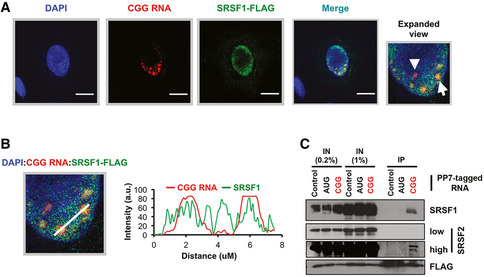
HCR‐ICC detects co‐localization of SRSF1 and CGG repeat RNA AHybridization chain reaction (HCR) detection of CGG repeat RNA along with immunocytochemistry (ICC) to detect SRSF1‐FLAG in U2OS cells. Expanded view of merged channels shows the CGG RNA foci (arrowhead) and an example of SRSF1‐CGG RNA foci co‐localization (arrow). DAPI marks the nucleus. Scale bars are 10 µm.BCo‐localization analysis of CGG RNA (red traces) and SRSF1 (green traces) showing overlaps of CGG RNA and SRSF1. (a.u. = arbitrary unit).CCo‐immunoprecipitation of indicated SRSF proteins with PP7‐tagged control sequence (partial nLUC sequence) to correct for non‐specific binding, AUG, and CGG reporter RNAs. SRSF1 and 2 specifically immunoprecipitated with PP7‐tagged CGG reporter. Source data are available online for this figure.

Top RBPs that preferentially interact with CGG repeat reporter in basal condition (no stress) include heterogeneous nuclear ribonucleoproteins hnRNPH, hnRNPC; poly(a) binding protein PABPN1, PABPC1, and PABPC4; ribosomal proteins RPL18A, RPLP0, and RPLP2 (Fig 1G). Stress‐specific interactors include ubiquitin‐60S ribosomal protein L40 (UBA52), hnRNPQ, PABPC1, LARP1, and YBX1 (Fig 1G). Interestingly, multiple SR proteins interact with the CGG reporter basally and in response to cellular stress (Fig 1E–G). SR proteins are a large family of RBPs consisting 12 structurally related proteins containing characteristic Arg/Ser‐rich (RS) domains that influence mRNA splicing, export, stability, and translation (Zhou & Fu, 2013). To validate some of these SR protein interactions, we took a two‐pronged approach. First, we determined the co‐localization of a key SR protein, SRSF1, with CGG repeat RNA by hybridization chain reaction (HCR) and immunocytochemistry (ICC). HCR enabled the detection of CGG repeat RNA foci in transfected U2OS cells and showed co‐localization of the RNA with SRSF1 (Fig 2A and B). In a parallel approach, we immunoprecipitated PP7‐tagged reporter RNAs and immunoblotted for SRSF proteins. SRSF1 interacted very strongly with PP7‐tagged CGG repeat RNA reporter compared to a PP7‐tag control or AUG reporter (Fig 2C). SRSF2 also interacted preferentially with the CGG RNA reporter, albeit less robustly than SRSF1 (Fig 2C).

### SR proteins modulate CGG and GGGGCC repeat RNA toxicity in *Drosophila*

**Figure 3:**
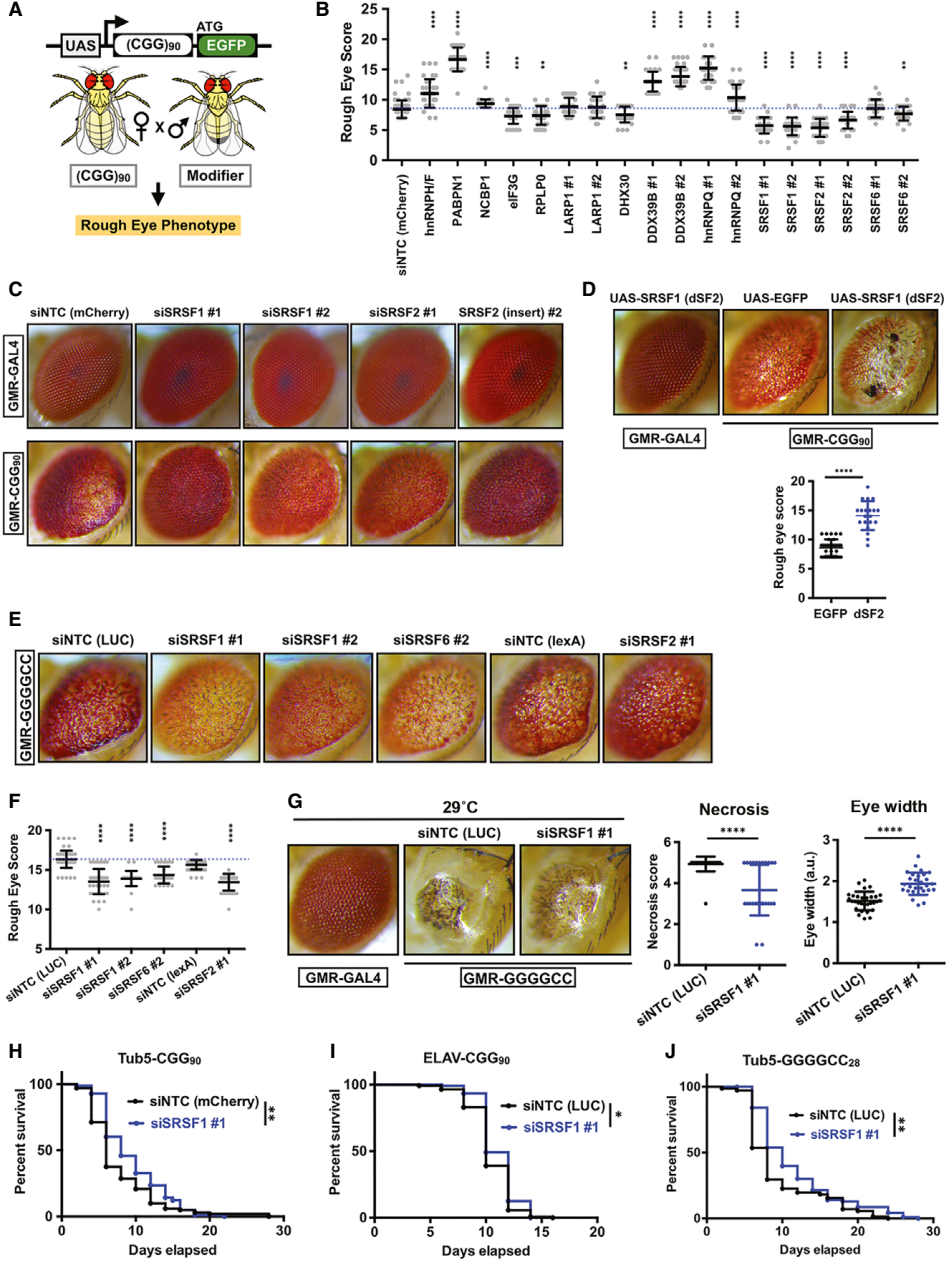
SRSF proteins act as modifiers of CGG and GGGGCC repeat‐associated toxicity in *Drosophila* ASchematic of (CGG)90‐EGFP construct and experimental outline for rough eye phenotype screening.BQuantitation of GMR‐GAL4‐driven uas‐(CGG)90‐EGFP eye phenotype with candidate modifiers (*n* ≥ 30 flies/genotype). siNTC = siRNA against a non‐targeting control gene (mCherry). Different siRNA lines for the same target gene are numbered (#1 and #2). Error bars represent mean ± SD.CRepresentative photographs of fly eyes expressing either GMR‐GAL4 driver alone or with uas‐(CGG)90‐EGFP construct, with fly SRSF1 (dSF2) and SRSF2 (dSC35) knockdown or disruptions (insertion).DRepresentative photographs of fly eyes and quantitation (below) of rough eye scores with fly SRSF1 overexpression (dSF2 OE); *n* = 20–32/genotype. Error bars represent mean ± SD.ERepresentative photographs of fly eyes expressing GMR‐GAL4‐driven (GGGGCC)28‐EGFP with indicated uas‐siRNAs against fly SRSF genes in comparison with non‐targeting control (NTC) siRNA against LUC/luciferase.FQuantitation of (GGGGCC)28‐EGFP rough eye phenotype with SRSF modifiers (*n* ≥ 30 flies/genotype). Error bars represent mean ± SD.GRepresentative photographs of fly eyes expressing GMR‐GAL4‐driven (GGGGCC)28‐EGFP at 29°C along with the quantifications of necrosis and eye width. *n* = 28–30/genotype. Error bars represent mean ± SD.H, ISurvival assays of flies expressing (CGG)90‐EGFP under Tub5‐GS (H) and ELAV‐GS (I) drivers with control or SRSF1 siRNAs. Expression of (CGG)90‐EGFP was initiated with addition of drug starting 1 day post‐eclosion and continued through experiment (Log‐rank Mantel–Cox test; *n* = 98–101/genotype for Tub‐GS and *n* = 120–141/genotype for ELAV‐GS flies); *P < 0.05, **P < 0.01.JSurvival assays of (GGGGCC)28‐EGFP expressing fly under Tub5‐GS driver (Log‐rank Mantel–Cox test; *n* = 71–93/genotype) with control or SRSF1 siRNAs. **P < 0.01. Data information: For eye scoring, target siRNA lines were compared to non‐targeting control siRNA lines using a two‐tailed Student’s *t*‐test with Welch’s correction for multiple comparisons. **P < 0.01; ***P < 0.001; ****P < 0.0001. Human orthologs of fly genes are used for labeling. Details of fly genes are described in Appendix Table S2. Source data are available online for this figure.

**Figure EV2:**
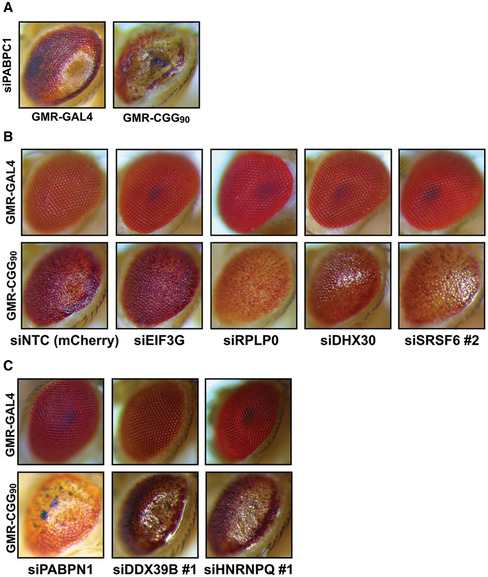
Modifiers of CGG repeat‐associated toxicity in *Drosophila* ARepresentative photographs of fly eyes expressing either GMR‐GAL4 driver alone or the (CGG)90‐EGFP construct under a GMR‐GAL4 driver, with siRNA against fly homologs of PABPC1.B, CRepresentative examples of (B) suppressors (eIF3G, RPLP0, DHX30, and SRSF4/6) and (C) enhancers (PABPN1, DDX39B, and hnRNPQ/Syncrip) of CGG repeat toxicity.

To test whether any of the CGG repeat RNA interactors can modulate CGG RNA toxicity, we conducted a candidate‐based screen using a *Drosophila melanogaster* model of FXTAS (Fig 3A and B) (Todd *et al*, 2013; Linsalata *et al*, 2019). This fly model carries an upstream activation sequence (UAS)‐driven 5’ UTR of human *FMR1* with 90 CGG repeats fused to EGFP in the +1 (FMRpolyG) reading frame. Expression of this reporter in the fly eye via a GMR‐GAL4 driver results in a rough‐eye phenotype (Todd *et al*, 2013). We have previously used this fly model to screen for modifiers of *FMR1* CGG RAN translation and identified several translation‐associated factors that modulate CGG repeat toxicity (Linsalata *et al*, 2019). For the modifier screen in this study, we selected a few candidates from the list of top CGG‐interacting proteins (Fig 1G; Appendix Table S2) to cross with GMR‐GAL4 control and 90 CGG repeats expressing flies. Candidate modifiers with intrinsic toxicity were excluded from further analysis (Fig EV2A). We found that knocking down *Drosophila* homologs of several SR proteins (SRSF1, 2, and 6) as well as translation initiation factor eIF3G, ribosomal protein RPLP0, and RNA helicase DHX30 significantly reduced CGG repeat RNA toxicity (Figs 3B and C, and EV2B). Factors whose knockdown significantly enhanced CGG repeat toxicity in fly eye include fly homologs of heterogeneous nuclear ribonucleoproteins hnRNP H/F and hnRNPQ/Syncrip; poly(a) binding protein (PABPN1); nuclear cap‐binding protein (NCBP1); and DExD‐Box Helicase 39B (Figs 3B and EV2C).

To investigate whether overexpression of SR proteins might enhance CGG repeat toxicity, we developed a transgenic *Drosophila* model carrying UAS‐driven dSF2 (*Drosophila* homolog of human SRSF1) and expressed this reporter in the fly eye using a GMR‐GAL4 driver. Expression of dSF2 alone did not lead to any eye abnormalities. However, co‐expression of dSF2 significantly enhanced CGG repeat elicited eye degeneration compared to an eGFP control (Fig 3D). A recent study has shown that SRSF1 is required for nuclear export of GGGGCC repeats and knockdown of SRSF1 modulates GGGGCC repeat toxicity in a fly model (Hautbergue *et al*, 2017). Consistent with this published work, in our mass spectrometry experiments multiple SRSF proteins interacted with the GGGGCC reporter (Data Set EV1). Thus, we asked if multiple SR proteins may modulate GGGGCC repeat toxicity in a *Drosophila* model. To this end, we used a transgenic Drosophila model expressing UAS‐driven 28 GGGGCC repeats that exhibits a rough‐eye phenotype when expressed in fly eyes (He *et al*, 2020). We found that *Drosophila* homologs of several SR proteins, including SRSF1, SRSF2, and 6, significantly reduced rough‐eye phenotypes in the GGGGCC repeat expressing fly (Fig 3E and F). Moreover, knock down of SRSF1 significantly reduced severe rough eye phenotype observed at higher temperature (29°C) that causes necrosis and shrinkage of GGGGCC repeat expressing fly eyes (Fig 3G).

**Figure EV3:**
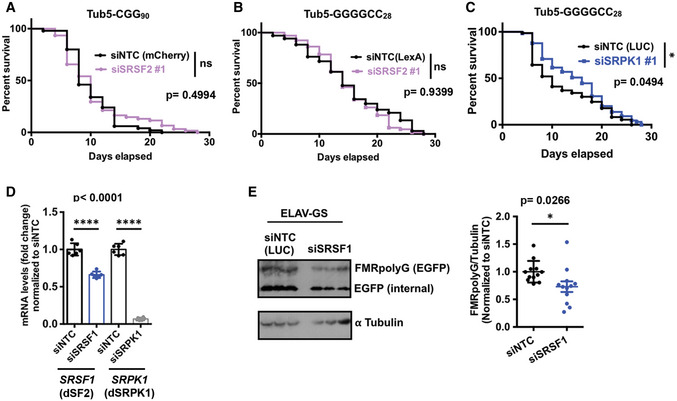
Impact of SRSF and SRPK modulation on RAN translation and survival assays in *Drosophila* A–CSurvival assays of (CGG)90‐EGFP and (G4C2)28‐EGFP expressing fly under Tub5‐GS driver with respective siRNAs as mentioned. Log‐rank Mantel–Cox test; *n* = 50–61 (A), 65–67 (B); and 65–73 (C). *P < 0.05.D
*Drosophila* SRSF1 (dSF2, blue bar) and SRPK1 (gray) levels after siRNA knockdown as compared to non‐targeting siRNA (siNTC, black bars), quantified by qRT–PCR. Error bars represent mean ± SD RNAs from two (*n* = 2 biological repeats) independent fly crosses (20–25 flies/genotype per cross) used to run RT–qPCR in triplicates (3 technical replicates). All data points presented in the graphs.EWestern blot of FMR‐polyG‐EGFP RAN products in (CGG)90‐EGFP/ELAV‐GS flies with or without SRSF1 knockdown. Error bars represent mean ± SD. Total protein from three (*n* = 3 biological repeats) independent fly crosses (20–22 flies/genotype per cross) and run in four replicates per gel (technical replicates). All data points presented in the graphs. Data information: Statistical analysis in (D) and (E) is performed using Student’s *t*‐test with Welch’s correction. *P < 0.05, ****P < 0.0001.

Ubiquitous expression of 90 CGG repeats after eclosion in adult flies using the Tub5‐Geneswitch system significantly shortens lifespan (Todd *et al*, 2010; Linsalata *et al*, 2019). Conversely, we observed that expression of siRNAs against SRSF1 under an inducible Tub5‐Geneswitch driver modestly increased the lifespan of flies expressing 90 CGG repeats (Fig 3H). Similarly, SRSF1 knockdown increased lifespan when CGG repeat was expressed selectively within neurons in adult flies under an inducible Geneswitch ElaV driver (Fig 3I). However, siRNAs against SRSF2 failed to enhance survival of flies expressing 90 CGG repeats (Fig EV3A). Together, these results suggest that SRSF1 in particular plays a role in adult‐onset CGG repeat‐associated neurodegeneration in *Drosophila*. As with CGG repeats, ubiquitous expression of siRNA against SRSF1 led to a modest but statistically significant enhancement of survival of GGGGCC repeat expressing fly, but SRSF2 did not show any significant change (Figs 3J and EV3B). Taken together, these results are consistent with the earlier findings related to SRSF1 and GGGGCC repeats (Hautbergue *et al*, 2017), while extending the results to a second disease‐causing repeat expansion (CGG) and suggesting that other SR proteins can also be involved in repeat expansion‐associated toxicity.

### SRSF Protein Kinase 1 (SRPK1) modifies repeat RNA toxicity in *Drosophila*

**Figure 4:**
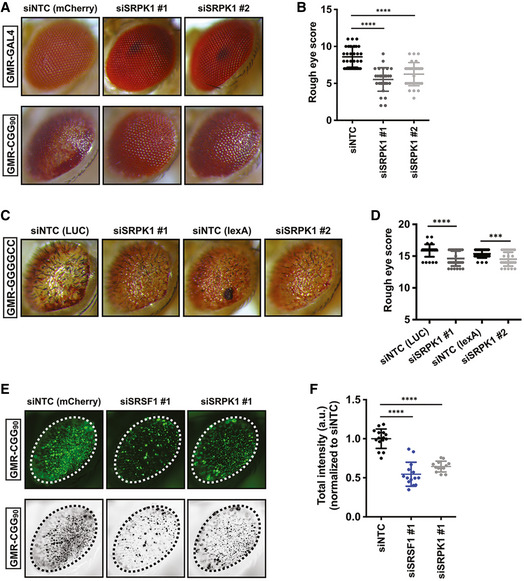
SRPK1 knockdown modifies CGG and GGGGCC repeat‐associated toxicity in *Drosophila* A, BRepresentative (A) photographs of fly eyes and (B) quantitation with siRNA‐mediated knockdown of SRPK1 (dSRPK1); *n* = 30–34/genotype. Error bars represent mean ± SD.CRepresentative photographs of fly eyes expressing GMR‐GAL4‐driven (GGGGCC)28‐EGFP with siRNA‐mediated knockdown of SRPK1 or disruption by insertion.DQuantitation of rough eye phenotypes. *t*‐test with Welch corrections for comparisons with the control; *n* = 31–34 flies/ genotype. Error bars represent mean ± SD.ERepresentative external eye imaging for the detection of GFP aggregates caused by (CGG)90‐EGFP transgene expression (top). Converted images used to quantify total intensity of GFP puncta (bottom).FDepletion of SRSF1 or SRPK1 by RNAi results in reduced (CGG)90‐EGFP puncta compared to control siRNA as quantified by total intensity (a.u. = arbitrary unit). *n* = 13–15 flies/genotype. Error bars represent mean ± SD. Data information: Statistical analysis was performed using two‐tailed Student’s *t*‐test with Welch’s correction, ***P < 0.001; ****P < 0.0001. Source data are available online for this figure.

Serine–arginine protein kinases (SRPKs 1–3) selectively phosphorylate SR proteins and modulate their subcellular localization (Zhou & Fu, 2013). In addition, many non‐splicing functions for SRPKs have been reported, including roles in tau phosphorylation and Alzheimer’s disease (AD) pathogenesis (Hong *et al*, 2012) and in translational regulation (Brown *et al*, 2014). SRPKs exhibit highly tissue‐specific expression profiles, suggesting that these kinases may have specialized functions (Wang *et al*, 1998; Nakagawa *et al*, 2005). SR proteins are misregulated in multiple cancers, and pharmacological targeting of the SRPK1‐SR axis has been proposed as a potential therapeutic approach (Amin *et al*, 2011; Mavrou *et al*, 2015; Mavrou & Oltean, 2016). As multiple SR proteins modulate both CGG and GGGGCC repeat RNA toxicities in our fly models, we asked if SRPK1 may also affect repeat RNA toxicity in flies. To this end, first we tested the effects of altering expression of SRPK1 on CGG repeat RNA toxicity in our FXTAS fly model. Interestingly, we found that selective knockdown of the *Drosophila* homolog of SRPK1 (dSRPK1) reduced CGG repeat RNA toxicity and significantly improved the CGG repeat rough eye phenotype (Fig 4A and B). Next, we asked if altering SRPK1 expression can also reduce GGGGCC repeat RNA toxicity in our fly model. Indeed, expression of siRNA against SRPK1 (dSRPK1) reduced GGGGCC repeat toxicity and significantly improved the associated rough eye phenotype (Fig 4C and D). Additionally, ubiquitous expression of siRNAs against dSRPK1 under an inducible Tub5‐Geneswitch driver led to a modest but statistically significant effect on lifespan of flies expressing GGGGCC repeats (Fig EV3C). We also confirmed that improvement of CGG RNA toxicity in *Drosophila* occurs due to decreased expression of target genes by siRNA‐mediated knockdown (Fig EV3D). Together, these results suggest that SRPK1 can modify repeat expansion‐associated toxicity, either directly through modulating SR proteins or through independent pathways.

Amelioration of CGG RNA toxicity by SRSF1 and SRPK1 knockdown might occur through altering the levels of RAN products in *Drosophila*. To test this possibility, first we assessed if any of the modifiers can decrease GFP inclusions (FMRpolyG‐EGFP) in fly eyes. A previous study from our laboratory has shown that RAN translation of the CGG90‐EGFP reporter can form GFP inclusions in *Drosophila* eye, while expression of GFP alone do not form inclusions (Todd *et al*, 2010). siRNA‐mediated knockdown of either SRSF1 or SRPK1 significantly reduced GFP inclusions in eye compared to control siRNA (Fig 4E and F). In addition, FMRpolyG‐EGFP levels in adult flies expressing CGG repeats within neurons were modestly decreased by siRNA‐mediated knockdown of SRSF1 (Fig EV3E). Together, these results suggest that SRSF1 and SRPK1 modify CGG repeat‐associated toxicity in *Drosophila* through altering RAN protein levels.

### SRPK1 inhibitors modulate RAN translation in cell‐based reporters

**Figure 5:**
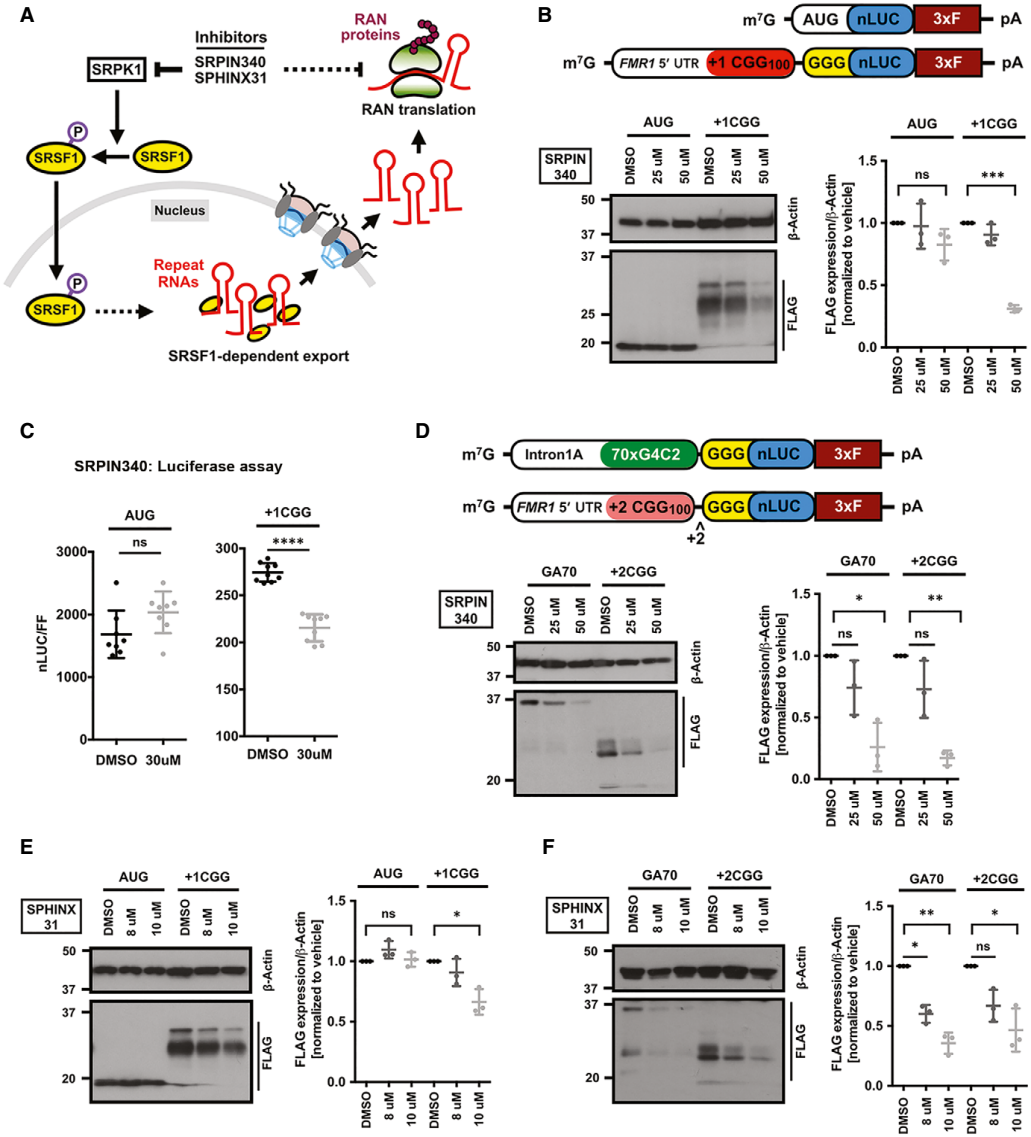
SRPK1 inhibitors selectively suppress RAN translation ASchematic of SRPK1 signaling pathway that regulates subcellular SRSF1 localization, which in turns can impact export and translation of repeat RNAs in the cytoplasm. SRPK1 may also act directly on protein translation pathways (dotted arrow). Known pharmacological compounds that inhibit SRPK1 can disrupt this pathway.BAnti‐FLAG immunoblot of DMSO and SRPIN340 pre‐treated HEK293T cells expressing AUG‐nLuc‐3xFLAG control or CGG‐nLuc‐3xFLAG RAN translation reporters. β‐Actin is used as a loading control. To prevent signal saturation, AUG‐nLuc lysate was diluted 1:3 in sample buffer prior to loading (*n* = 3 biological replicates). Schematics of the AUG‐nLUC‐3xFLAG and +1CGG(100)‐nLuc‐3xFLAG reporters presented on top.CRelative expression of AUG‐nLuc and CGG‐nLuc reporters in HEK293T cells (*n* = 8–9 biological replicates) following treatment with DMSO and SRPIN340.DAnti‐FLAG immunoblot of DMSO and SRPIN340 pre‐treated HEK293T cells expressing GGGGCC‐nLuc‐3xFLAG (GA70) and +2CGG‐nLuc‐3xFLAG (FMRpolyA) RAN translation reporters (*n* = 3 biological replicates). Schematics of the GA70 (GGGGCCx70) and +2CGG reporters presented on top.EImmunoblot of DMSO and SPHINX31 pre‐treated HEK293T cells expressing AUG‐nLuc‐3xFLAG control or CGG‐nLuc‐3xFLAG RAN translation reporters (*n* = 3 biological replicates).FAnti‐FLAG of DMSO and SPHINX31 pre‐treated HEK293T cells expressing GGGGCC‐nLuc‐3xFLAG (GA70) and +2CGG‐nLuc‐3xFLAG (FMRpolyA) RAN translation reporters (*n* = 3 biological replicates). Data information: Error bars represent mean ± SD. Statistical analysis was performed using two‐tailed Student’s *t*‐test with Welch’s correction. *P < 0.05; **P < 0.01, ***P < 0.001, and ****P < 0.0001. To prevent over‐exposure, the AUG‐nLuc lysate was diluted 1:3 in sample buffer. Source data are available online for this figure.

Since genetic ablation of *Drosophila* homolog of SRPK1 altered RAN protein (FMRpolyG‐EGFP) levels in a fly model of FXTAS, we next asked if inhibition of SRPK1 can modulate RAN translation in mammalian cells. We hypothesized that SRPK1 is required for SR protein‐mediated export of repeat RNAs into the cytoplasm (Fig 5A). Thus, SRPK1 inhibition can possibly lead to a decrease in cytoplasmic levels of repeat RNAs, resulting ultimately in a decrease level of RAN translation. To monitor RAN translation in mammalian cells, we used our previously characterized nLuc‐based CGG RAN translation reporter consisting of a 3xFLAG‐tagged nanoluciferase (nLuc‐3XF) downstream of the 5’ UTR of human *FMR1* (Kearse *et al*, 2016). In order to test the effects of SRPK1 inhibition on RAN translation, we used two known chemical inhibitors of SRPK1 (Fig 5A). First, we tested the effects of SRPIN340, an ATP‐competitive SRPK inhibitor, on RAN translation by pre‐treating HEK293T cells with SRPIN340 followed by transfecting CGG RAN translation reporters (Fukuhara *et al*, 2006). SRPIN340 treatment at 50 μM led to a significant and selective decrease in +1CGG RAN translation as detected by immunoblot (Fig 5B). Similarly, SRPIN340 treatment at 30 μM inhibited CGG RAN translation as measured by nanoluciferase assay, but this had no impact on AUG‐nLuc expression (Fig 5C). The requirement of lower concentration of SRPIN340 treatment for RAN inhibition in nanoluciferase assays compared to Western, is possibly due the differences in the sensitivity of detection between these assays. The effects of SRPIN340 was not isolated to FMRpolyG synthesis: We showed that SRPK1 inhibition by SRPIN340 also suppressed GGGGCC RAN translation (GA70) and +2CGG RAN translation (FMRpolyA) using previously published reporters (Fig 5D) (Kearse *et al*, 2016; Green *et al*, 2017).

As pharmacological agents may have off‐target effects, we also evaluated the impact of a second SRPK inhibitor, SPHINX31 (Gammons *et al*, 2013; Batson *et al*, 2017). Similar to SRPIN340, SPHINX31 significantly and selectively inhibited +1CGG, GGGGCC and +2CGG RAN translation (Fig 5E and F). These results indicate that pharmacological inhibition of SRPK1 has a general inhibitory effect on RAN translation.

### SRPK1 inhibitors prohibit stress‐induced increase in RAN translation

**Figure 6:**
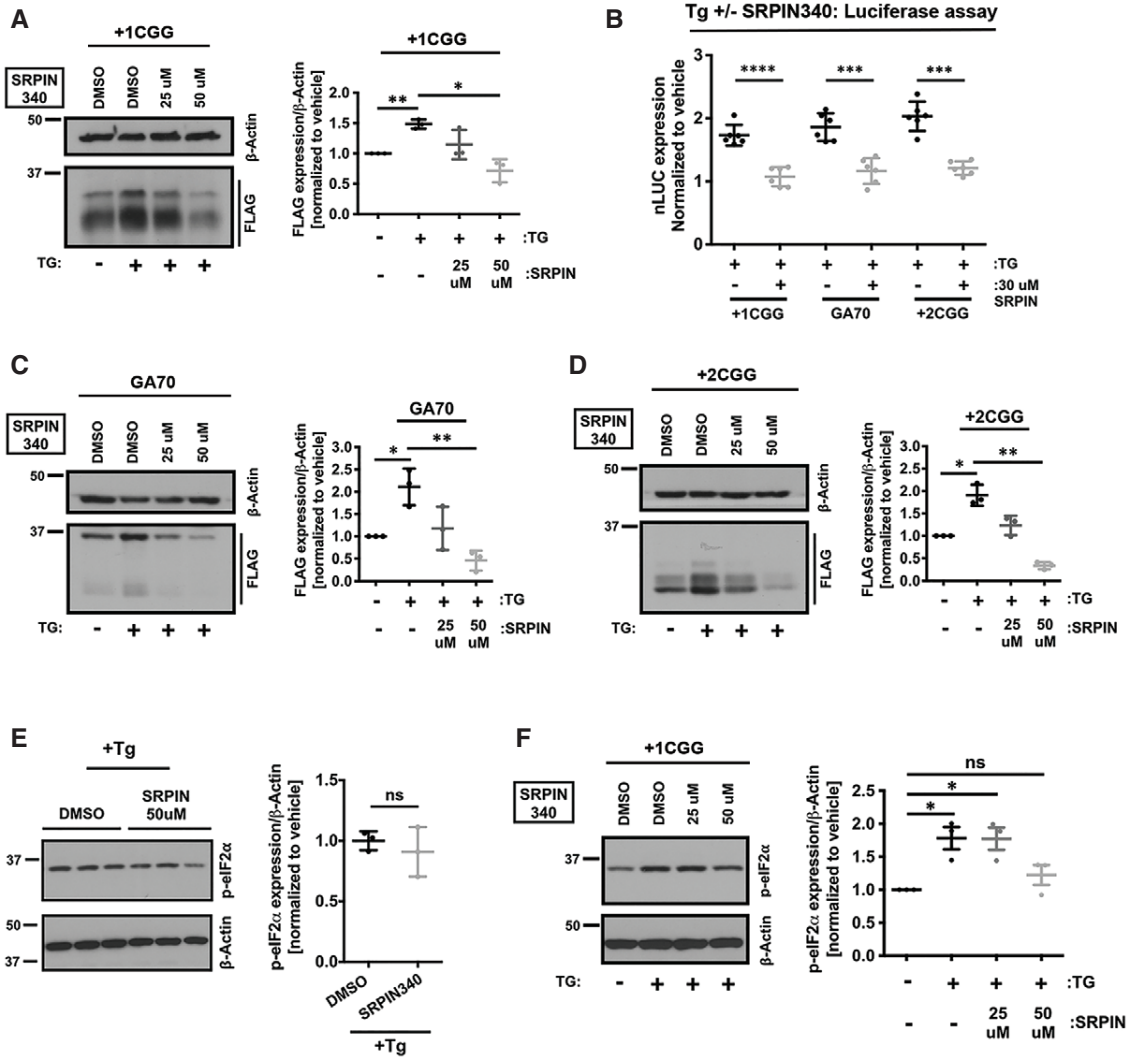
SRPK1 inhibition prevents stress‐induced enhancement of RAN translation AExpression of +1CGG‐nLuc‐3xFLAG RAN translation reporters in HEK293T cells treated with 2 μM TG (for stress induction) analyzed by immunoblot (*n* = 3 biological replicates). To evaluate effects of SRPK1 inhibition, cells were pre‐treated with DMSO or SRPIN340 before reporter transfection.BRelative expression of +1CGG‐nLuc reporters in HEK293T cells (*n* = 6 biological replicates) following stress induction with 2 μM TG treatment. Values normalized to vehicle (DMSO) treatment. As in (A), cells were pre‐treated with DMSO or SRPIN340 before reporter transfection.C, DExpression of GGGGCC‐nLuc‐3xFLAG (C) and +2CGG‐nLuc‐3xFLAG (D) RAN translation reporters in HEK293T cells treated with 2 μM TG (for stress induction) analyzed by immunoblot (*n* = 3 biological replicates). To evaluate effects of SRPK1 inhibition, cells were pre‐treated with DMSO or SRPIN340 before reporter transfection.ESRPK1 inhibition by SRPIN340 did not alter eIF2α phosphorylation in response to 2 μM TG (for stress induction) in HEK293T cells by immunoblot (*n* = 3 biological replicates).FSPRK1 inhibition suppressed eIF2α phosphorylation in HEK293T cells treated with 2 μM TG (for stress induction) in the presence of the +1CGG reporter. Cells were pre‐treated with DMSO or SRPIN340 before reporter transfection (*n* = 3 biological replicates). Data information: Error bars represent mean ± SD. Two‐tailed Student’s *t*‐test with Welch’s correction. *P < 0.05; **P < 0.01; ***P < 0.001; and ****P < 0.0001. Source data are available online for this figure.

RAN protein levels increase under various stress conditions, including ER stress (Green *et al*, 2017; Cheng *et al*, 2018; Sonobe *et al*, 2018; Westergard *et al*, 2019). During integrated stress response (ISR), phosphorylation of eIF2α leads to a decrease in global translation, while RAN translation remains unperturbed. This creates a feed‐forward loop that leads to production of more RAN proteins, which contribute to neuronal dysfunction and death. As SPRK1 inhibitors selectively suppressed RAN translation, we wondered if SRPK1 inhibition might also impede stress‐induced enhancement of RAN translation. Pre‐treating cells with 50 μM SRPIN340 led to a complete blockade of thapsigargin‐induced enhancement of +1CGG RAN translation as detected by immunoblot and luciferase assays (Fig 6A and B). SRPIN340 also suppressed stress‐induced GGGGCC (GA70) and +2CGG RAN translation (Fig 6B–D). Together, these results suggested that pharmacological inhibition of SRPK1 can prohibit both basal level and stress‐induced increase in RAN translation across at least two different repeats and at least two reading frames of the CGG repeat.

To assess the mechanism by which SPRK1 inhibitors precluded stress‐induced RAN translation, we asked whether SRPK1 inhibitors directly target cellular ISR pathways. To test this, we measured eIF2α phosphorylation levels in TG‐treated (stress‐induced) cells in the presence or absence of SRPIN340. We did not observe any significant changes in the levels of phosphorylated eIF2α after ISR induction with TG in presence of SRPIN340 (Fig 6E). However, we did observe that 50 μM SRPIN340 treatment lead to a modest blockade in the enhancement of phosphorylated eIF2α during ISR induction in the presence of +1CGG repeat RNA (Fig 6F). We conclude that although SRPIN340 does not directly target ISR pathways alone, it may decrease the overall burden of cellular stress triggered by the presence of +1CGG repeat RNA through inhibiting RAN protein production.

### SRPK1 inhibitors trigger nuclear accumulation of CGG repeat RNAs

Genetic or pharmacological inhibition of SRPK1 could theoretically reduce RAN translation through multiple mechanisms (Fig 5A). SRPK1 inhibition may alter SR protein‐mediated export of repeat RNAs, thus preventing their access to assembled and active ribosomes. Alternatively, SRPK1 inhibition could directly impair RAN translation through either an SR protein dependent or independent pathway. We tested both of these possibilities.

**Figure 7:**
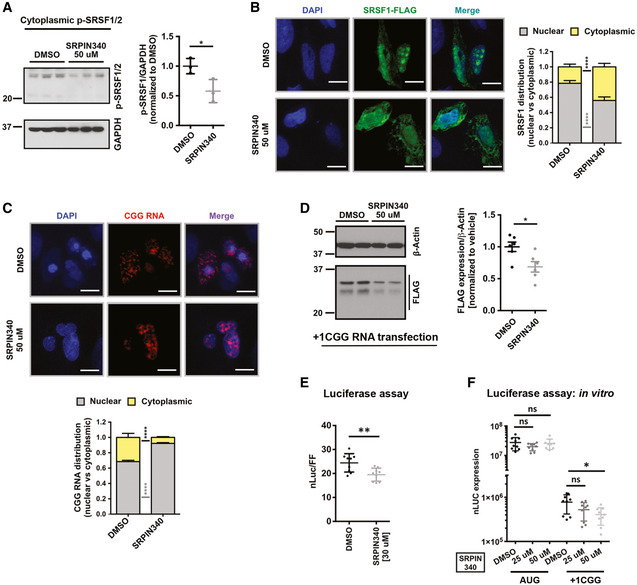
SRPK1 inhibition alters CGG repeat RNA localization and directly inhibits RAN translation AImmunoblot of cytoplasmic phospho‐SRSF1/2 after SRPIN340 treatment. GAPDH is used as the loading control. Error bars represent mean ± SD (*n* = 3 biological replicates). Statistical analysis was performed using Student’s *t*‐test with Welch’s correction. *P < 0.05. Full images of the same blot showing levels of all phospho SR proteins (pan SRSF phosphorylation) after SRPIN340 treatment are presented in Fig EV4A.BICC images of FLAG‐tagged SRSF1 after SRPIN340 treatment compared to vehicle (DMSO). Quantification shows the ratio of nuclear and cytoplasmic intensity of SRSF1 signal (see methods for details). Error bars indicate mean ± 95% CI (*n* = 85–126 cells/condition). Statistical analysis was performed using *t*‐test with Welch’s correction, ****P < 0.0001.CNucleocytoplasmic distribution CGG repeat RNA after SRPIN340 treatment compared to vehicle (DMSO) as detected by HCR. Quantification shows the ratio of nuclear and cytoplasmic intensity of CGG RNA signal (see methods for details) as parts of whole. Error bars indicate mean ± 95% CI (*n* = 124–151 cells/condition). Statistical analysis was performed using *t*‐test with Welch’s correction, ****P < 0.0001.DAnti‐FLAG immunoblot blot of DMSO and SRPIN340 pre‐treated HEK293T cells transfected with *in vitro* transcribed CGG‐nLuc‐3xFLAG reporter RNA. β‐Actin is used as a loading control. Error bars represent mean ± SD (*n* = 6 biological replicates). Statistical analysis was performed using Student’s *t*‐test with Welch’s correction. *P < 0.05.ERelative expression of *in vitro* transcribed CGG‐nLuc reporter in HEK293T cells pre‐treated with DMSO and SRPIN340. Error bars represent mean ± SD (*n* = 9 biological replicates).FExpression of *in vitro* transcribed Aug‐nLuc and CGG‐nLuc reporters in rabbit reticulocyte lysate (RRL) *in vitro* translation system following pre‐treatment with DMSO or SRPIN340. Error bars represent mean ± SD (*n* = 9 biological replicates). Data information: For E and F, statistical analysis was performed using Student’s *t*‐test with Welch’s correction. *P < 0.05; **P < 0.01. Source data are available online for this figure.

**Figure EV4:**
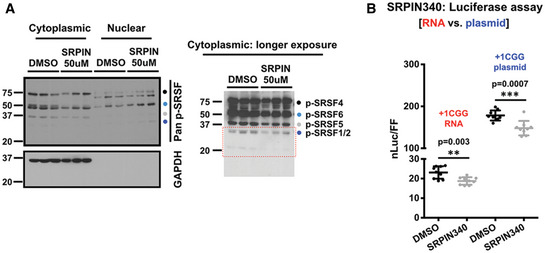
SRPK1 inhibition alters SR protein phosphorylation and inhibits RAN translation ASubcellular levels of phosphorylated SRSF proteins with or without SRPIN340 treatment measured by Western blot using an antibody, which detects phosphorylated SR proteins (p‐SRSFs). GAPDH is used as cytoplasmic marker and loading control for cytoplasmic fraction of phosphorylated SRSFs (*n* = 3 biological repeats). Detected p‐SRSFs are labeled/color‐coded. p‐SRSF1/2 data presented in Fig 7A are outlined in red dots.BRelative nanoluciferase (nLuc) expression of +1 CGG‐nLuc‐3xF reporter transfected as plasmid or as *in vitro* transcribed RNA in presence or absence of SRPIN340 treatment (*n* = 9). Error bars represent mean ± SD (*n* = 12 biological repeats). Statistical analysis is performed using two‐tailed Student’s *t*‐test with Welch’s correction. **P < 0.01, ***P < 0.001.

Inhibition of SRPK1 results in decreased phosphorylation of target SR proteins, particularly SRSF1, resulting in a reduction of SRSF1 nuclear import (Zhou & Fu, 2013; Gonçalves *et al*, 2014). Consistent with this, we found that SRPIN340 treatment significantly decreased levels of phosphorylated SRSF1/2 in the cytoplasm (Figs 7A and EV4A). Likewise, SRPIN340 treatment reduced nuclear SRSF1 protein levels (Fig 7B). If SRSF1 is required for nuclear export of CGG repeat RNAs, as described earlier for GGGGCC repeat RNAs (Hautbergue *et al*, 2017), then a decrease in nuclear SRSF1 levels could impair export of CGG repeat RNAs. Consistent with this prediction, treating cells with SRPIN340 triggered accumulation of CGG repeat RNAs inside the nucleus as measured by HCR (Fig 7C). Together, these results suggest that inhibition of SRPK1 leads to impaired export and nuclear accumulation of repeat RNAs, resulting in decrease production of RAN proteins in the cytoplasm (Fig 5A).

### SRPK inhibition directly impairs RAN translation from CGG repeats

Next, we asked if SRPK1 inhibition can directly affect RAN translation. We tested this by two means. First, we transfected HEK293T cells with *in vitro* transcribed +1CGG RAN reporter RNA in presence or absence of SRPIN340 (Fig 7D). RNA transfection precludes the requirement of nuclear export of the RNA and reporter RNAs become readily available in the cytoplasm for translation. Interestingly, SRPIN340 treatment led to a significant decrease in RAN translation from +1CGG reporter RNAs, as detected by immunoblot and luciferase assay (Fig 7D and E). We also confirmed direct and selective inhibition of RAN translation by SRPIN340 using an *in vitro* translation system. Addition of 50 μM SRPIN340 to a rabbit reticulocyte lysate (RRL) selectively inhibited translation of *in vitro* transcribed +1CGG reporter RNA without affecting AUG‐nLUC reporter RNA translation (Fig 7F). However, SRPIN340 treatment effects on +1CGG RAN translation were smaller with RNA reporter transfection compared to plasmid reporter transfection (Fig EV4B). Taken together, these results suggest that SRPK1 inhibition suppresses RAN translation through at least two complementary mechanisms: nuclear RNA export and translation efficiency.

### Inhibition of SRPK1 enhances survival of (CGG)_100_ expressing neurons

**Figure 8:**
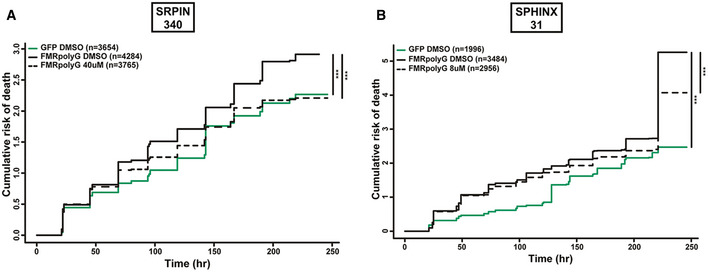
SRPK1 inhibition mitigates (CGG)100 RNA toxicity in primary rat cortical neurons A, BPharmacological targeting of SPRK1 with (A) 40 μM SRPIN340 or (B) 8 μM SPHINX31 reduced the cumulative risk of death in +1(CGG)100‐EGFP (encoding FMRpolyG) expressing neurons. *n* = # of neurons quantified for each condition; Cox proportional hazard analysis, ***P < 0.001.

**Figure EV5:**
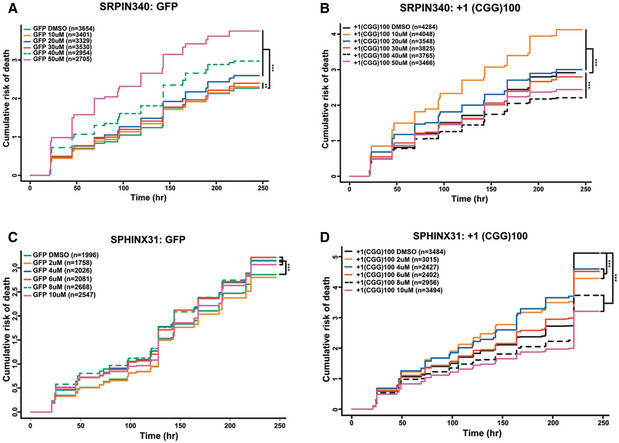
SRPK1 inhibitors improve survival in rodent neurons expressing CGG repeats A–DPharmacological targeting of SRPK1 with range of concentrations of SRPIN340 (A–B) and SPHINX31 (C–D) showing the effects on GFP control (A and C) or +1(CGG)100‐EGFP (encoding FMRpolyG) (B and D) expressing neurons (*n* = # of neurons quantified for each conditions as mentioned within the graphs); Cox proportional hazard analysis, **P < 0.01 and ***P < 0.001.

As pharmacological inhibition of SRPK1 suppressed RAN translation from both +1 FMRpoly(G) and +2 FMRpoly(A) translation frames in mammalian cells and knockdown of SRPK1 in *Drosophila* suppressed CGG repeat toxicity, we asked whether SRPK1 inhibitors could mitigate CGG repeat toxicity in mammalian neurons. We expressed +1(CGG)_100_‐EGFP reporters encoding for +1 FMRpoly(G) in rat primary neurons along with an mApple reporter plasmid that allowed for selective tracking of transfected cells using an automated fluorescence microscopy assay system (Barmada *et al*, 2015; Linsalata *et al*, 2019). An AUG‐driven EGFP reporter served as a control for transfection and exogenous protein expression‐associated toxicity. Consistent with previous results (Linsalata *et al*, 2019), +1(CGG)_100_‐EGFP expression markedly reduced neuronal survival compared to EGFP expression over 10 days (Figs 8A and EV5A). We next treated neurons with SRPIN340 at a range of concentrations from 10 to 50 μM before +1(CGG)_100_‐EGFP reporter transfection (Fig EV5B). Neuronal survival rate was significantly improved with SRPIN340 treatment at concentrations of 30, 40, and 50 μM compared to DMSO treatment. However, SRPIN340 appeared to have some neurotoxicity itself on EGFP‐transfected neurons at higher concentrations, with SRPIN340 treatment at 40 μM showing the most favorable and selective effects on neuronal survival (Figs 8A and EV5A and B).

To confirm that this suppression of neurotoxicity by SRPIN340 treatment is not a drug‐specific effect, we also tested SPHINX31 effects on +1(CGG)_100_‐EGFP‐induced toxicity. Similar to SRPIN340, SPHINX31 significantly improved +1(CGG)_100_‐EGFP expressing neuronal survival at all tested concentrations (Fig EV5C and D). SPHINX31 elicited maximum suppression of neurotoxicity at concentrations of 8 and 10 μM, consistent with our observation of inhibition of RAN translation in a similar range of concentrations. At 8 μM concentration, SPHINX31 showed a promising effect on neuronal survival against +1(CGG)_100_‐EGFP reporter‐induced toxicity over any intrinsic drug‐associated toxicity (Fig 8B). Together, these findings suggest that pharmacological inhibition of SRPK1 can suppress neurotoxicity of expanded CGG repeats through suppression of RAN translation.

## Discussion

Nucleotide repeat expansions as RNA form complex structures that bind and sequester specific RNA binding proteins within different cellular compartments (Krzyzosiak *et al*, 2012; Jazurek *et al*, 2016; Ciesiolka *et al*, 2017). These interactions influence repeat RNA stability, distribution, and translation efficiency, and alter the behavior of the RNA binding proteins, which interact with repeat RNAs. To date, most studies of repeat RNA–protein interactions have relied on *in vitro* capture methods that do not take mRNA cellular context into consideration (Jazurek *et al*, 2016). As such, they have the potential to miss important interacting proteins that might be of lower overall abundance or require interaction within the context of specific cellular compartments or *in vivo* RNA structures. Here, we utilized an alternative method for identifying RNA–RBP interactors that allows for capture of complexes that form within cells under both basal conditions and in response to cellular stress. By applying this tool to CGG repeats that cause FXTAS, we identified a series of novel interactors, including factors with differential interaction profiles after stress induction (Fig 1E–G). Evaluation of these interactors identified SRSF proteins as playing direct roles in CGG repeat toxicity in model systems. Moreover, genetic and pharmacological targeting of the major SRSF kinase (SRPK1) significantly impaired RAN translation at multiple GC‐rich repeat sequences and suppressed toxicity in *Drosophila* and CGG repeat expressing rodent neurons (Figs 5, 6, 7, 8). Taken together, these studies highlight the value of RNA‐tagging for identification of RNA–RBP interactions and suggest that SRPK1 may serve as a therapeutic target worthy of further evaluation in repeat expansion disorders.

Our screening identified multiple SR proteins that interact with CGG repeat RNA and loss of SR protein orthologues in *Drosophila* suppressed CGG repeat toxicity (Figs 1, 2, 3). Though SR proteins play a major role in splicing, they are also implicated in mRNA export, regulation of RNA stability, and translation (Jeong, 2017). SRSF1, 2, and 9 have been previously shown to interact with other repeat RNAs, with functional implications for GGGGCC repeat RNA associated with C9ALS (Sato *et al*, 2009; Donnelly *et al*, 2013; Lee *et al*, 2013; Cooper‐Knock *et al*, 2014). Specifically, sense GGGGCC RNA has been shown to interact with both SRSF1 and SRSF2 and antisense C4G2 RNA has been shown to colocalize with SRSF2 (Cooper‐Knock *et al*, 2015; Hautbergue *et al*, 2017). Moreover, prior data suggested that SRSF1 is important for nuclear export of GGGGCC repeat RNA (Hautbergue *et al*, 2017). Interestingly, analysis of C9ALS patient cerebellum samples has shown extensive alternative splicing (AS) defects in transcripts targeted by hnRNPH1 and SRSF1, indicating that SRSF1 sequestration by GGGGCC RNA may account for this altered splicing (Prudencio *et al*, 2015; Conlon *et al*, 2016). Our data confirm many of these initial observations and extend these results to CGG repeats. Together, they strongly indicate that SR proteins play an important role in repeat RNA toxicity across multiple repeats.

Because of the central importance of SR proteins in many cellular functions, we pivoted to study whether altering the behavior of the major SRSF kinase, SPRK, might serve as a better biological target in repeat expansion disorders. Consistent with this idea, we observed that SRPK inhibition, which influences SRSF1 distribution and behavior, led to a marked nuclear retention of CGG repeat RNA in the nucleus (Fig 7A–C). Thus, at least some of the observed effects of SRPK inhibition and presumably SRSF1 expression manipulation on both CGG repeat distribution and toxicity is mediated by altered repeat RNA export. However, we also observed that SRPK inhibition had a direct effect on RAN translation from CGG repeats, as determined by *in vitro* translation assays and by RNA reporter transfections (Fig 7D–F). SRPK1 may have indirect effects on protein translation through modulation of eIF4E phosphorylation as well as through impacts on AKT signaling (Zhou & Fu, 2013; Brown *et al*, 2014). Alternatively, inhibition of SRPKs can also affect translation through altering the phosphorylation level of eIF4G, as observed earlier for simultaneous knockdown of multiple SRPKs (Hu *et al*, 2012). As such, the exact mechanism(s) by which SRPK inhibition is acting to impair RAN translation and suppress CGG repeat toxicity will require further study.

Protein kinases are attractive targets for drug development. Notably, SPRK1 inhibitors are actively pursued as potential anti‐cancer drugs (van Roosmalen *et al*, 2015; Mavrou & Oltean, 2016; Chandra *et al*, 2020). SRPK1 phosphorylates multiple serine residues in the RS1 domain of SRSF1 to regulate its nuclear localization (Zhou & Fu, 2013). Altered levels of SRSF1 have been reported in many cancers, where phosphorylation of SRSF1 plays a decisive role in alternative splicing of disease‐associated transcripts (Anczuków *et al*, 2015; Sheng *et al*, 2018). Furthermore, in some cancers misregulation of SRPK1 has been linked with cell proliferation, migration, and angiogenesis (van Roosmalen *et al*, 2015). In light of these findings, our discovery of SRPIN340 and SPHINX31 as potential inhibitors of RAN translation is worthy of further pursuit. Furthermore, our mechanistic studies strongly suggest that SRPK1 inhibition may affect RAN translation through multiple pathways (Fig 7). This makes SRPK1 a robust candidate for targeting RAN inhibition that can be applied across multiple repeat expansion disorders. However, there may be some intrinsic toxicity associated with these compounds, given that we observed modest toxicity in neurons expressing GFP alone, especially for SRPIN340 (Figs 8A and EV5). Interestingly, SPHINX31, which has been shown to inhibit SRPK1 more potently than SRPIN340, appeared less neurotoxic (Figs 8A and EV5) (Gammons *et al*, 2013), perhaps due to differential effects of SRPIN340 on SRPK2 (Fukuhara *et al*, 2006). This indicates SPHINX31 to be a more favorable candidate for development in this context.

Besides SRSFs, our dual screens identified a number of other intriguing RNA‐binding proteins that may play a role in CGG repeat‐associated toxicity. For some of these, knockdown mitigates rough eye phenotypes in flies, such as DHX30 and eIF3G, while for others such as hnRNPQ, knockdown enhances rough eye phenotypes in a fashion more consistent with sequestration (Figs 1 and EV2B and C). DHX30 is an ATP‐dependent DEAD/H RNA helicase that has been implicated in translation regulation apoptosis‐associated transcripts (Rizzotto *et al*, 2020). A previous *Drosophila* screen identified the fly homolog of DHX30 as a modest modifier of rough eye phenotype in this FXTAS fly model, which we also observed here (Linsalata *et al*, 2019). Given the roles of other DEAD‐box helicases in RAN translation, including DDX3, the selective identification of this factor as a CGG repeat RNA interactor suggests the need for further study of how it might alter repeat RNA behavior (Linsalata *et al*, 2019). Similarly, the mammalian eIF3 complex protein eIF3F has already been implicated in RAN translation at CAG and GGGGCC repeats (Ayhan *et al*, 2018). While eIF3G is one of the core subunits, eIF3F a non‐core regulatory subunit of the eIF3 complex (Hinnebusch, 2006). Identification of this complex in association with the repeat RNA suggests that it may play a role in its translation, especially given prior studies suggesting eIF3 may be critical in non‐canonical initiation events. Lastly, hnRNPQ is implicated as a key factor in controlling the translation of FMRP from FMR1 transcripts through an IRES‐mediated mechanism (Choi *et al*, 2019). Given recent studies linking CGG repeats, RAN translation, and FMRP synthesis (Rodriguez *et al*, 2020), this factor will require further evaluation in RAN translation assays at this and other repeat structures.

In sum, we developed an in‐cell method for identifying repeat RNA binding proteins and applied it to CGG repeats to reveal both novel interactors and phenotypic modifiers associated with these repeat expansions. Future comparative studies with other repeat elements and in other systems and cellular compartments should allow for a better understanding of repeat RNA–protein complex formation and interactions *in vivo* and guide us in our understanding of the native functions of repeat elements, and how repeats as RNA cause disease and potentially what drug targets are likely to serve as effective therapeutics in these currently untreatable disorders.

## Materials and Methods

### Antibodies

For Western blots, following antibodies were used: FLAG‐M2 at 1:1,000 dilution (mouse, Sigma F1804), 1:2,500 β‐Actin (mouse, Sigma A1978), 1:1,000 SRSF1 (Rabbit, Proteintech 12929‐2‐AP), 1:1,000 SRSF2 (Rabbit, Proteintech 20371‐1‐AP), 1:1,000 Anti‐Phosphoepitope SR proteins, clone 1H4 mouse (MABE50, Millipore Sigma), GAPDH (mouse, Santa Cruz sc‐32233), 1:1,000 eIF2α/EIF2S1 (phospho S51) (rabbit, Abcam ab32157), and 1:1,000 GFP (mouse, Roche/Sigma 11814460001) in 5% non‐fat dry milk. HRP‐conjugated goat‐anti‐mouse (115‐035‐146) or goat‐anti‐rabbit (111‐035‐144) secondary antibodies (Jackson ImmunoResearch Laboratories) were used at a 1:10,000 dilution in 5% non‐fat dry milk.

### Plasmids

NanoLuciferase (nLuc) reporters cloned into pcDNA3.1(+) vector encoding AUG‐nLuc‐3xFLAG, +1CGG100‐nLuc‐3xFLAG (FMRpolyG), +2CGG100‐nLuc‐3xFLAG (FMRpolyA), GGGGCC70‐nLuc‐3xFLAG (GA70) sequences used in this paper are published earlier (Kearse *et al*, 2016; Green *et al*, 2017; Linsalata *et al*, 2019). 2x PP7 stem‐loop sequence as described earlier (Coulon *et al*, 2014; Harlen & Churchman, 2017) was synthesized from GeneWitz. In order to make the RNA‐tagging construct, pcDNA3.1(+) AUG‐nLuc‐3xFLAG and pcDNA3.1(+) +1CGG100‐nLuc‐3xFLAG vectors were modified in two steps. First, nLuc reporter sequence was PCR modified to introduce a stop codon after nLuc sequence and remove the 3xFLAG sequence. This PCR product was cloned back into the original vectors to make pcDNA3.1(+) AUG‐nLuc and pcDNA3.1(+) +1CGG100‐nLuc vectors. Finally, 2× PP7 stem‐loops were cloned into these vectors using ApaI restriction site to finally make—pcDNA3.1(+) AUG‐nLuc‐PP7 and pcDNA3.1(+) +1CGG90‐nLuc‐PP7 constructs. PCP‐NLS sequence was PCR amplified from a PCP containing plasmid to introduce SV40 NLS sequence right after PCP. The PCP sequence‐containing plasmid was generously gifted by Brittany Flores (Flores *et al*, 2019) and originally reported here (Yan *et al*, 2016). Then, a 3xFLAG sequence was amplified from the AUG‐nLuc‐3xFLAG plasmid and PCR sewed with the PCP‐NLS fragment to clone in pcDNA3.1(+) vector using KpnI and ApaI restriction sites to finally make the pcDNA3.1(+) PCP‐NLS‐3xFLAG construct.

See Appendix Table S1 for reporter sequences used in this study.

### Cell culture, drug treatments, and reporter assays

HEK293T and U2OS cells were purchased from American Type Culture Collection (ATCC) and cultured in DMEM media supplemented with 10% FBS. They were confirmed to be mycoplasma free in regular interval.

Luminescence assays were performed as described earlier with slight modifications (Green *et al*, 2017; Linsalata *et al*, 2019). Briefly, HEK293T cells were seeded in 96‐well plates at a concentration of 2 × 10^4^ cells/well and transfected ˜24 h later at ˜ 70% confluency with 25 ng nLuc reporter plasmid and 25 ng pGL4.13 Firefly luciferase reporter plasmid using a ratio of 2:1 jetPRIME (Polyplus) to DNA following manufacturer's recommendation. ˜24 h post‐transfection cells were lysed with 70 μl Glo Lysis buffer (Promega) by incubating for 5 min on a shaker at room temperature. Then, 25 μl of lysate was mixed with NanoGlo substrate diluted 1:50 in NanoGlo buffer (Promega) and 25 μl of lysate was mixed with ONE‐Glo luciferase assay buffer (Promega) in opaque 96‐well plates. Reaction was allowed to continue for 5 min on a shaker in the dark. Finally, luminescence was measured on a GloMax 96 Microplate Luminometer. RNA transfections were performed with *in vitro* transcribed reporter RNAs (nLuc and Firefly luciferase reporters) using *Trans*IT‐mRNA transfection kit (Mirus), per manufacturer’s recommendation. ˜24 h post‐transfection luciferase assays performed as described earlier.

For Western blots, HEK293T cells were seeded in 24‐well plates at a concentration of 1.5 × 10^5^ cells/ml and transfected 24 h later at ˜70% confluency with 250 ng nLuc reporters using a ratio of 2:1 jetPRIME as described earlier. 24‐h post‐transfection cells were lysed in 300 μl of RIPA buffer containing protease inhibitor cocktail (cOmplete™ Mini, Sigma) for 30 min at 4°C with occasional vortexing. Lysates were cleared by centrifugation at 14,000 rpm for 10 min, mixed with 6× SDS sample buffer, and boiled at 90°C for 10 min before running on SDS–PAGE. If required, lysates were stored at −80°C for future experiments. For immunoblot after drug treatment or stress induction, HEK293T cells were seeded in 24‐well plates at 1.5 × 10^5^ cells/ml and treatments were performed as described earlier. For each experimental condition, at least three biological samples were run on 10% SDS–PAGE along with a standard curve for quantification of protein expression. Band intensities were measured using ImageJ and plotted using GraphPad Prism.

For SRPK1 inhibitors, HEK293T cells were plated as described above and pre‐treated with SRPIN340 (Sigma 5042930001) and SPHINX31 (BioVision, B2516‐5) at desired concentrations for 8 and 6 h before the transfection, respectively. Transfections and luminescence assays were performed as described above. For luminescence assays following ISR activation, HEK293T cells were seeded and transfected as described before for 19 h, followed by 5 h of treatment with 2 μM Thapsigargin (Thermo Fisher Scientific). All drugs were dissolved in DMSO and stored as recommended by the manufacturer.

Subcellular fractionation after SRPIN340 treatment was performed as described earlier (Li *et al*, 2015). In brief, HEK293T cells were grown on 6‐well plates. 24 h after SRPIN340 treatment with desired concentration, cells were gently resuspended in ice‐cold 200 μl cytoplasmic extraction buffer (10 mM HEPES pH 7.6, 60 mM KCl, 1 mM EDTA, 0.15% NP‐40, 0.5 mM DTT) supplemented with protease inhibitor and incubated on ice for 5 min. Then, cells were spun at 600 *g* for 4 min at 4°C. The supernatant (cytoplasmic fraction) was then transfer to a new tube. The pellet was then washed twice with the wash buffer (cytoplasmic extraction buffer lacking NP‐40) by spinning at 600 *g* for 2 min each. The pellet was then resuspended in 200 μl nuclear extraction buffer (20 mM HEPES pH 7.9, 1.5 mM MgCl_2_, 430 mM NaCl, 0.2 mM EDTA, 25% glycerol, and protease inhibitor) and rotated on a rotor for 15 min at 4°C. Finally, both cytoplasmic and nuclear fractions were cleared by centrifugation.

### Tagged RNA capture and mass spectrometry

For tagged‐RNA immunoprecipitation (IP), HEK293T cells were grown in SILAC medium for 5–6 passages before transfection. DMEM deficient in L‐arginine and L‐lysine is supplemented with 10% dialyzed FBS and isotopes of lysine (final concentration 146 mg/l) and arginine (final concentration 84 mg/l) for triplex SILAC labeling. For light SILAC: DMEM, L‐lysine (Lys‐0) and L‐arginine (Arg‐0); for medium SILAC: DMEM, L‐lysine‐4,4,5,5‐d4 (Lys‐4) and L‐arginine [13C6] HCl (Arg‐6); while for heavy SILAC: L‐lysine [13C6, 15N2]HCl (Lys‐8) and L‐arginine [13C6, 15N4]HCl (Arg‐10) were added to the medium and filter‐sterilized with a 0.2‐µm filter. For transfection, cells were seeded at 2 × 10^6^ cells/10 cm dish and transfected at ˜70% confluency with 5.25 μg of PP7‐tagged RNA bait plasmids [pcDNA3.1(+) AUG‐nLuc‐PP7, pcDNA3.1(+) +1CGG100‐nLuc‐PP7, and pcDNA3.1(+) (GGGGCC)70‐nLuc‐PP7] along with 0.75 μg of PCP‐NLS‐3xF plasmid, using jetPRIME reagents. Light SILAC was used for AUG‐nLuc‐PP7, medium SILAC +1CGG100‐nLuc‐PP7, and heavy SILAC was used for 1(+) (GGGGCC)70‐nLuc‐PP7 reporter. For ISR activation, cells were seeded similarly and transfected for 19 h, followed by 5 h of treatment with 2 μM Thapsigargin. Three 10‐cm dishes were used per condition. 24 h after transfection, cells were isolated by trypsinization and washing with 1× PBS. Cell pellets were immediately flash‐frozen using liquid nitrogen and proceeded to IP. Cell pellets from three 10 cm plates were pooled together and lysed in 1 ml of NP‐40 buffer (supplemented with cOmplete™ Mini protease inhibitor, 1 mM PMSF, NEB Murine RNase inhibitor, and RNaseIN) by incubating at 4°C for 30 min with occasional pipetting to mix. Lysates were cleared by centrifugation at 20,000 *g* for 10 min, and the supernatant was transferred into a new tube. Protein concentration for each sample was measured by BCA assay (23227, Thermo Fisher Scientific). For IP, 3 mg of total protein was used for each lysate. Lysates were first incubated with 40 μl of pre‐washed protein G beads for 30 min at 4°C to block any non‐specific interaction, then incubated with pre‐washed 40 μl of packed M2 FLAG beads (Sigma) rotating at 4°C for 4 h. Afterward, beads were washed with NP‐40 lysis buffer for a total of four times, 3 min each at 4°C. Before the last wash, 20% of the IP was taken out and saved for Western blot if needed. After the final wash with lysis buffer, beads were transferred to a new tube and finally washed with 1× PBS (mixing by hand) and stored at −80°C until mass spectrometry.

Mass spectrometry was performed by the proteomics resource facility at the Department of Pathology, University of Michigan. In brief, the beads were resuspended in 50 μl of 0.1 M ammonium bicarbonate buffer (pH ˜8). Cysteines were reduced by adding 50 μl of 10 mM DTT and incubating at 45°C for 30 min. An overnight digestion with 1 μg sequencing grade, modified trypsin was carried out at 37°C with constant shaking in a Thermomixer. Samples were completely dried using vacufuge. Resulting peptides were dissolved in 8 μl of 0.1% formic acid/2% acetonitrile solution, and 2 μl of the peptide solution was resolved on a nano‐capillary reverse phase column (Acclaim PepMap C18, 2 micron, 50 cm, Thermo Scientific) using a 0.1% formic acid/2% acetonitrile (Buffer A) and 0.1% formic acid/95% acetonitrile (Buffer B) gradient at 300 nl/min over a period of 180 min (2–22% buffer B in 110 min, 22–40% in 25 min, 40–90% in 5 min followed by holding at 90% buffer B for 5 min and equilibration with Buffer A for 25 min). Eluent was directly introduced into Orbitrap Fusion tribrid mass spectrometer (Thermo Scientific, San Jose CA) using an EasySpray source. Proteins were identified by searching the MS/MS data against *H Sapiens* (UniProt; 20,145 reviewed entries; downloaded on 08‐02‐2017) using Proteome Discoverer (v2.1, Thermo Scientific). False discovery rate (FDR) was determined using Percolator, and proteins/peptides with an FDR of ≤1% were retained for further analysis.

### *Drosophila* lines, rough eye screening, external eye fluorescent measurement, and survival

All fly lines used here and their sources are listed in Appendix Table S2.

To make the dSF2 OE fly line, *Drosophila* dSF2 sequence was PCR amplified (dSF2 F 5’‐CACCATGGGATCACGCAACGAGTGCCG‐3’ and dSF2 R 5’‐ATAGTTAGAACGTGAGCGAGACCTGG‐3’) was cloned into pEntry‐TOPO vector (Thermo Fisher Scientific). The pENTR‐dSF2 vector was recombined with Gateway plasmid pTWH (*Drosophila* Genomics Resource Center, IN). The final vector was used for site‐specific transgenesis using PhiC31 integrase technique (BestGene, CA).

Flies were crossed and raised at 25°C on SY10 food supplemented with dry yeast unless otherwise noted. For rough eye screening, 5–6 virgin female flies expressing GMR‐GAL4‐driven UAS‐*FMR1* (CGG)_90_‐EGFP reporters were crossed with male flies carrying either UAS‐driven siRNA against a candidate gene or a germline mutation. For GGGGCC repeat RNA toxicity modifier phenotyping, a GMR‐GAL4‐driven UAS‐(GGGGCC)_28_‐EGFP reporter containing fly was used. Rough eye phenotypes in F_1_ progenies were scored at 1–2 days post‐eclosion. A minimum of 30 flies (both male and females) from two independent crosses was scored. For rough eye scores were given based on following eye abnormalities: (i) abnormal orientation of the bristles, (ii) supernumerary bristles, (iii) ommatidial fusion and disarray, (iv) presence of necrosis, and (v) collapse/shrinkage of the eye. For each category, three possible scores were given: 1 (for presence of the abnormality), 3 (if the abnormality affected >5% of the total eye), and 5 (if the abnormality affected >50% of the total eye). Eye images were captured using a Leica M125 stereomicroscope and a Leica DFC425 digital camera.

For external eye fluorescent measurement, fly crosses were performed as described above. Fluorescent images were taken at 1–2 days post‐eclosion using a Leica M125 stereomicroscope with GFP filter. All images were taken at the same exposure. GFP images were converted to grayscale, and total intensity was measured using ImageJ.

For survival assays, flies carrying desirable repeat RNA reporter and either a Tub5‐GAL4 GeneSwitch or ElaV‐GAL4 GeneSwitch driver were crossed with a modifier fly. F_1_ progenies were collected 1 day post‐eclosion and placed on SY10 food supplemented with 200 μM RU486 and flipped onto fresh RU486‐containing food every 48 h. For survival, ˜20 flies (equal male and females) from at least three independent crosses maintained at 29°C. Number of deaths recorded every 48 h until expiration and plotted using GraphPad Prism.

### *Drosophila* Western blotting, RNA isolation, and quantitative reverse‐transcription PCR (qRT–PCR)

Immunoblotting and qRT–PCRs were performed as described earlier with slight modifications (Linsalata *et al*, 2019). In brief, 1–2 days post‐eclosion flies carrying (CGG)_90_‐EGFP and a GeneSwitch (Tub5 or ELAV) driver were placed on 200 μM RU486‐supplemented SY10 food with fresh RU486‐supplemented food provided every 24 h, at 29°C. For Western samples, flies carrying (CGG)_90_‐EGFP with ELAV driver were maintained on RU486‐supplemented food for 5 days. Flies were homogenized at 4°C in RIPA buffer supplemented with complete mini protease inhibitor (Roche) and centrifuged at 13,500 *g* for 10 min at 4°C to pellet cuticle and wing debris. The supernatant was removed, mixed with 6× SDS sample buffer, and boiled at 90°C for 10 min before running on SDS–PAGE.

Total RNA for qPCR analysis was isolated as described earlier using TRIzol (Thermo Fisher) (Linsalata *et al*, 2019). For qRT–PCR analysis, 10 μg of total RNA per sample was treated with 2 U of TURBO Dnase (Thermo Fisher) at 37°C for 30 min. DNase‐treated RNA was purified using RNA clean and concentrator‐25 kit (Zymo Research). 500 ng of RNA was used to synthesize cDNA using iScript cDNA synthesis kit (Bio‐Rad). qPCR assays were performed using iQ SYBR Green Supermix (Bio‐Rad) and an iQ5 qPCR system (Bio‐Rad). *Drosophila* α‐Tubulin transcript abundance used for normalization of target transcripts using ∆∆CT method (Livak & Schmittgen, 2001). Primers used for qPCR assays are listed on Appendix Table S3.

### Hybridization Chain Reaction (HCR) and immunocytochemistry (ICC)

HCR v3.0 was performed as previously described (Glineburg *et al*, 2021). Briefly, U2OS cells were seeded at 5 × 10^4^ cells/ml in the chamber and transfected with SRSF1‐FLAG (Addgene #99021) and/or CGG repeat plasmids. Transfection was performed with TransIT‐LT1 Transfection Reagent (Mirus, MIR‐2304) in the ratio of 3:1 of reagent to plasmid. Transfected cells were washed twice with 1xPBS, then fixed in 4% PFA for 10 min at room temperature (RT). After fixation, cells were washed with 1xPBS twice before treating with Turbo DNase for 30 min at 37°C incubator. Then, cells were dehydrated overnight in 70% ethanol at 4°C and rehydrated with 1× PBS for 1 h, prior to immunocytochemistry (ICC). For ICC, cells were permeabilized in 0.1% Triton X‐100 in 1× PBS for 6 min and block with 2% RNAse‐free acetylated BSA in 1× PBS for 20 min at RT. Cells were stained with primary antibody FLAG‐M2 (1:100 dilution, Sigma# F1804) overnight at 4°C, then followed by three times of 5 min 1× PBS washes before staining with secondary antibody (1:500 dilution, Alexa Fluor 488, Invitrogen# A11029) for 1 h at RT in dark. Then, the cells were fixed again with 4% PFA for 10 min at RT followed by washing with 1× PBS 3 times each for 1 min before proceeding to hybridization chain reaction (HCR). HCR v3.0 was performed according to manufacturer’s instructions (Molecular Instrument). The initiator probe for CGG repeats and fluorophore 647‐labeled hairpin probes (B1H1 and B1H2) were synthesized by Molecular Instruments. Repeat RNA were detected by initiator probe CGG at 8 nM and amplified by fluorophore 647 labeled hairpin probes (B1H1 and B1H2) at 60 nM. Finally, cells were stained with DAPI and stored at 4°C in dark until imaging.

Imaging was performed using an oil 60× objective in Olympus FV1000 inverted laser scanning confocal microscope. For all experiments, acquisition parameters were identical between conditions within experiments. Cells were imaged in a series of Z‐planes, and images were analyzed in ImageJ. Average intensity composite images were derived from raw image files. For nuclear and cytoplasmic ratio analysis, signals for each channel were normalized prior to quantification. The background signal was first normalized to non‐transfection group or DMSO control. Next, the ROI was applied to the DAPI channels to specify the region of nucleus along with the 647 channel, which captured CGG RNA amplified by HCR. CGG RNA signal from the nucleus was calculated by the intensity in ROI from DAPI channel in pixels, while the RNA signal from the cytoplasm was calculated by the total intensity from 647 channel subtracted by the RNA intensity in ROI from DAPI channel in pixels. Finally, percentages of CGG RNA intensity in nucleus and cytoplasm were calculated. 20–25 views of images with a number of 85–151 U2OS cells were counted for each condition. *P*‐values were calculated Student’s *t*‐test with Welch’s correction.

### RNA synthesis and rabbit reticulocyte lysate (RRL) *in vitro* translation

For RNA transfections and *in vitro* translation assays using rabbit reticulocyte lysate (RRL), RNAs were *in vitro* transcribed from linearized plasmids containing nanoluciferase (AUG‐nLuc and CGG‐nLuc) and firefly (FLuc) reporters described earlier (Green *et al*, 2017). pcDNA3.1(+) nLuc reporter plasmids were linearized with PspOMI, and pCRII FLuc reporter plasmid was linearized with HindIII‐HF. *In vitro* RNA synthesis was performed using HiScribe T7 ARCA mRNA kit (with tailing; NEB) as per the manufacturer's instructions. Synthesized RNAs were purified using RNA Clean and Concentrator‐25 kits (Zymo Research), and the integrity and size of transcribed RNAs were confirmed by gel electrophoresis before using in transfection assays or in RRL translation reactions.

For *in vitro* translation assays, Flexi Rabbit Reticulocyte Lysate System (Promega) was used as described earlier (Green *et al*, 2017). In brief, 3 nM of in vitro transcribed mRNAs was incubated with 30% RRL, 10 mM amino acid mix minus methionine, 10 mM amino acid mix minus leucine, 0.5 mM MgOAc, 100 mM KCl 0.8 U/µl Murine RNAse Inhibitor (NEB), and indicated amount of SRPIN304 (or DMSO for control) at 30°C for 30 min. Reactions were then diluted 1:7 in Glo Lysis Buffer (Promega) and incubated with NanoGlo Substrate freshly diluted 1:50 in NanoGlo Buffer (Promega). Luminescence was measured using a GloMax 96 Microplate Luminometer.

### Primary rat neuron drug treatment, transfection, and automated fluorescence microscopy imaging

Rat embryonic cortical dissections from E20 Long–Evans rat pups of both sexes were performed as previously described (Malik *et al*, 2018; Flores *et al*, 2019). Dissociated cortical neurons were plated at 0.6 × 10^5^ cells per well on poly‐D‐lysine‐coated 96‐well plate in neuronal growth media (NGM, neurobasal A media, 2% B‐27, 1% Glutamax‐1 (v:v)), and maintained at 37°C for 4 days before transfection. On DIV 4, neurons were treated with SRPIN (10–50 µM) or SPHINX (2–10 µM) or DMSO 8 h before transfection. Neurons were then co‐transfected 0.1 μg of pGW1‐mCherry and either 0.1 μg of pGW1‐GFP or 0.1 μg of pGW +1(CGG)100 GFP DNA per well of a 96‐well culture plate, using Lipofectamine 2000 (Thermo Fisher Scientific).

24 h after transfection, neurons were imaged at 24‐h intervals for 10 days using an automated fluorescence microscopy platform previously described (Arrasate *et al*, 2004; Barmada *et al*, 2015). Images were processed using a custom code written in Python and ImageJ macro language and analyzed by cox proportional hazard test using the survival package in R.

### Prediction of RNA secondary structures

RNA secondary structure prediction was performed using M Fold web server (Zuker, 2003).

### Statistical methods

Statistical analysis was performed using GraphPad Prism 7. For comparison of nLuc reporter luciferase activity, Western blots, and HCR/ICC analysis, two‐tailed Student’s *t*‐tests were performed with Welch’s correction. For all experiments, specification of statistical analysis and sample numbers (*n*) is provided with figure legends. A minimum of three independent biological samples (*n* > 3) with technical replication of results from each sample. To avoid the effects of subjective bias, fly screening was done by at least two independent investigators using multiple crosses. Eye necrosis/width measurements were done by blind data analysis.

## Author contributions

IM and PKT conceptualized the experiments. Indranil performed the formal analysis for most of the described assays and was responsible for developing the methodology along with PKT. Y‐JT performed and analyzed the experiments relevant to HCR and RNA–protein co‐localization. SEW performed the studies and analyzed the experiments relevant to neuronal survival. PR, KZ, and KMG performed drosophila experiments and assisted with design and analysis of these studies. IM wrote the initial draft of the manuscript along with PKT, and all authors reviewed and edited the manuscript. IM, SEW, KMG, and PKT were responsible for obtaining funding for the work. PKT oversaw the project.

## Conflict of interest

The authors declare that they have no conflict of interest.

## Supporting information



Appendix

Expanded View Figures PDF

Dataset EV1

Source Data for Figure 1

Source Data for Figure 2

Source Data for Figure 3

Source Data for Figure 4

Source Data for Figure 5

Source Data for Figure 6

Source Data for Figure 7

## Data Availability

Raw mass spectrometry data are provided in Data Set EV1. Mass spectrometry data are also deposited to the ProteomeXchange Consortium via the PRIDE partner repository with the dataset identifier PXD027000. The datasets produced in this study are available in the following databases: ProteomeXchange (http://proteomecentral.proteomexchange.org/cgi/GetDataset) using the data set identifier (PXD027000).
