# Supplementary material for: SRSF protein kinase 1 modulates RAN translation and suppresses CGG repeat toxicity
Source: EMBO Mol Med. 2021 Sep 20;13(11):e14163. doi: 10.15252/emmm.202114163 (PMC8573603; doi:10.15252/emmm.202114163)
Supplement: Supplementary file 1 — Appendix [file EMMM-13-e14163-s004.pdf]

Appendix

Table of contents:

Appendix Figure S1. Predicted RNA secondary structure of *FMR1* 5’-CGG90
Appendix Table S1. Sequence maps of reporters used in this study
Appendix Table S2. List of flies used in this study
Appendix Table S3. List of primers used for *Drosophila* RT-qPCR
References for Primers

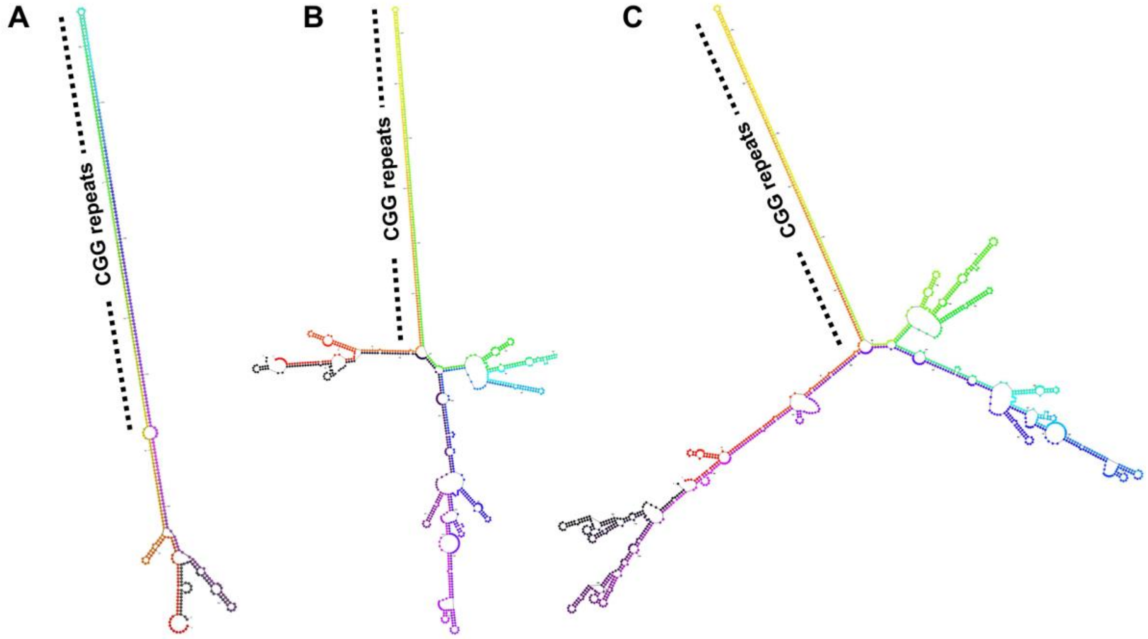

Appendix Figure S1. Predicted RNA secondary structure of *FMR1* 5’-CGG90 (A), *FMR1* 5’-CGG90-nLuc (B) and *FMR1* 5’-CGG90-nLuc-PP7 showing CGG repeat RNA secondary structure (hairpin) remains unperturbed by surrounding sequences. RNA secondary structures were predicted using M fold.

Appendix Table S1
Sequence maps of reporters used in this study

AUG-nLuc-PP7: T7-ATGnLuc-2xPP7

GAAATtaatacgactcactatagggAGACCCAAGCTGGCTAGCGTTTAAACTTAAGCTTGGCAATCC
GGTACTGTTGGTAAAGCCACC**ATG**gtcttcacactcgaagatttcgttggggactggcgacagacagccggctaca
acctggaccaagtccttgaacagggaggtgtgtccagtttgtttcagaatctcggggtgtccgtaactccgatccaaaggattgtcctg
agcggtgaaaatgggctgaagatcgacatccatgtcatcatcccgtatgaaggtctgagcggcgaccaaatgggccagatcgaaa
aaatttttaaggtggtgtaccctgtggatgatcatcactttaaggtgatcctgcactatggcacactggtaatcgacggggttacgccga
acatgatcgactatttcggacggccgtatgaaggcatcgccgtgttcgacggcaaaaagatcactgtaacagggaccctgtggaac
ggcaacaaaattatcgacgagcgcctgatcaaccccgacggctccctgctgttccgagtaaccatcaacggagtgaccggctggc
ggctgtgcgaacgcattctggcgTAAGGCCGCGACTCTAGAGggcccAGATTCACCGGTTAAGGTACC
TAATTGCCTAGAAAGGAGCAGACGATATGGCGTCGCTCCCTGCAGGTCGACTCTAGAAAC
CAGCAGAGCATATGGGCTCGCTGGCTGCAGTATTCCCGGGTTCATTTAAGGTACCTAATT
GCCTAGAAAGGAGCAGACGATATGGCGTCGCTCCCTGCAGGTCGACTCTAGAAACCAGC
AGAGCATATGGGCTCGCTGGCTGCAGTATTCCCGGGTTCATTaccggt

CGG-nLuc-PP7: T7-FMR1 5'-(CGG)90-GGGnLuc-2xPP7

GAAATtaatacgactcactatagggAGACCCAAGCTGGCTAGCGTTTAAACTTAAGCTTGGTACCGA
GCTCGGATCCACTAGTCCAGTGTGGTGGAATTCGTTAACAGATCTGCTCAGCTCCGTTTC
GGTTTCacttccggtggagggccgcctctgagcgggcggcgggccgacggcgagcgcgggcggcggcggtgacggaggc
gccgctgccagggggcgtgcggcagcg**(CGG)90**CGCTGGGCCTCGAGGATATCAAGATCTGGCCTCG
GCGGCCAAGCTTGGCAATCCGGTACTGTTGGTAAAGCCACCC**GGG**gtcttcacactcgaagatttcg
ttggggactggcgacagacagccggctacaacctggaccaagtccttgaacagggaggtgtgtccagtttgtttcagaatctcgggg
tgtccgtaactccgatccaaaggattgtcctgagcggtgaaaatgggctgaagatcgacatccatgtcatcatcccgtatgaaggtct
gagcggcgaccaaatgggccagatcgaaaaaatttttaaggtggtgtaccctgtggatgatcatcactttaaggtgatcctgcactat
ggcacactggtaatcgacggggttacgccgaacatgatcgactatttcggacggccgtatgaaggcatcgccgtgttcgacggcaa
aaagatcactgtaacagggaccctgtggaacggcaacaaaattatcgacgagcgcctgatcaaccccgacggctccctgctgttc
cgagtaaccatcaacggagtgaccggctggcggctgtgcgaacgcattctggcgTAAGGCCGCGACTCTAGAGggc
ccAGATTCACCGGTTAAGGTACCTAATTGCCTAGAAAGGAGCAGACGATATGGCGTCGCT
CCCTGCAGGTCGACTCTAGAAACCAGCAGAGCATATGGGCTCGCTGGCTGCAGTATTCC
CGGGTTCATTTAAGGTACCTAATTGCCTAGAAAGGAGCAGACGATATGGCGTCGCTCCCT
GCAGGTCGACTCTAGAAACCAGCAGAGCATATGGGCTCGCTGGCTGCAGTATTCCCGGG
TTCATTaccggt

PCP-NLS-3xFLAG: T7-ATGPCP-NLS-3xFLAG

GAAATaatacgactcactatagggAGACCCAAGCTGGCTAGCGTTTAAACTTAAGCTTGgtacCTCTC
GAGAATTCTCACGCGCCGGATCCGCCACC**ATG**TCCAAAACCATCGTTCTTTCGGTCGGCG
AGGCTACTCGCACTCTGACTGAGATCCAGTCCACCGCAGACCGTCAGATCTTCGAAGAGA
AGGTCGGGCCTCTGGTGGGTCGGCTGCGCCTCACGGCTTCGCTCCGTCAAAACGGAGC
CAAGACCGCGTATCGCGTCAACCTAAAACTGGATCAGGCGGACGTCGTTGATTGCTCCAC
CAGCGTCTGCGGCGAGCTTCCGAAAGTGCGCTACACTCAGGTATGGTCGCACGACGTGA
CAATCGTTGCGAATAGCACCGAGGCCTCGCGCAAATCGTTGTACGATTTGACCAAGTCCC
TCGTCGCGACCTCGCAGGTCGAAGATCTTGTCGTCAACCTTGTGCCGCTGGGCCGTCGT
GCGGACCCGCTAGCCTCCTGCGGCCGCCCAAAAAAGAagagaaaggtagaagacccccGACTACA
AAGACCATGACGGTGATTATAAAGATCATGACATCGATTACAAGGATGACGATGACAAGta
a

PP7-nLUC 5'-PP7 (control for IP/Western)

agattcaccggttaaggtaccTAATTGCCTAGAAAGGAGCAGACGATATGGCGTCGCTCCCTGCAG
GTCGACTCTAGAAACCAGCAGAGCATATGGGCTCGCTGGCTGCAGTATTCCCGGGTTCAT
TTAAGGTACCTAATTGCCTAGAAAGGAGCAGACGATATGGCGTCGCTCCCTGCAGGTCGA

CTCTAGAAACCAGCAGAGCATATGGGCTCGCTGGCTGCAGTATTCCCGGGTTCATTaccggt
agattccggccgggggtcttcacactcgaagatttcgttggggactggcgacagacagccggctacaacctggaccaagtccttga
acagggaggtgtgtccagtttgtttcagaatctcggggtgtccgtaactccgatccaaaggattgtcctgagcggtgaaaatgggctg
aagatcgacatccatgtcatcatcccgtatgaaggtctgagcggcgacggcgcgccagattcaccggttaaggtaccTAATTG
CCTAGAAAGGAGCAGACGATATGGCGTCGCTCCCTGCAGGTCGACTCTAGAAACCAGCA
GAGCATATGGGCTCGCTGGCTGCAGTATTCCCGGGTTCATTTAAGGTACCTAATTGCCTA
GAAAGGAGCAGACGATATGGCGTCGCTCCCTGCAGGTCGACTCTAGAAACCAGCAGAGC
ATATGGGCTCGCTGGCTGCAGTATTCCCGGGTTCATTaccggt

Appendix Table S2
List of flies used in this study

Modifier/target gene	Drosophila homolog	Source/stock number
HNRNPH	Glo	BDSC 33668
PABPN1	Pabp2	BDSC 34602
NCBP1	Cbp80	VDRC 110673
EIF3G	eIF3G1	BDSC 43243
RPLP0	RpLP0	BDSC 31370
LARP1 #1	larp	BDSC 11687
LARP1 #2	larp	BDSC 42578
DHX30	bgcn	VDRC 108334
DDX39B #1	Hel25E	BDSC 11043
DDX39B #2	Hel25E	BDSC 33666
SYNCRIP #1	Syp	BDSC 55577
SYNCRIP #2	Syp	BDSC 56972
SRSF1 #1	SF2	BDSC 32367
SRSF1 #2	SF2	BDSC 29522
SRSF2 #1	SC35	BDSC 65888
SRSF2 #2	SC35	BDSC 20169
SRSF 6 #1	B52	BDSC 10265
SRSF 6 #2	B52	BDSC 37519
SRPK1 #1	SRPK	BDSC 57295
SRPK1 #2	SRPK	BDSC 57587
Non-targeting Control	mCherry RNAi	BDSC 35785
Non-targeting Control	LUC RNAi	BDSC 31603/Todd lab (G4C2/LUC)
Non-targeting Control	lexA RNAI	BDSC 67947/Todd lab (G4C2/lexA)
SRSF1 overexpression	dSF2	Todd lab

Appendix Table S3

List of primers used for *Drosophila* RT-qPCR

Primer	Target	Sequence	Reference
SRSF1 F	SRSF1/ dSF2	5'-TACCGCGTCATGGTTACTGG-3'	(Hautbergue *et al*, 2017)
SRSF1 R	SRSF1/ dSF2	5'-GTACGCGAATGTAGGCAACC-3'	(Hautbergue *et al*, 2017)
SRPK1 F	SRPK1	5'-TGGGCCCGTCTGGACCACAA-3'	(Sopko *et al*, 2014)
SRPK1 R	SRPK1	5'-TCGCCGTCACCGGAGTCCAT-3'	(Sopko *et al*, 2014)
dTUB84b F	α-Tubulin	5'-ATCCGCTGACTGAGGGCACTG-3'	(Hautbergue *et al*, 2017)
dTUB84b R	α-Tubulin	5'-GTAGAGTTTTCCAGTTGTGGCG-3'	(Hautbergue *et al*, 2017)

Reference:

Hautbergue GM, Castelli LM, Ferraiuolo L, Sanchez-Martinez A, Cooper-Knock J, Higginbottom A, Lin Y-H, Bauer CS, Dodd JE, Myszczynska MA, *et al* (2017) SRSF1-dependent nuclear export inhibition of C9ORF72 repeat transcripts prevents neurodegeneration and associated motor deficits. *Nat Commun* 8

Sopko R, Foos M, Vinayagam A, Zhai B, Binari R, Hu Y, Randklev S, Perkins LA, Gygi SP & Perrimon N (2014) Combining genetic perturbations and proteomics to examine kinase-phosphatase networks in Drosophila embryos. *Dev Cell* 31: 114–127
