# Supplementary material for: SRSF protein kinase 1 modulates RAN translation and suppresses CGG repeat toxicity
Source: EMBO Mol Med. 2021 Sep 20;13(11):e14163. doi: 10.15252/emmm.202114163 (PMC8573603; doi:10.15252/emmm.202114163)
Supplement: Supplementary file 2 — Expanded View Figures PDF [file EMMM-13-e14163-s008.pdf]

Expanded View Figures

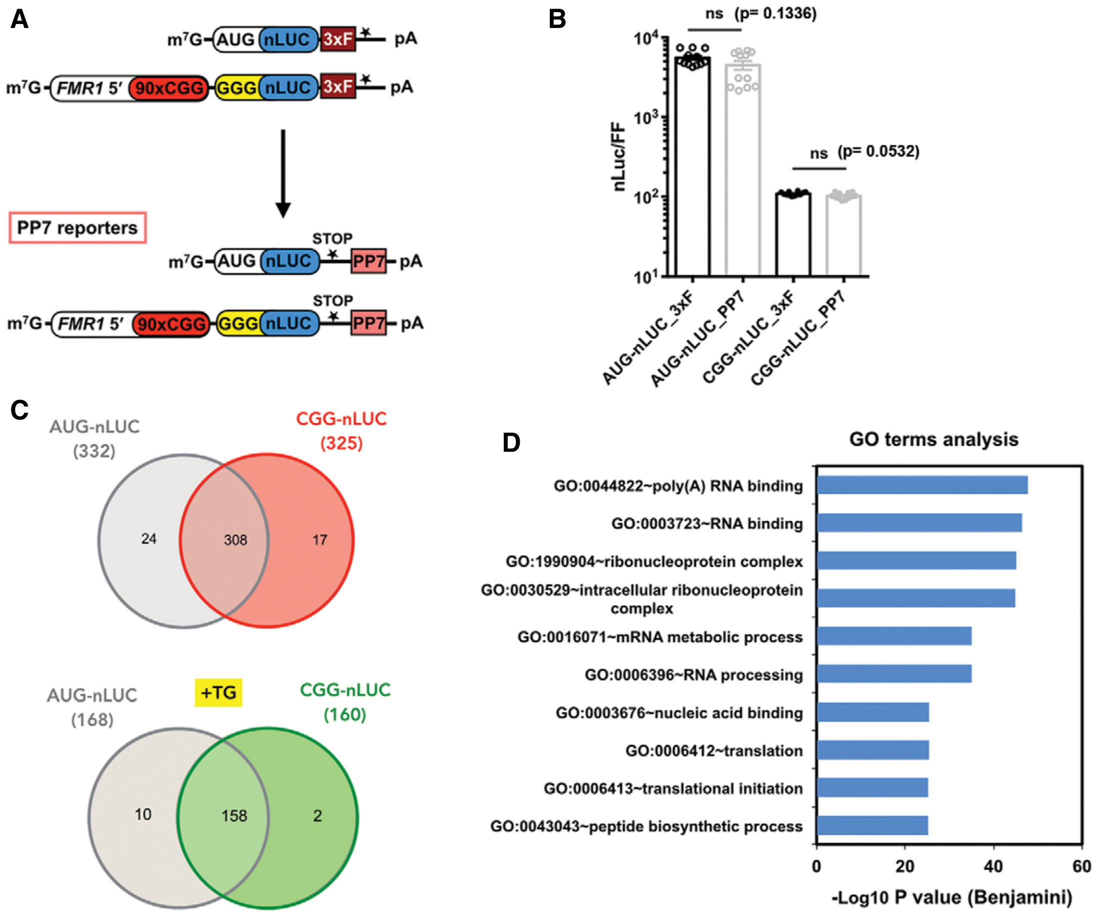

Figure EV1. Reporters used for RNA-tagging experiments and proteins identified by mass spectrometry.

A Schematic of PP7-tagged RNA reporters used in this study and the previously published 3x FLAG (3xF)-tagged reporters that were used to develop the PP7 reporters.

B Relative nanoluciferase (nLuc) expression from PP7-tagged reporters compared to the primary 3x FLAG (3xF)-tagged reporters showed no significant differences in translational efficiency in HEK293T cells (n = 12 biological replicates). Two-tailed Student's t-test with Welch's correction is used for statistical analysis.

C Venn diagrams indicate total number of proteins identified for AUG-nLuc and CGG-nLuc RNA-tagging reporters without or with TG treatment (TG^+).

D GO term analysis of manually curated differentially enriched CGG-interacting proteins.

Source data are available online for this figure.

© 2021 The Authors

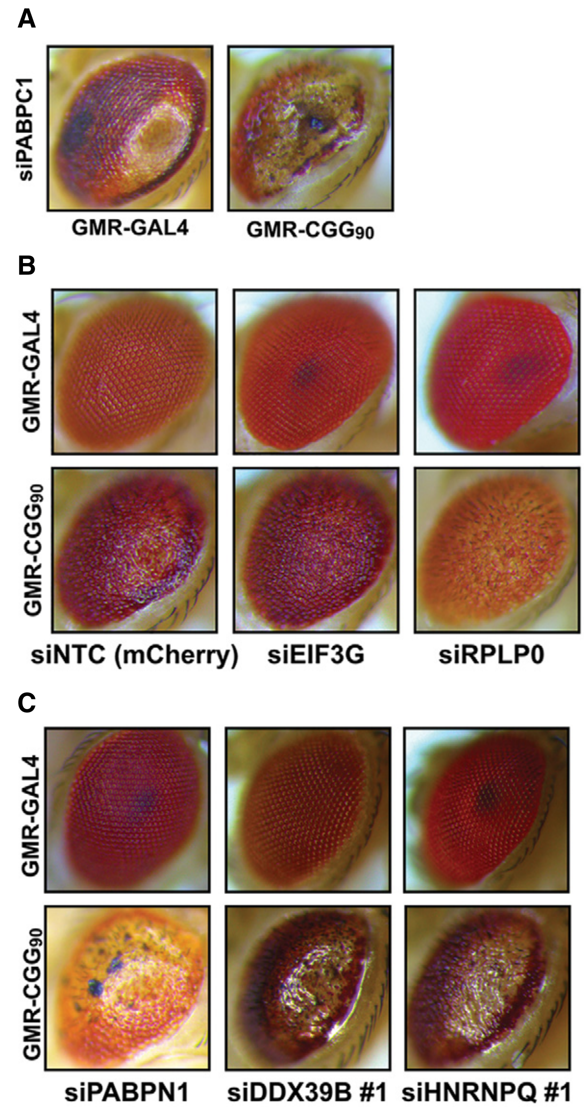

Figure EV2. Modifiers of CGG repeat-associated toxicity in *Drosophila*.

A Representative photographs of fly eyes expressing either GMR-GAL4 driver alone or the (CGG)90-EGFP construct under a GMR-GAL4 driver, with siRNA against fly homologs of PABPC1.

B, C Representative examples of (B) suppressors (eIF3G, RPLP0, DHX30, and SRSF4/6) and (C) enhancers (PABPN1, DDX39B, and hnRNPQ/Syncrip) of CGG repeat toxicity.

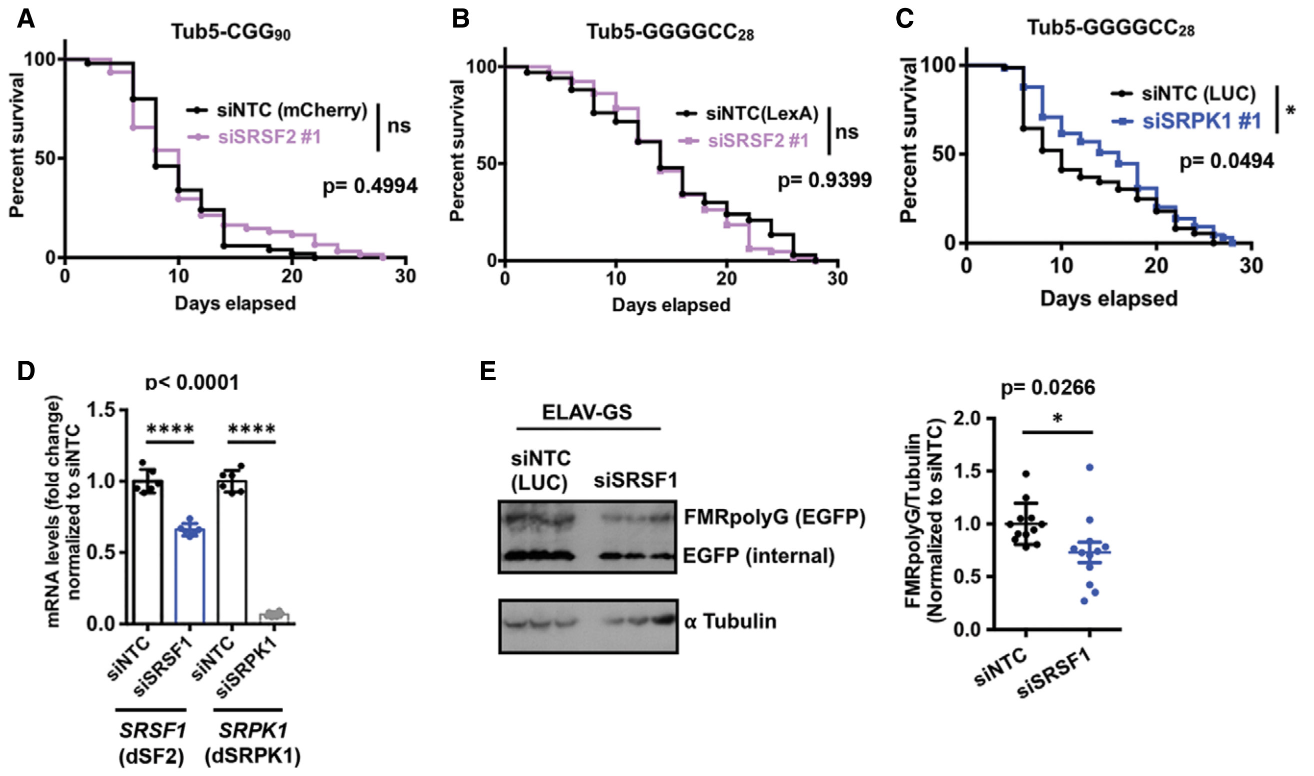

Figure EV3. Impact of SRSF and SRPK modulation on RAN translation and survival assays in *Drosophila*.

A–C Survival assays of (CGG)90-EGFP and (G4C2)28-EGFP expressing fly under Tub5-GS driver with respective siRNAs as mentioned. Log-rank Mantel–Cox test; n = 50–61 (A), 65–67 (B); and 65–73 (C). *P < 0.05.

D *Drosophila* SRSF1 (dSF2, blue bar) and SRPK1 (gray) levels after siRNA knockdown as compared to non-targeting siRNA (siNTC, black bars), quantified by qRT–PCR. Error bars represent mean ± SD RNAs from two (n = 2 biological repeats) independent fly crosses (20–25 flies/genotype per cross) used to run RT–qPCR in triplicates (3 technical replicates). All data points presented in the graphs.

E Western blot of FMR-polyG-EGFP RAN products in (CGG)90-EGFP/ELAV-GS flies with or without SRSF1 knockdown. Error bars represent mean ± SD. Total protein from three (n = 3 biological repeats) independent fly crosses (20–22 flies/genotype per cross) and run in four replicates per gel (technical replicates). All data points presented in the graphs.

Data information: Statistical analysis in (D) and (E) is performed using Student's t-test with Welch's correction. *P < 0.05, ****P < 0.0001.

 © 2021 The Authors

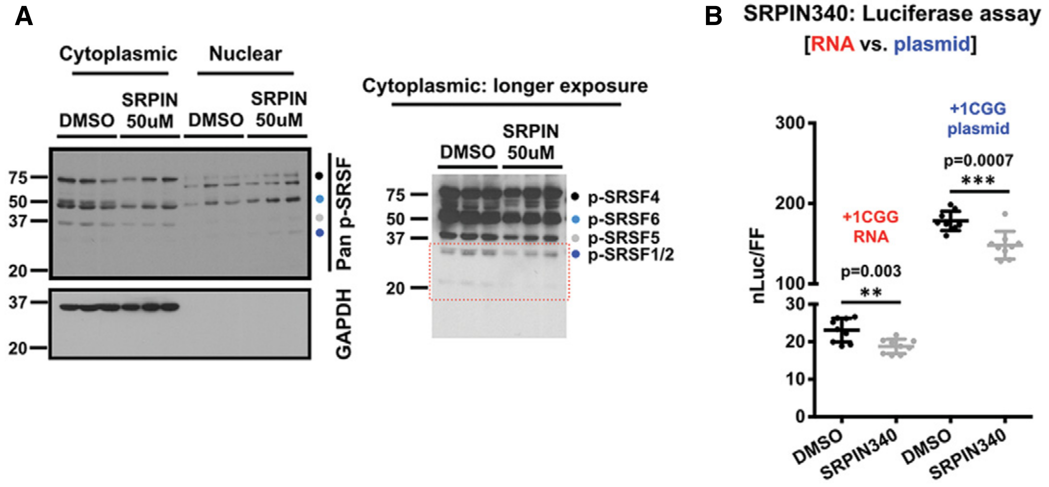

Figure EV4. SRPK1 inhibition alters SR protein phosphorylation and inhibits RAN translation.

A Subcellular levels of phosphorylated SRSF proteins with or without SRPIN340 treatment measured by Western blot using an antibody, which detects phosphorylated SR proteins (p-SRSFs). GAPDH is used as cytoplasmic marker and loading control for cytoplasmic fraction of phosphorylated SRSFs (n = 3 biological repeats). Detected p-SRSFs are labeled/color-coded. p-SRSF1/2 data presented in Fig 7A are outlined in red dots.

B Relative nanoluciferase (nLuc) expression of +1 CGG-nLuc-3xF reporter transfected as plasmid or as *in vitro* transcribed RNA in presence or absence of SRPIN340 treatment (n = 9). Error bars represent mean ± SD (n = 12 biological repeats). Statistical analysis is performed using two-tailed Student's t-test with Welch's correction. $^{**}P < 0.01$, $^{***}P < 0.001$.

© 2021 The Authors

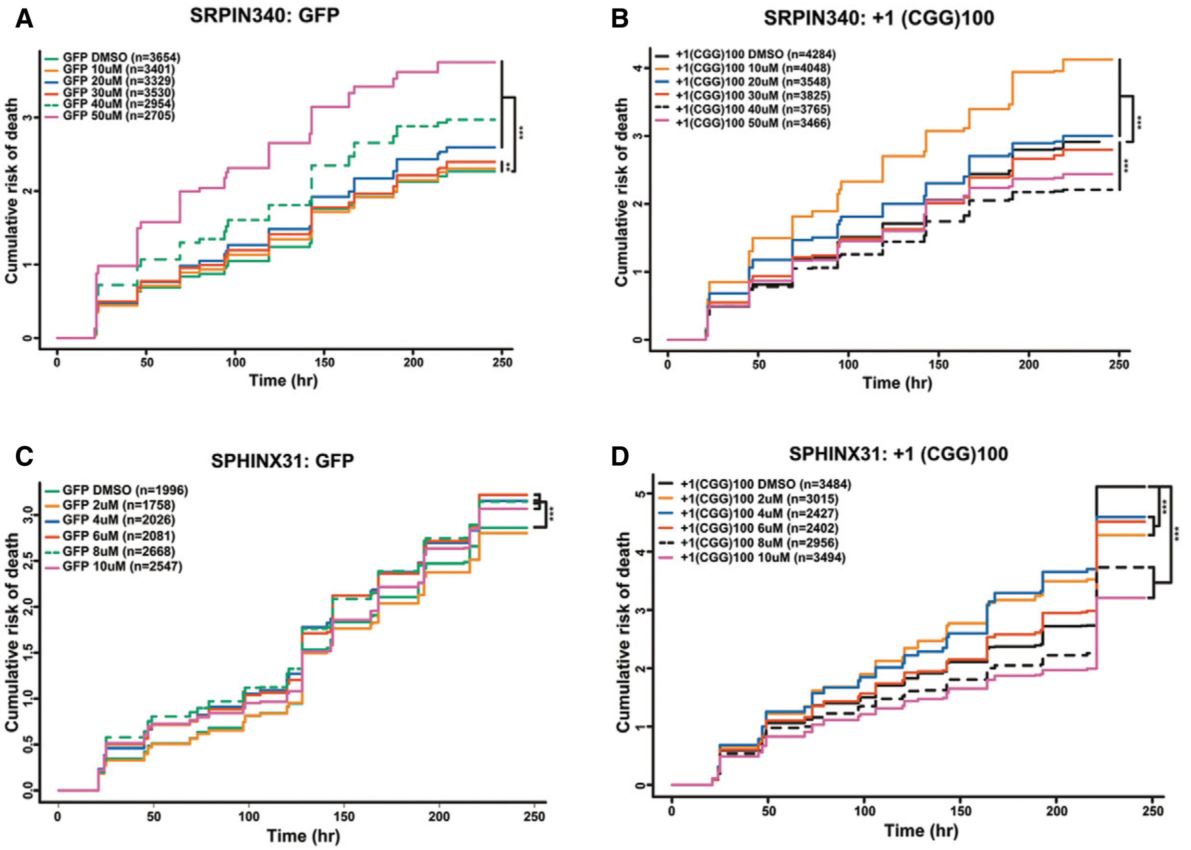

Figure EV5. SRPK1 inhibitors improve survival in rodent neurons expressing CGG repeats.

A–D Pharmacological targeting of SRPK1 with range of concentrations of SRPIN340 (A–B) and SPHINX31 (C–D) showing the effects on GFP control (A and C) or +1(CGG) 100-EGFP (encoding FMRpolyG) (B and D) expressing neurons (*n* = # of neurons quantified for each conditions as mentioned within the graphs); Cox proportional hazard analysis, $^{**}P < 0.01$ and $^{***}P < 0.001$.

 © 2021 The Authors
