# Supplementary figures and images for: SRSF protein kinase 1 modulates RAN translation and suppresses CGG repeat toxicity
Source: EMBO Mol Med. 2021 Sep 20;13(11):e14163. doi: 10.15252/emmm.202114163 (PMC8573603; doi:10.15252/emmm.202114163)

**Figure 2A**

**DAPI**

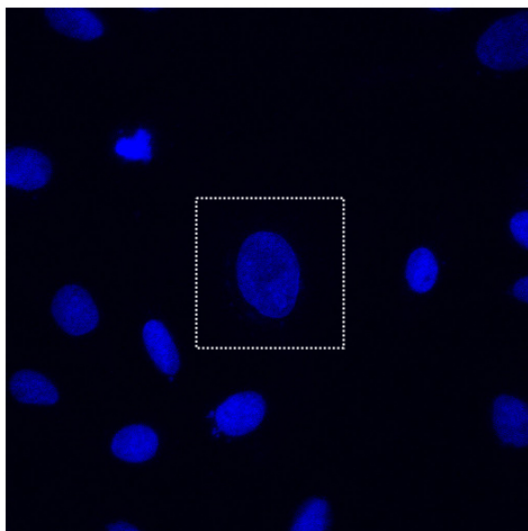

**Figure 2A**

**CGG RNA**

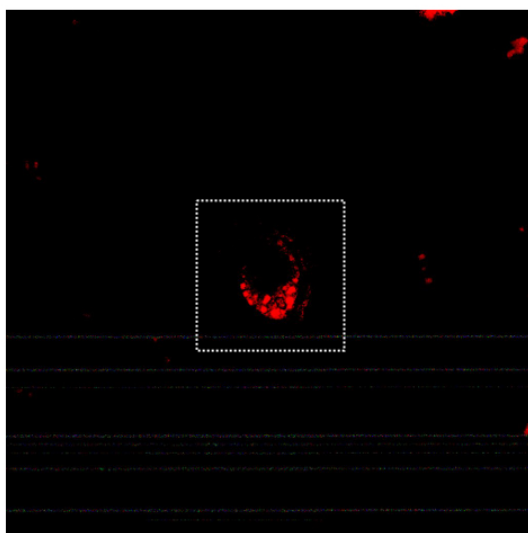

**Figure 2A**

**SRSF1-FLAG**

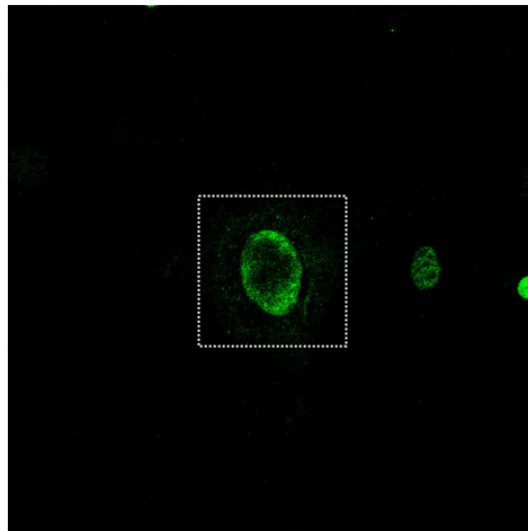

**Figure 2A**

**Merge**

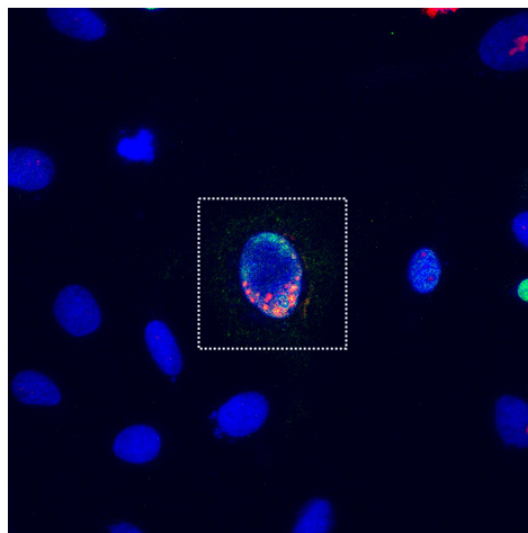

Figure 2C

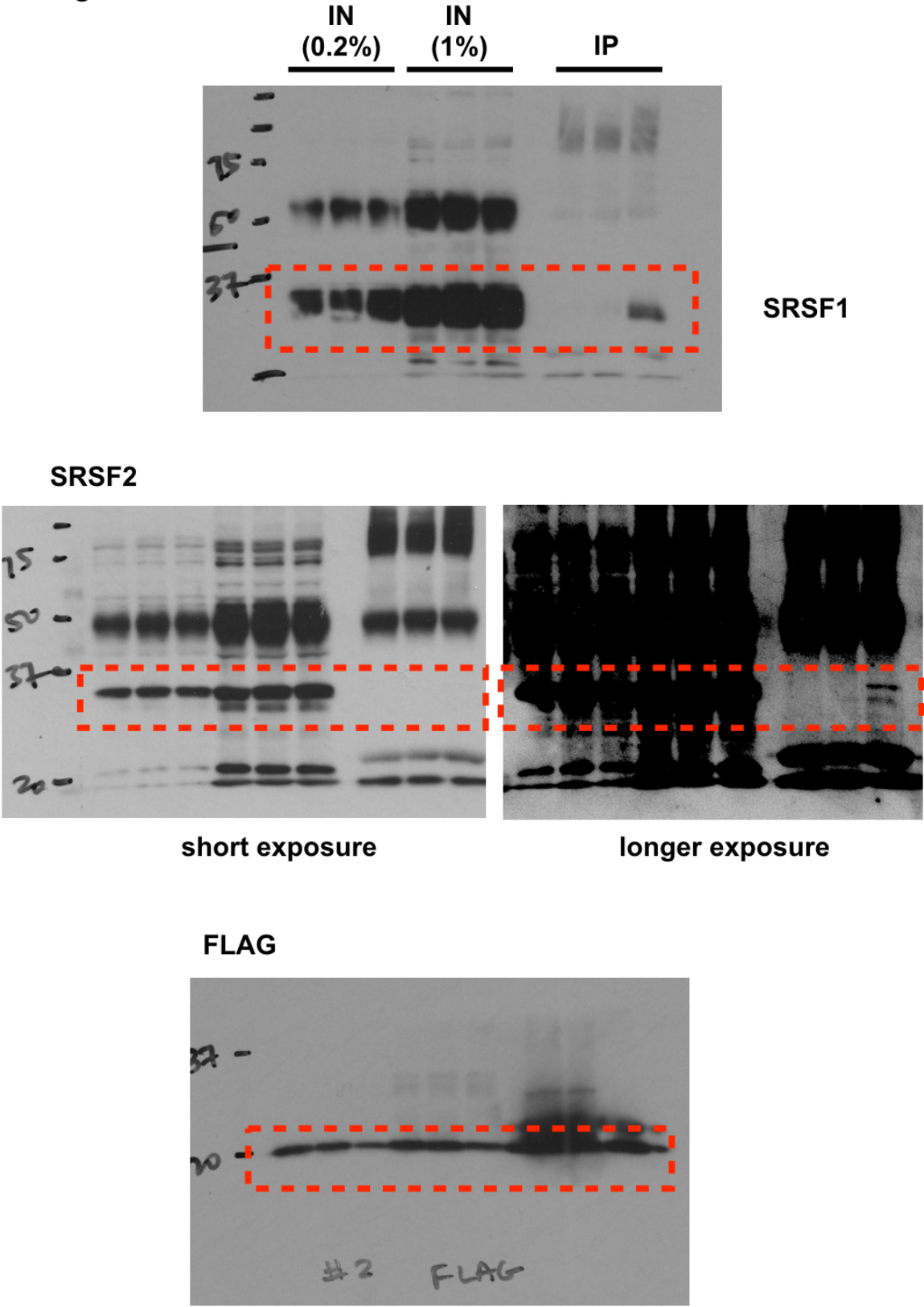

Supplement: Supplementary file 5 — Source Data for Figure 2 [file EMMM-13-e14163-s001.pdf]

Figure  
5B

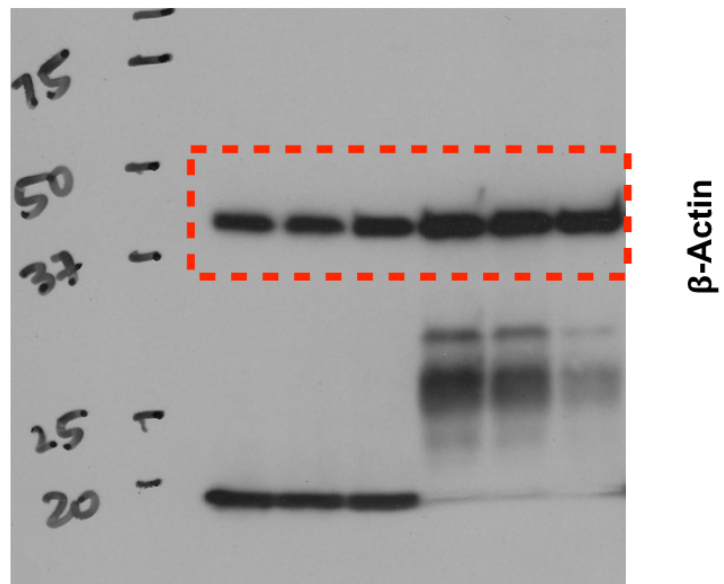

Figure  
5B

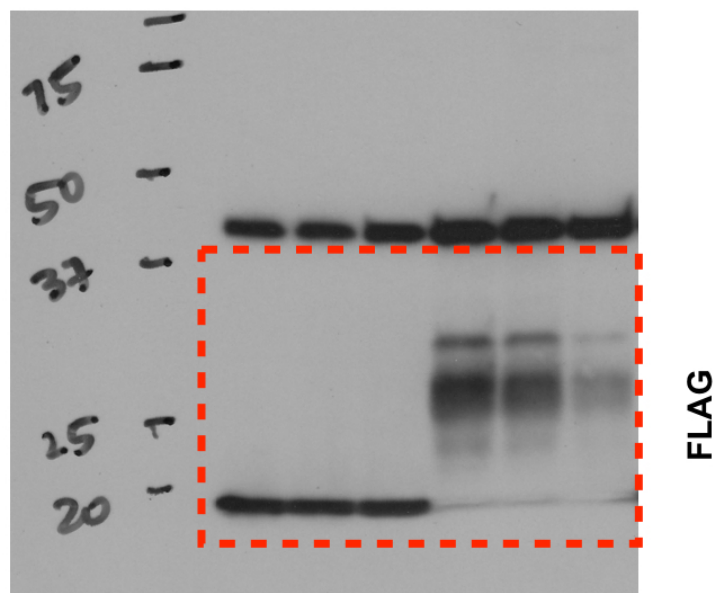

**Figure  
5D**

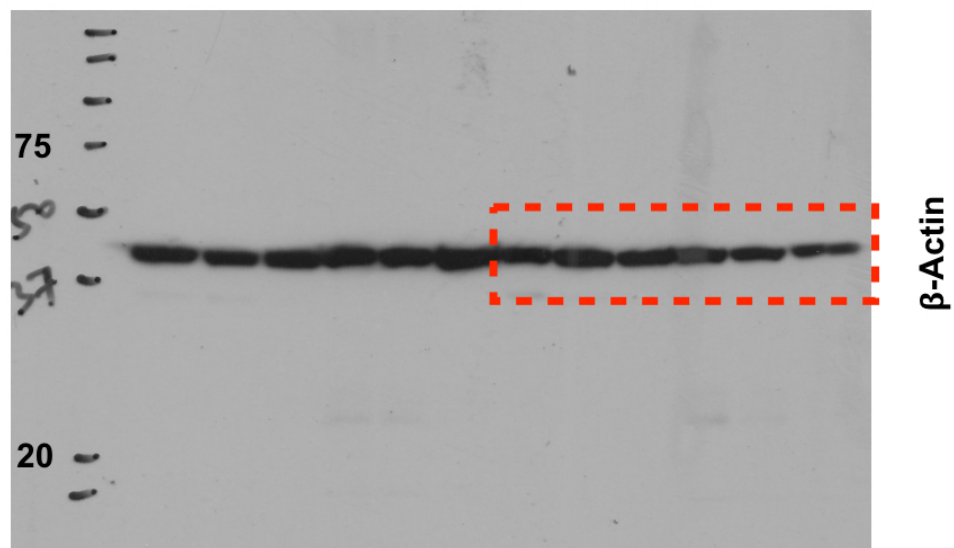

**Figure  
5D**

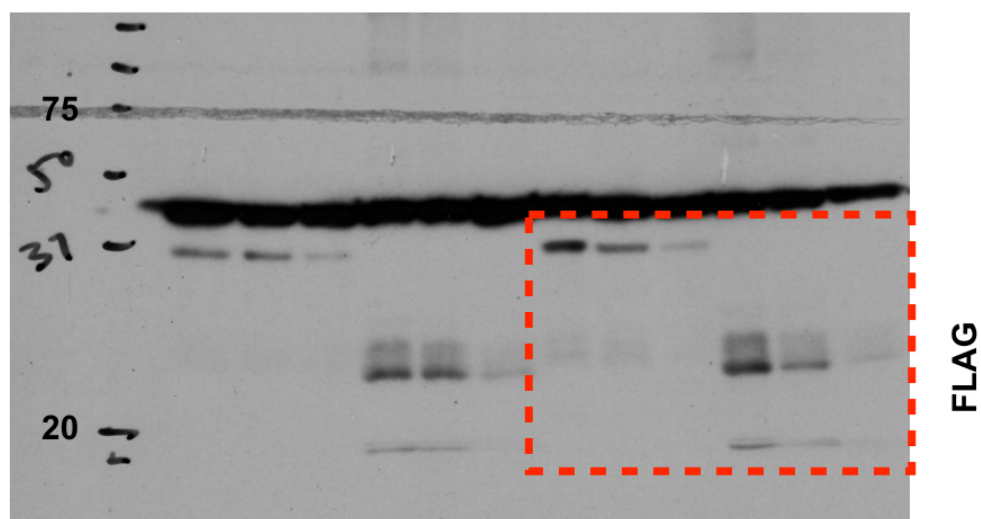

Figure 5E

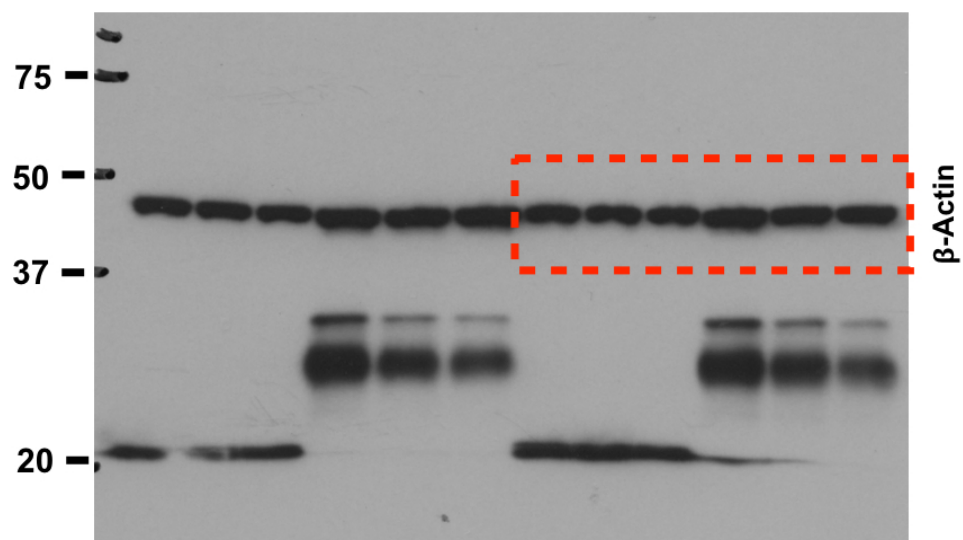

Figure 5E

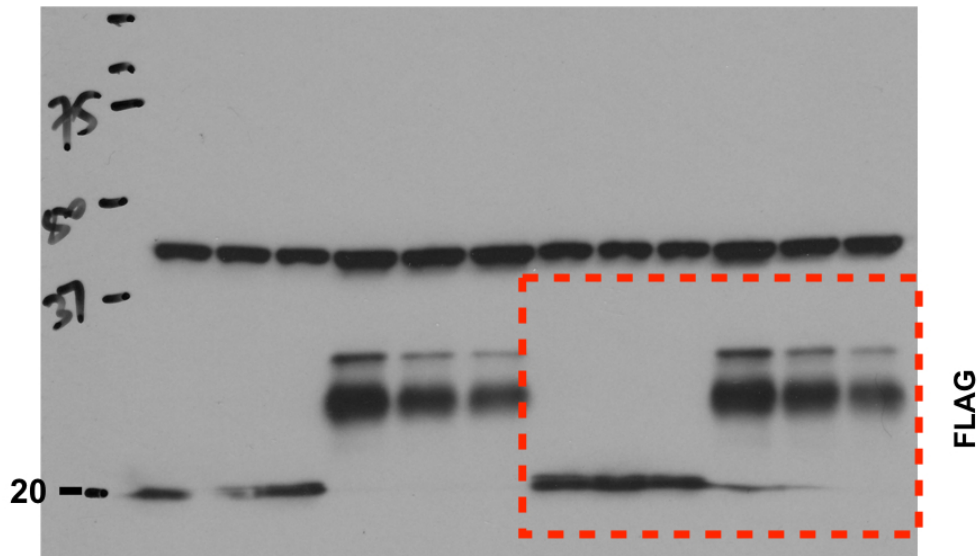

Figure 5F

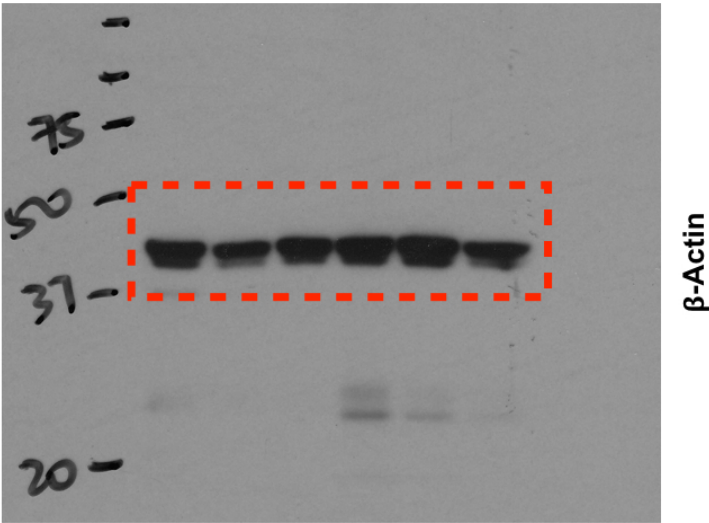

Figure 5F

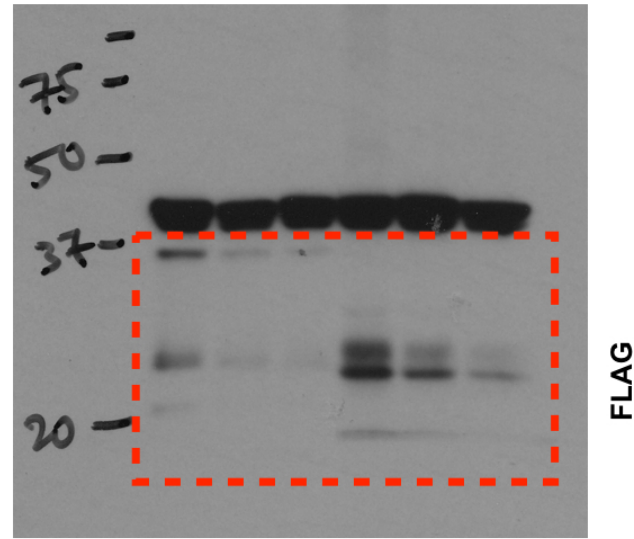

Supplement: Supplementary file 8 — Source Data for Figure 5 [file EMMM-13-e14163-s006.zip › Figure 5 Source data/Figure 5_Raw source data.pdf]

Figure  
6A

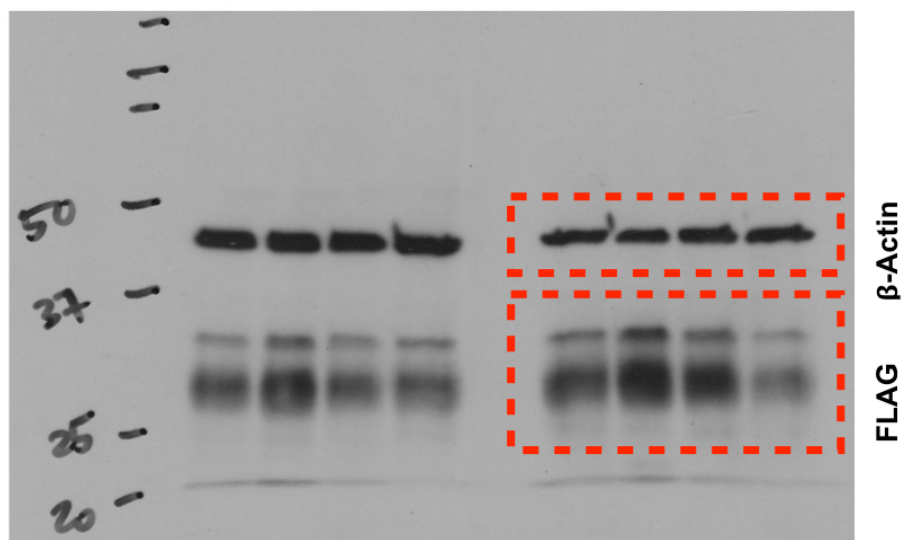

Figure  
6C

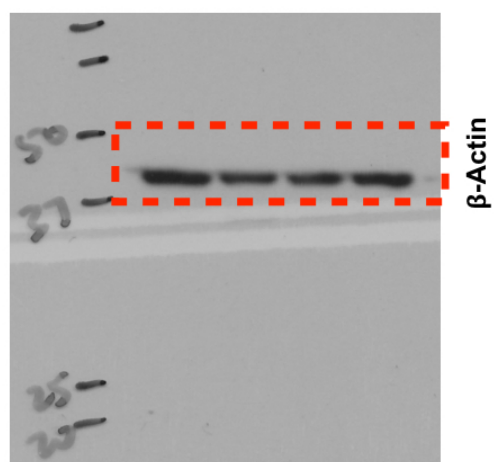

Figure  
6C

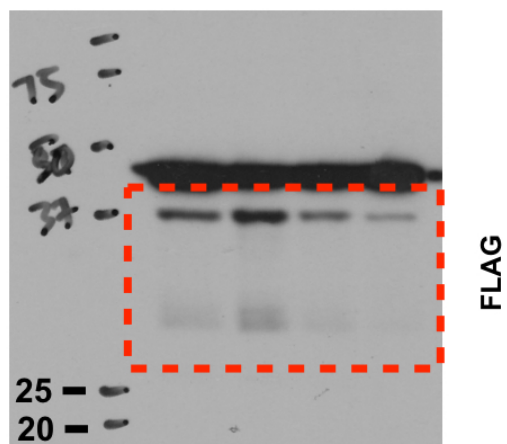

Figure 6D

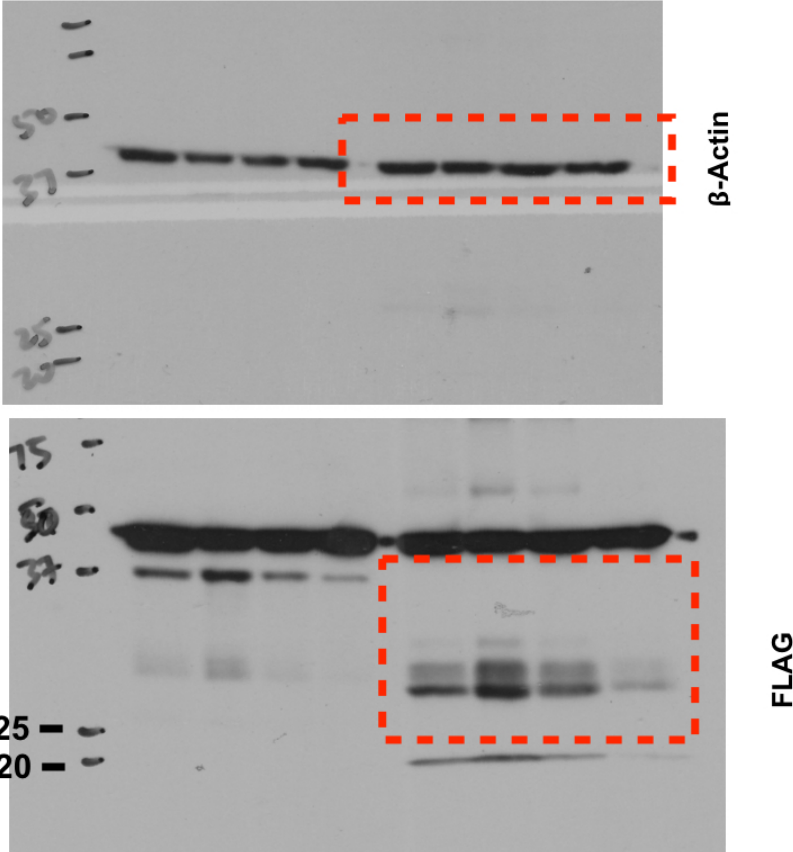

Figure 6E

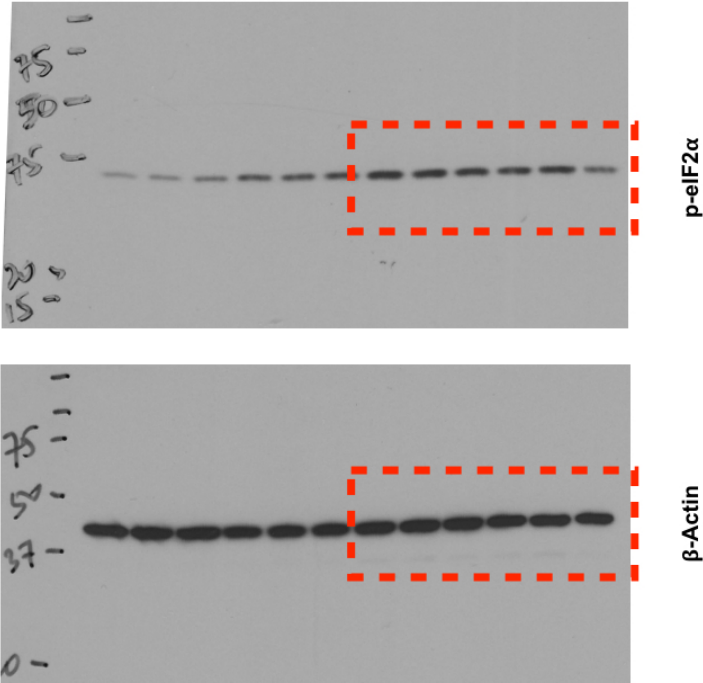

Figure 6F

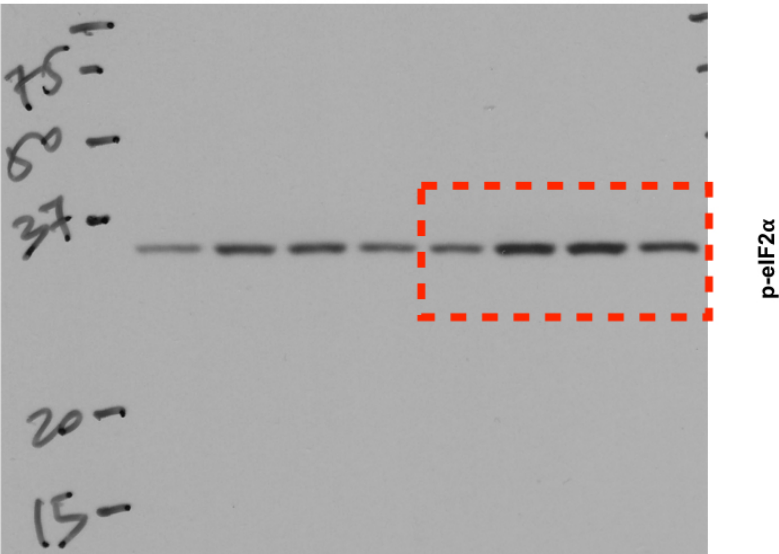

Figure 6F

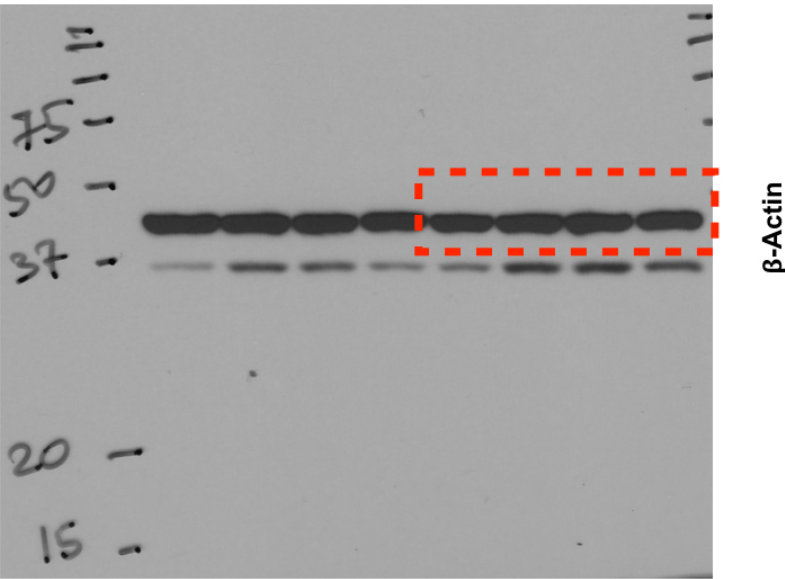

Supplement: Supplementary file 9 — Source Data for Figure 6 [file EMMM-13-e14163-s010.zip › Figure 6 Source data/Figure 6_Raw source data.pdf]
