# Supplementary material for: SRSF protein kinase 1 modulates RAN translation and suppresses CGG repeat toxicity
Source: EMBO Mol Med. 2021 Sep 20;13(11):e14163. doi: 10.15252/emmm.202114163 (PMC8573603; doi:10.15252/emmm.202114163)

Figure 7A

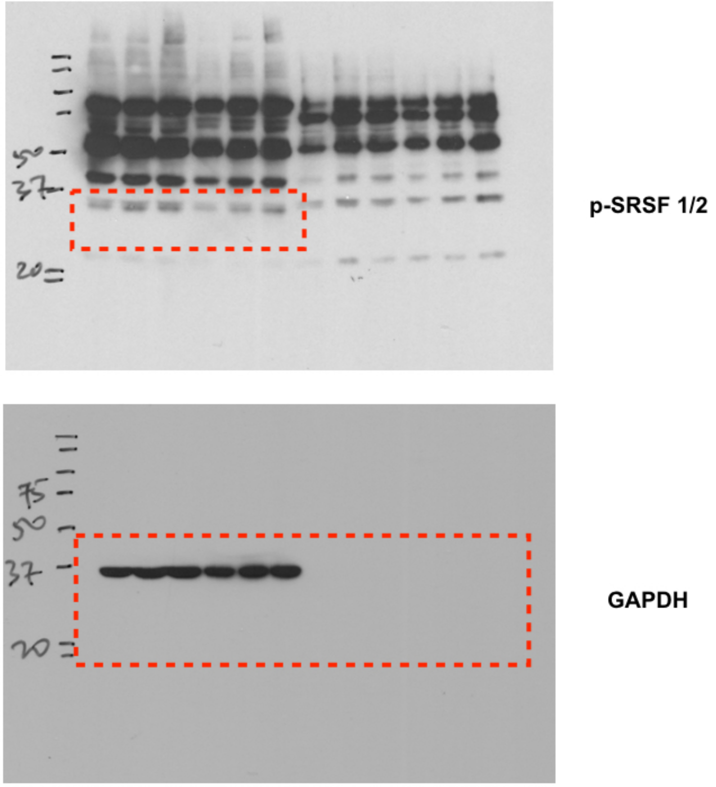

Figure 7B: DMSO panel

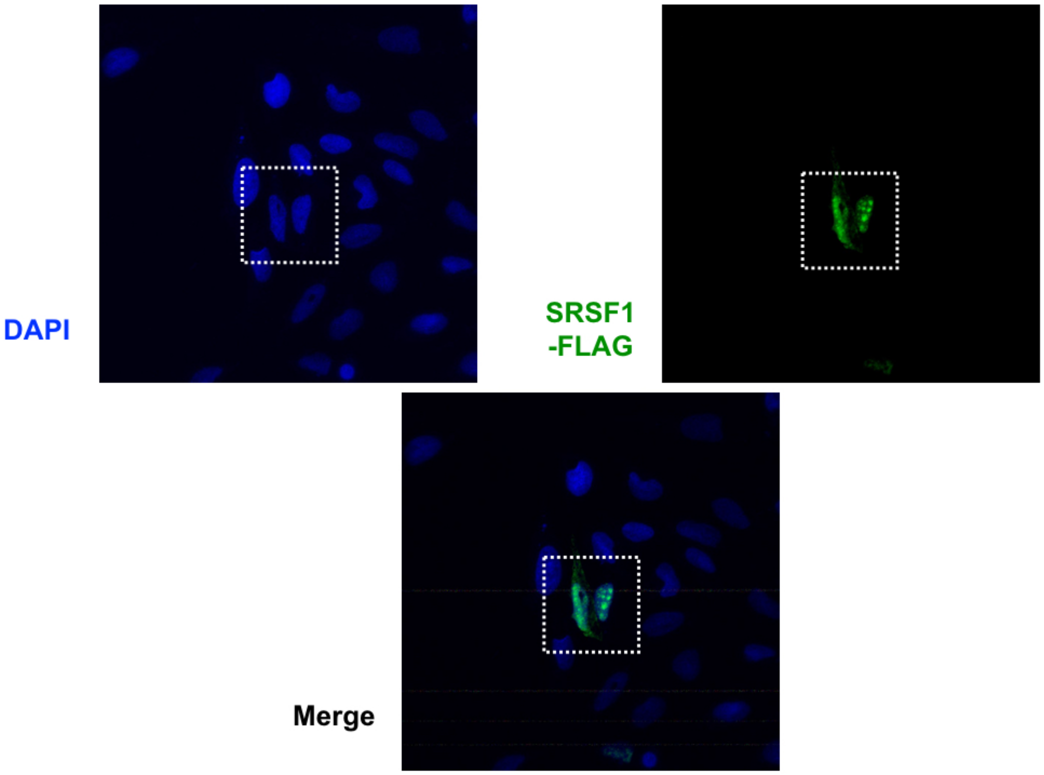

Figure 7B: SRPIN panel

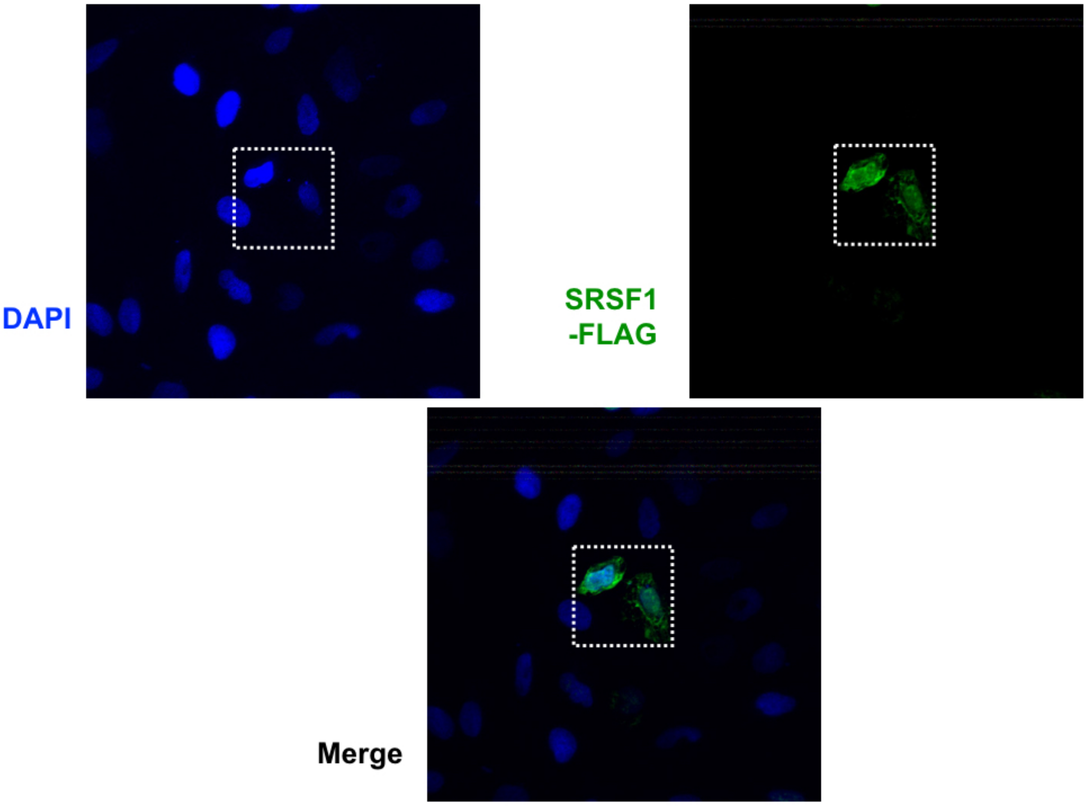

Figure 7C: DMSO panel

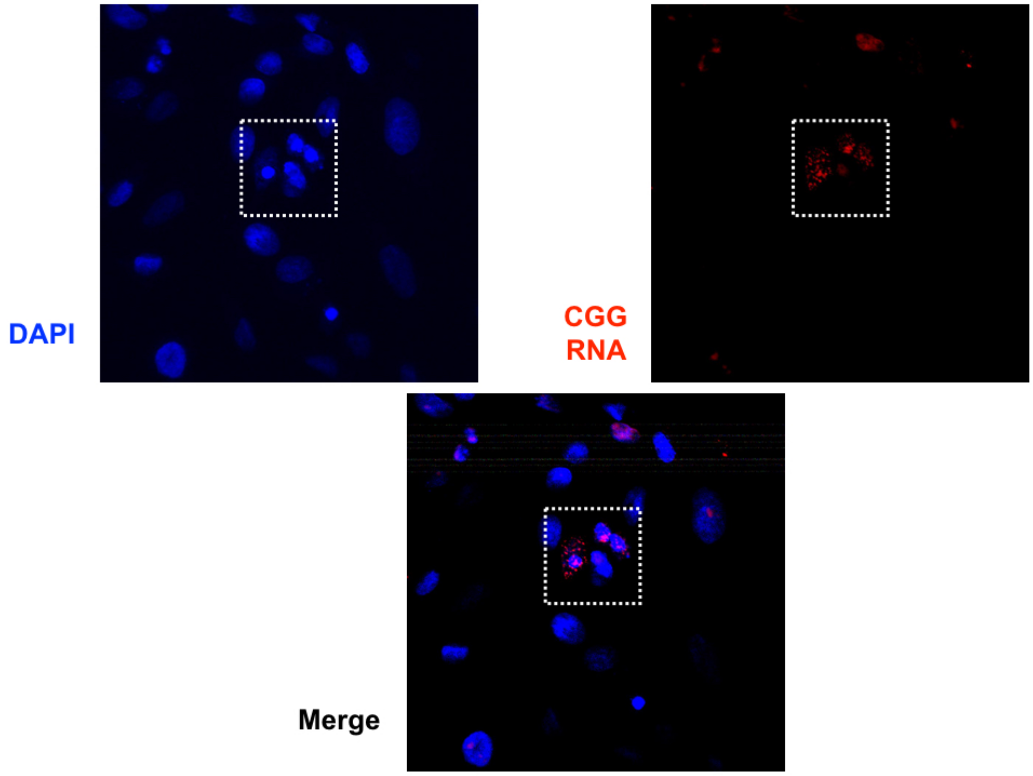

Figure 7C: SRPIN panel

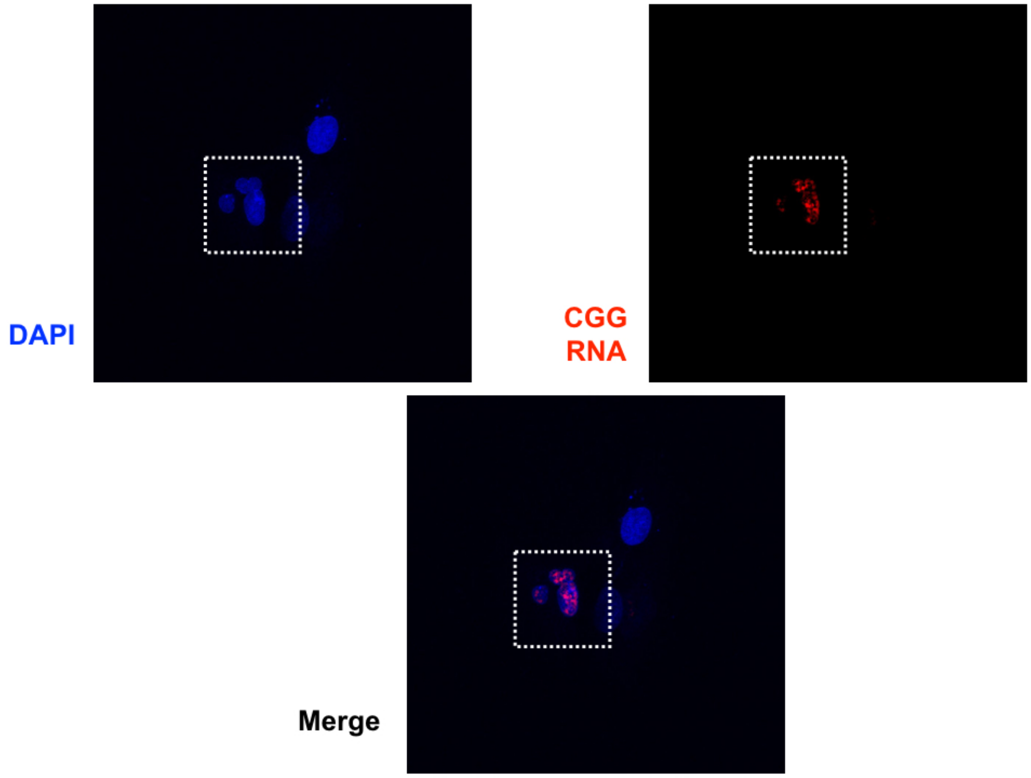

Figure 7D

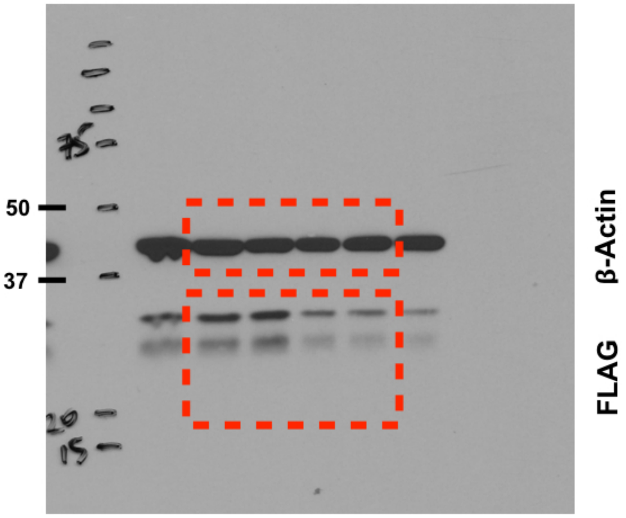

Supplement: Supplementary file 10 — Source Data for Figure 7 [file EMMM-13-e14163-s003.zip › Figure 7 Source data/Figure 7_Raw source data.pdf]
