# Supplementary material for: SRSF protein kinase 1 modulates RAN translation and suppresses CGG repeat toxicity
Source: EMBO Mol Med. 2021 Sep 20;13(11):e14163. doi: 10.15252/emmm.202114163 (PMC8573603; doi:10.15252/emmm.202114163)
Supplement: Supplementary file 11 [file EMMM-13-e14163-s007.pdf]

EMBO Molecular Medicine

SRSF protein kinase 1 modulates RAN translation and suppresses CGG repeat toxicity

Indranil Malik, Yi-Ju Tseng, Shannon Wright, Prithika Ramaiyer, Kristina Zheng, Katelyn Green, and Peter Todd
DOI: 10.15252/emmm.202114163

Corresponding author: Peter Todd (petertod@med.umich.edu)

Review Timeline:

Submission Date:	21st Feb 21
Editorial Decision:	17th Mar 21
Revision Received:	22nd Jul 21
Editorial Decision:	20th Aug 21
Revision Received:	28th Aug 21
Accepted:	30th Aug 21

Editor: Zeljko Durdevic

Transaction Report:

(Note: With the exception of the correction of typographical or spelling errors that could be a source of ambiguity, letters and reports are not edited. Depending on transfer agreements, referee reports obtained elsewhere may or may not be included in this compilation. Referee reports are anonymous unless the Referee chooses to sign their reports.)

17th Mar 2021

Dear Dr. Todd,

Thank you for the submission of your manuscript to EMBO Molecular Medicine. We have now heard back from the three referees who agreed to evaluate your manuscript. As you will see from the reports below, the referees acknowledge the interest of the study. However, they raise serious and partially overlapping concerns that should be addressed in a major revision of the present manuscript.

Overall it is clear that publication of the manuscript cannot be considered at this stage. I also note that addressing the reviewers concerns in full, except of the point 1 from referee #2 that should be addressed using RNA folding prediction algorithm, will be necessary for further considering the manuscript in our journal and this appears to require a lot of additional work and experimentation. I am unsure whether you will be able or willing to address those and return a revised manuscript within the three to six months deadline. On the other hand, given the potential interest of the findings, I would be willing to consider a revised manuscript with the understanding that the referee concerns must be fully addressed and that acceptance of the manuscript would entail a second round of review.

Please note that EMBO Molecular Medicine encourages a single round of revision only and therefore, acceptance or rejection of the manuscript will depend on the completeness of your responses included in the next, final version of the manuscript. For this reason, and to save you from any frustrations in the end, I would strongly advise against returning an incomplete revision and would also understand your decision if you chose to rather seek rapid publication elsewhere at this stage.

Should you find that the requested revisions are not feasible within the constraints outlined here and choose, therefore, to submit your paper elsewhere, we would welcome a message to this effect.

Yours sincerely,

Zeljko Durdevic

Zeljko Durdevic
Editor
EMBO Molecular Medicine

***** Reviewer's comments *****

Referee #1 (Comments on Novelty/Model System for Author):

Experiments performed in a rigorous manner using appropriate model systems.

Referee #1 (Remarks for Author):

The manuscript by Malik investigated the important role of RBPs in modulating RAN translation and repeat RNA pathology. In recent years, RAN translation of repeat RNA has been discovered in multiple neurodegenerative diseases, including FXTAS and ALS/FTD, which may play a critical role in disease pathogenesis. To elucidate the mechanisms underlying RAN translation in vivo, Malik and colleagues employed a new repeat RNA-tagging system to capture repeat associated RBPs under normal and stress conditions, by which they identified multiple SR proteins interacting with CGG repeat RNA. Using Drosophila models, they showed that inhibiting SR proteins and their modulator SRPK suppresses repeat-induced toxicity in eye morphology and survival. They further used cell models to indicate that pharmacological inhibition of SRPK1 suppresses repeat toxicity through RAN translation. Overall, the findings provide novel insight into RAN translation and repeat RNA pathology in FXTAS.

The experiments are well designed and the manuscript is well written. I have some comments and suggestions for the authors to consider.

Major comments
1. Context-specific interactions between repeat RNA and RBPs is quite interesting. Please provide an excel spreadsheet of the complete data? Supplemental figure 1 seems important (more than the GO analysis in Figure 1G), this might be better in the main figure.
2. Drosophila genetic experiments are mostly based on RNAi knockdown. The authors should provide information on knockdown efficiency of each RNAi line (e.g., if this information exists from previous publications).
3. In Drosophila genetic experiments, the authors mainly investigated eye morphology and survival, both of which are not true neurodegenerative phenotypes. It might be helpful to include Figure S3A in the main figure, which provides direct evidence indicating a neuronal interaction. Additionally, does the rough eye phenotype recapitulate in the ELAV-GS flies with SRSF1 and SRPK1 knock-down?
4. It wasn't discussed clearly how SR proteins modulate RAN translation of repeat RNA. Do SR proteins sequester repeat RNA in the nucleus? How do they recruit other components of the RAN translation machinery? Can the authors speculate the potential underlying mechanisms and include a few more sentences in the discussion section?
5. Does altering SRSF phosphorylation change localization of repeat RNA constructs? Does stress change the subcellular localization of these RNA transcripts? Does this contribute to the translational changes you see? RNA imaging experiments can be done with the PCP/PP7 constructs.
6. Does knockdown of SRPK1 change RAN reporter levels in flies or cells?
7. Does altering SRSF phosphorylation with these drugs impact the ISR?

Minor comments
1. Please add description of genetic background being appropriately controlled in this manuscript.

This is critical especially when quantitative assays are performed. Please indicate what controls are used and whether different strains are backcrossed to equilibrate the genetic background.
2. Although some terms are widely used in the field, it is helpful to list their full names, such as ISR.
3. Please italicize Drosophila.
4. Figure 4A is a confusing schematic because these constructs aren't spliced, so why mention splicing?
5. Explain more clearly why SRSF6 is enriched in mass spec but doesn't validate but immunoblot.

Referee #2 (Remarks for Author):

The manuscript from Malik et al., entitled "SRSF protein kinase 1 modulates RAN translation and suppresses CGG repeat toxicity" describes a robust quantitative analysis of CGG repeat RNA protein interactors, the identification of SRSF proteins as contributors to toxicity using fly models of CGG- and G4C2- repeat toxicity, and modest therapeutic effects of knockdown and inhibition of the SRSF-regulating protein kinase SRPK. The results are interesting but require a bit more mechanistic connection in order to be convincing. This point, along with additional points are listed below for consideration.

1. The structure of the CGG repeat-containing RNA is likely key to the selectivity of the interacting proteins identified. Have the authors validated that the structure of the 90xCGG mRNA is the same when it is fused to GGG-nLuc and PP7 hairpins? At a minimum, it would seem that comparison of the PP7-fusion to unfused RANT reporter (and AUG reporter) would be need to establish lack of effect of the added sequence.
2. It is unclear if, prior to IP of SILAC-labeled protein lysates, the lysates of the different conditions were mixed in order to carry out a single pull-down. This is the central advantage of SILAC analyses - permitting a single experimental manipulation. If this was not performed, validation of equivalent pull-down is needed.
3. In Figure 1I, it is unclear what exactly is represented by the scrambled tagged RNA. Scrambled CGG repeat sequence?
4. Some explanation is needed for the identification of SRSF6 as a strongly differentially interacting protein (Fig. 1E, F) but with lack of validation of the differential interaction in Fig. 1I.
5. Some explanation of what is meant in Figure 2B of SRSF2/8, SRSF4/6 and SRSF7/3. In addition, the duplicate analyses are unclear - are they simply duplicate crosses or is there some other meaning? Some of these are labeled (1) and (2), but others, e.g., SRSF2/8 are labeled (1) and (1). What do the numbers 1 and 2 signify? The is unclear from the figure legend, the text and the methods. The nomenclature is particularly important for the reader to understand since SRSF6 shows no differential interaction in Figure 1I.
6. The effect of siSRSF1 knockdown on survival, shown in Fig. 2E is very modest, albeit statistically significant. Some acknowledgement/discussion of this point should be made. The same is true of the survival data shown in Supplementary Figure 3A and D.
7. Line 222, the authors should insert "knockdown of" SF2/SRSF1 increased lifespan...
8. Extended data table (referred to on line 232) appears to be missing.
9. A demonstration of differential interaction of SRSF1, SRSF4/6, SRSF2 (Fig. 3A) with GGGGCC reporter is needed.
10. As with the CGG-90 flies shown in Figure 2, the effect of SRSF1 knockdown in Figure 3F on GGGGCC-28 flies is very modest, albeit statistically significant. How physiologically relevant are these effects?

11. Additional experimentation is needed to understand the mechanism underlying the effects of SRPK knockdown or inhibition. What effect do these manipulations have on SRSF1 interactions with the CGG and GGGGCC RNAs? Some understanding of how these manipulations are linked is required. Is phosphorylation of SRSF1 required for its nuclear translocation and binding to repeat-containing RNAs? If so, this should be demonstrated, along with the analysis of RNA binding.
12. The inclusion of SRSF6 in the discussion point (line 355) on interaction with CGG repeat RNA is flawed, as SRSF6 interacted non-specifically even with the scrambled reporter. Moreover, SRSF2 and SRSF3 were not validated for selective interaction with the CGG repeat RNA and should be.

Referee #3 (Remarks for Author):

The manuscript by Malik and colleagues demonstrates identification and functional characterization of several SR (serine/arginine-rich domain) proteins in Drosophila models of C9 and FXTAS. They observed that genetic or pharmacological modulation of SR protein kinases reduced RAN translation in cells and toxicities associated with C9 and FXTAS. Overall, this is an interesting study describing the role of SR proteins in human neurodegenerative diseases. There are multiple scientific flaws as listed below that the authors should address.

Major comments:
1. It is already known that SRSF1 is a genetic modifier of C9orf72 toxicity in fly models and it has been recently published. Hence, the current work is not novel. Here is the reference https://www.nature.com/articles/ncomms16063.
2. CGG RAN translation was enhanced by TG mediated stress response (Figure 1C), however, fewer CGG interactors were identified after ISR (Figure 1F). Is there a correlation between interactors of CGG and RAN translation?
3. What is the rationale behind choosing SRSF1, 2, 3 and 6 for the study and excluding SRSF 8 and 10? Also, why the authors choose not to focus on the top interactors of CGG repeat with or without stress? Similarly, Does SRSF3 does not interact with the CGG repeat (Figure 1i)?
4. What are the levels of SR proteins in response to CCG repeats expansion in the Drosophila model?
5. Many important controls are missing from their figures. Are the fly RNAi or the deficiency lines validated by qPCR or Western blot (Figure 2c)? Also, what does (1), (2) represent in the figure 2c. The terms haven't been explained in the text or legends. Are siSRSf1/dSF2 (1) and siSRSf1/dSF2 (2) different RNAi lines? If so, why do they show different degree of suppression? Are these siRNA or RNAi lines?
6. Survival assay in Figure 2E does not show drastic rescue of lifespan of CGG repeat expressing flies by SRSF1 knockdown as argued by the authors.
7. Does modulation of SR proteins affect the CGG or G4C2 repeat RNA or RAN translation?
8. The effect of SRPK1 on repeat expansion- mediated toxicity/RAN translation is either directly through modulating SR proteins or some other pathways? The authors did not provide sufficient evidence to support the correlation between SRPK1, SR proteins and CGG/G4C2 expansion mediated toxicity.
9. The paper started with finding the potential interactors of CGG repeat and studying the effect of some of the interactors such as SR protein on modulating the repeat expansion-mediated toxicity. However, the authors choose not to study the in-depth mechanism involving the SR protein and repeat expansion-toxicity and instead shifted the course of study towards SRPK1 without providing the sufficient correlation between SRPK1 and SR proteins and repeat expansion-toxicity.

Please find enclosed our revised manuscript entitled "**SRSF protein kinase 1 modulates RAN translation and suppresses CGG repeat toxicity".** We thank the editors and reviewers for their careful review of our manuscript and helpful suggestions to improve the work. All three reviewers noted that the manuscript had significant merit, was well written and contained a series of well designed experiments. However, some voiced concerns that the mechanism underlying how SPRK inhibition altered RAN translation and modulated toxicity was not well worked out. As such, we have focused significant efforts over the past 3 months at defining how these factors are acting. These new data provide a clear picture now of how SRPK inhibition both alters CGG repeat RNA localization and both directly and indirectly impairs RAN translation and correlate these effects with phenotypic rescue in two model systems across two repeat expansion disorders.

We now return to you what we feel is a significantly improved manuscript which we feel is ready for publication in EMBO Molecular Medicine.

Responses to specific reviewer comments:

Referee #1 (Remarks for Author):

The manuscript by Malik investigated the important role of RBPs in modulating RAN translation and repeat RNA pathology. In recent years, RAN translation of repeat RNA has been discovered in multiple neurodegenerative diseases, including FXTAS and ALS/FTD, which may play a critical role in disease pathogenesis. To elucidate the mechanisms underlying RAN translation in vivo, Malik and colleagues employed a new repeat RNA-tagging system to capture repeat associated RBPs under normal and stress conditions, by which they identified multiple SR proteins interacting with CGG repeat RNA. Using Drosophila models, they showed that inhibiting SR proteins and their modulator SRPK suppresses repeat-induced toxicity in eye morphology and survival. They further used cell models to indicate that pharmacological inhibition of SRPK1 suppresses repeat toxicity through RAN translation. Overall, the findings provide novel insight into RAN translation and repeat RNA pathology in FXTAS.

The experiments are well designed and the manuscript is well written. I have some comments and suggestions for the authors to consider.

We thank the reviewer for their supportive comments regarding our work.

Major comments
1. Context-specific interactions between repeat RNA and RBPs is quite interesting. Please provide an excel spreadsheet of the complete data? Supplemental figure 1 seems important (more than the GO analysis in Figure 1G), this might be better in the main figure.

We moved Supplemental Figure 1 into main figure 1 and moved our GO analysis into Extended View Figure 1. We also now include an excel spreadsheet with a complete presentation of our RNA/RBP interaction data as an appendix. Additionally, raw mass spectrometry data is now uploaded to PRIDE (Proteomics identification database) and we provide the accession number in the method section of the revised paper.

2. Drosophila genetic experiments are mostly based on RNAi knockdown. The authors should provide information on knockdown efficiency of each RNAi line (e.g., if this information exists from previous publications).

We now provide data demonstrating effective RNAi knockdown in Drosophila for both SRSF1 and SRPK1 in Extended View figure 3. Where available and viable, we have also included insertional mutagenesis lines or additional RNAi lines to confirm the findings shown.

3. In Drosophila genetic experiments, the authors mainly investigated eye morphology and survival, both of which are not true neurodegenerative phenotypes. It might be helpful to include Figure S3A in the main figure, which provides direct evidence indicating a neuronal interaction. Additionally, does the rough eye phenotype recapitulate in the ELAV-GS flies with SRSF1 and SRPK1 knock-down?

We have moved Figure S3A into main figure 3I.

Unfortunately, the ELAV-GS does not reliably recapitulate the rough eye phenotype in CGG repeat flies and ELAV expression of these repeats during embryonic development is lethal.

4. It wasn't discussed clearly how SR proteins modulate RAN translation of repeat RNA. Do SR proteins sequester repeat RNA in the nucleus? How do they recruit other components of the RAN translation machinery? Can the authors speculate the potential underlying mechanisms and include a few more sentences in the discussion section?

We agree with the reviewer that a more mechanistic understanding of how SR proteins and SRPK inhibition alters CGG repeat toxicity and RAN translation is needed. To this end, we have now conducted a series of additional experiments to address this question.

1) We confirmed a physical interaction and co-localization between SRSF1 and the repeat RNA by RNA IP, and by combinatorial Repeat RNA hybridization Chain Reaction (R-HCR) and immunohistochemistry to SRSF1 (Figure 2).
2) We demonstrate that SRPK inhibition leads to a change in SRSF phosphorylation and distribution within cells and that this correlates with nuclear retention of the CGG repeat RNA. Lastly we demonstrate that SRPK inhibitors can directly impair RAN translation *in vitro* in a rabbit reticulocyte lysate and in repeat RNA reporter transfected cells (which bypass any impact of SRSF on nucleocytoplasmic transport of the RNAs). (Figure 7 and Extended View Figure 4)

Taken together, these data suggest that SRPK inhibition indirectly precludes CGG repeat RNA transport from the nucleus to the cytoplasm by preventing SRSF phosphorylation. However, SRPK inhibition also directly and selectively suppresses CGG and G4C2 RAN translation. It is not yet clear whether this inhibition of RAN translation *in vitro* directly involves SRSF1.

5. Does altering SRSF phosphorylation change localization of repeat RNA constructs? Does stress change the subcellular localization of these RNA transcripts? Does this contribute to the

translational changes you see? RNA imaging experiments can be done with the PCP/PP7 constructs.

As noted above, SRPK inhibition leads to decreased phosphorylation of SRSF1, a redistribution of SRSF1 into the cytoplasm and a retention of CGG repeat RNA in the nucleus (Figure 7 and EV4). This partially explains the impaired RAN translation seen with SRPK inhibition and in Drosophila.

6. Does knockdown of SRPK1 change RAN reporter levels in flies or cells?

We now provide data showing a reduction in RAN aggregates in flies after knockdown of SRPK1 (Figure 4E).

7. Does altering SRSF phosphorylation with these drugs impact the ISR?

This is an interesting question that produced a telling result. SRPK inhibitors do not alter eIF2alpha phosphorylation in response to Thapsigargin (Tg) in untransfected cells. However, when cells are transfected with CGG repeats, those repeats trigger eIF2alpha phosphorylation as we previously described (Green et al, 2017, Nature Communications). In this context, addition of Tg normally further enhances this phosphorylation and boosts RAN translation. However, in the presence of SRPK inhibitors, this further boost in eIF2alpha phosphorylation triggered by thapsigargin is lost, as is the boost in RAN translation. We suggest that this loss of Tg effect in this context is through impaired RAN translation leading to less additional proteotoxic stress in the setting if ER stress activation by Tg.

Minor comments

1. Please add description of genetic background being appropriately controlled in this manuscript. This is critical especially when quantitative assays are performed. Please indicate what controls are used and whether different strains are backcrossed to equilibrate the genetic background.

All fly lines are maintained on a W118 background. While not all lines have been sufficiently backcrossed to assure no potential background effects, in general the effects on eye morphology are large and seen across multiple siRNA lines (which themselves are on a W118 background) and the Gene-switch studies showed survival effect only in presence of RU-486 drug (Conditional knockdown) suggesting observed phenotypes are specifically due to loss of target genes.

2. Although some terms are widely used in the field, it is helpful to list their full names, such as ISR.

We agree and we have now defined ISR as integrated stress response (Line 168-169). In general, unless we used a phrase more than 3 times, we have avoided use of abbreviations.

3. Please italicize Drosophila.

Agreed and done.

4. Figure 4A is a confusing schematic because these constructs aren't spliced, so why mention splicing?

We agree. A new schematic is now included in current figure 5A that does not have this issue and which better describes our model for how SRSF inhibition can impact RAN translation.

5. Explain more clearly why SRSF6 is enriched in mass spec but doesn't validate but immunoblot.

After further validation studies, we have removed SRSF6 from our discussed factors and removed the immunoblot data on this point- instead focusing our attention on SRSF1 and to a lesser degree SRSF2, which appear to mediate most of the observed effects.

Referee #2 (Remarks for Author):

The manuscript from Malik et al., entitled "SRSF protein kinase 1 modulates RAN translation and suppresses CGG repeat toxicity" describes a robust quantitative analysis of CGG repeat RNA protein interactors, the identification of SRSF proteins as contributors to toxicity using fly models of CGG- and G4C2- repeat toxicity, and modest therapeutic effects of knockdown and inhibition of the SRSF-regulating protein kinase SRPK. The results are interesting but require a bit more mechanistic connection in order to be convincing. This point, along with additional points are listed below for consideration.

We appreciate the reviewers response and (as discussed below) have now added significantly more mechanistic data to support our conclusions, including two full new figures (Figure 2 and Figure 7) plus additional new panels in Figures 4, 5, and 6.

1. The structure of the CGG repeat-containing RNA is likely key to the selectivity of the interacting proteins identified. Have the authors validated that the structure of the 90xCGG mRNA is the same when it is fused to GGG-nLuc and PP7 hairpins? At a minimum, it would seem that comparison of the PP7-fusion to unfused RANT reporter (and AUG reporter) would be need to establish lack of effect of the added sequence.

In Appendix 1/Supplemental Figure 1, we now include predicted RNA secondary structures for all RNA transcripts generated in m-Fold which demonstrate that the addition of PP7 sites are not predicted to directly alter repeat RNA structures. In addition, we compared RAN translation efficiency of PP7-tagged reporters used in this study with the base reporters (lacking PP7 loops) used to make the PP7 reporters, showing there is no significant difference (EV Figure 1B).

2. It is unclear if, prior to IP of SILAC-labeled protein lysates, the lysates of the different conditions were mixed in order to carry out a single pull-down. This is the central advantage of SILAC analyses - permitting a single experimental manipulation. If this was not performed, validation of equivalent pull-down is needed.

We used the same amount of total protein (quantified by BCA) for each condition to perform the FLAG pull-down and, finally, after wash FLAG beads were pooled together to perform a single mass spec experiment. Expression of PCP-FLAG and pull-down efficiency were measured by running small fractions of input and IP before performing mass spec (as shown bellow for one of the mass spec samples).

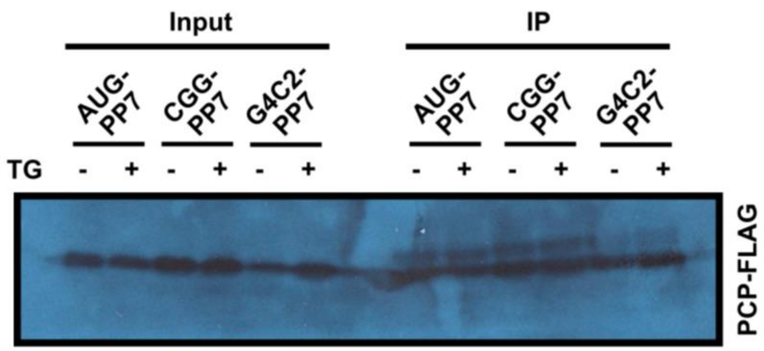

3. In Figure 1I, it is unclear what exactly is represented by the scrambled tagged RNA. Scrambled CGG repeat sequence?

It is not a scrambled CGG repeat, but a control sequence (for non-specific binding) containing part of the nLuc sequence. The control sequence is now included in the Appendix Table 1.

4. Some explanation is needed for the identification of SRSF6 as a strongly differentially interacting protein (Fig. 1E, F) but with lack of validation of the differential interaction in Fig. 1I.

After further validation studies, we have removed SRSF6 from our discussed factors and removed the immunoblot data on this point- instead focusing our attention on SRSF1 and to a lesser degree SRSF2, which appear to mediate most of the observed effects. As SRSF6 can interact with other SRSF proteins, we suspect that it came down in our initial SILAC studies as part of a larger complex.

5. Some explanation of what is meant in Figure 2B of SRSF2/8, SRSF4/6 and SRSF7/3. In addition, the duplicate analyses are unclear - are they simply duplicate crosses or is there some other meaning? Some of these are labeled (1) and (2), but others, e.g., SRSF2/8 are labeled (1) and (1). What do the numbers 1 and 2 signify? The is unclear from the figure legend, the text and the methods. The nomenclature is particularly important for the reader to understand since SRSF6 shows no differential interaction in Figure 1I.

We apologize for the confusion. For many SRSF proteins in *Drosophila,* there is a single orthologue for multiple SRSF factors in humans, which have significant functional overlap. This was the goal of the labeling, but we agree that it could be made clearer. To this end, we have now included the names of the most common human gene family member and then the the *Drosophila* name in the figure legend or results when appropriate. The full list of human genes and Drosophila orthologs are now presented in Appendix Table 2.

6. The effect of siSRSF1 knockdown on survival, shown in Fig. 2E is very modest, albeit statistically significant. Some acknowledgement/discussion of this point should be made. The same is true of the survival data shown in Supplementary Figure 3A and D.

We agree the survival effect is modest, especially compared to the eye phenotype rescue which is quite robust. We now directly acknowledge this in the results and discussion. It likely is less robust because SRSF proteins are important in many cellular processes.

Of note, we do not propose SRSF1 knockdown as a therapeutic option. Indeed, our pivot to and evaluation of SRPK1 inhibitors derives in part from the recognition that SRSF proteins are critical for many cellular processes. Thus, the hope is that modulating SRSF function though SRPK activity would be better tolerated (and more readily targeted pharmacologically) and thus carry a greater potential for further evaluation in mammalian model systems.

7. Line 222, the authors should insert "knockdown of" SF2/SRSF1 increased lifespan...

We have now inserted this phrase. Line 234: "…..knockdown of SRSF1 modulates GGGGCC repeat toxicity….."

8. Extended data table (referred to on line 232) appears to be missing.

We now include this table. In addition, all raw data is now uploaded in publically available/accessible databases.

9. A demonstration of differential interaction of SRSF1, SRSF4/6, SRSF2 (Fig. 3A) with GGGGCC reporter is needed.

This was previously established for GGGGCC repeats in Hautbergue et al, 2017 Nature Communications.

10. As with the CGG-90 flies shown in Figure 2, the effect of SRSF1 knockdown in Figure 3F on GGGGCC-28 flies is very modest, albeit statistically significant. How physiologically relevant are these effects?

Equating effect sizes in *Drosophila* to physiological relevance in humans is likely not appropriate. There is a biological interaction in a simple model system that we extend and observe in additional model systems with both pharmacologic and genetic data, providing robustness through multiple parallel analyses. In addition, while survival effect sizes with ubiquitous expression in adult flies is not robust, effects on the rough eye phenotypes observed are clear.

11. Additional experimentation is needed to understand the mechanism underlying the effects of SRPK knockdown or inhibition. What effect do these manipulations have on SRSF1 interactions with the CGG and GGGGCC RNAs? Some understanding of how these manipulations are linked is required. Is phosphorylation of SRSF1 required for its nuclear translocation and binding to

repeat-containing RNAs? If so, this should be demonstrated, along with the analysis of RNA binding.

We agree with the reviewer that a more mechanistic understanding of how SR proteins and SRPK inhibition alters CGG repeat toxicity and RAN translation is needed. To this end, we have now conducted a series of additional experiments to address this question.

1) We confirmed a physical interaction and co-localization between SRSF1 and the repeat RNA by RNA IP, and by combinatorial Repeat RNA hybridization Chain Reaction (R-HCR) and immunohistochemistry to SRSF1 (Figure 2).
2) We demonstrate that SRPK inhibition leads to a change in SRSF phosphorylation and distribution within cells and that this correlates with nuclear retention of the CGG repeat RNA. Lastly we demonstrate that SRPK inhibitors can directly impair RAN translation *in vitro* in a rabbit reticulocyte lysate and in repeat RNA reporter transfected cells (which bypass any impact of SRSF on nucleocytoplasmic transport of the RNAs). (Figure 7 and Extended View Figure 4)

Taken together, these data suggest that SRPK inhibition indirectly precludes CGG repeat RNA transport from the nucleus to the cytoplasm by preventing SRSF phosphorylation. However, SRPK inhibition also directly and selectively suppresses CGG and G4C2 RAN translation. It is not yet clear whether this inhibition of RAN translation *in vitro* directly involves SRSF1.

12. The inclusion of SRSF6 in the discussion point (line 355) on interaction with CGG repeat RNA is flawed, as SRSF6 interacted non-specifically even with the scrambled reporter. Moreover, SRSF2 and SRSF3 were not validated for selective interaction with the CGG repeat RNA and should be.

This has been removed and the focus of the discussion is maintained on SRSF1.

Referee #3 (Remarks for Author):

The manuscript by Malik and colleagues demonstrates identification and functional characterization of several SR (serine/arginine-rich domain) proteins in Drosophila models of C9 and FXTAS. They observed that genetic or pharmacological modulation of SR protein kinases reduced RAN translation in cells and toxicities associated with C9 and FXTAS. Overall, this is an interesting study describing the role of SR proteins in human neurodegenerative diseases. There are multiple scientific flaws as listed below that the authors should address.

We thank the reviewer and appreciate their recognition that this is an interesting study. We have endeavored to address all of the scientific concerns raised by this reviewer and discuss these below.

Major comments:
1. It is already known that SRSF1 is a genetic modifier of C9orf72 toxicity in fly models and it has been recently published. Hence, the current work is not novel. Here is the reference https://www.nature.com/articles/ncomms16063.

We are aware of this paper and cited it multiple times in our first submission (and do so again in this submission). The main focus of this manuscript is on CGG repeats, which are separate from GGGGCC repeats. SR proteins were not seen in prior in vitro pulldown studies of CGG repeats, suggesting that our methodology here may be capturing interactors that are important in the biology of these repeat RNAs that might be missed by other methods. In addition, it is interesting that GGGGCC and CGG repeats share RNA binding protein interactors in cells.

While we did perform some studies to validate the findings in the manuscript above with our own G4C2 model fly, the main focus here is on SRPK inhibition (not discussed or proposed in the paper above) and SRPK's role as a genetic modifier. As such, we feel there is significant novelty in this manuscript- and even more after the additional experiments we have added aimed at defining the mechanism(s) of action of SRPK1 inhibition on RAN translation.

2. CGG RAN translation was enhanced by TG mediated stress response (Figure 1C), however, fewer CGG interactors were identified after ISR (Figure 1F). Is there a correlation between interactors of CGG and RAN translation?

This is an interesting question. As we discuss more in the next question (Major point# 3), we mainly focused on the effect of loss of function of the target proteins in Drosophila for this study. Indeed, we observe many regulatory factors that specifically interact with CGG under stress and may affect RAN translation during stress such as – LARP1 (involved in TOP mRNA translation), UBA52 (context dependent ubiquitination of ribosome) and SYNCRIP/HNRNPQ (involved in FMRP IRES like translation). However, these SiRNAs directed to these factors showed either lethal or highly toxic interactions when we crossed with CGG fly. On the other hand, SR proteins suppressed CGG fly phenotype, thus we chose to focus on SR proteins for further studies.

3. What is the rationale behind choosing SRSF1, 2, 3 and 6 for the study and excluding SRSF 8 and 10? Also, why the authors choose not to focus on the top interactors of CGG repeat with or without stress? Similarly, Does SRSF3 does not interact with the CGG repeat (Figure 1i)?

As SRSF1 is the most prominent and best studied of the SR proteins, we chose to focus our attention on it and on its modulation via phosphorylation. Given that SRPK inhibitors are in clinical trials in humans, we felt this was the most translational finding from our initial screen.

In terms of why we focused on SR proteins rather than other hits, the rationale was pleiotropic. For some, the major effect of loss of expression in Drosophila was exacerbation of the underlying phenotype or their loss was associated with toxic phenotypes in isolation. In other cases, a clear set of orthologous genes with publically available lines for study were not readily available or the functions of the proteins were not well understood. In all cases, ongoing work is following up on other hits from these RNA pull-downs and these will be part of subsequent manuscripts utilizing this data.

4. What are the levels of SR proteins in response to CCG repeats expansion in the Drosophila model?

We measured the level of SRSF1 in +1CGG transfected cells. We didn't see any significant changes.

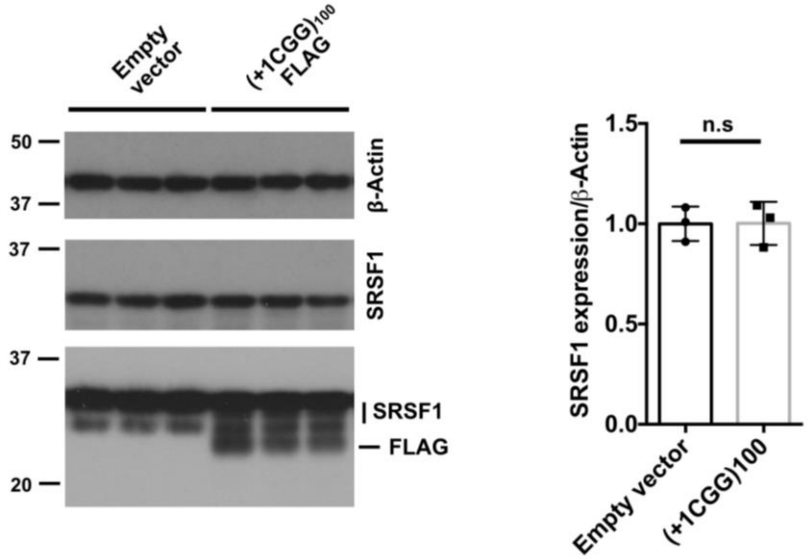

Figure: Quantification of SRSF1 level by immunoblot shows no significant changes in absence or presence of +1CGG reporter.

5. Many important controls are missing from their figures. Are the fly RNAi or the deficiency lines validated by qPCR or Western blot (Figure 2c)?

We validated that dSF2/SRSF1 and SRPK1 RNAi lines significantly lowered SRSF and SRPK1 RNA expression in flies, respectively and this data is included in Extended View Figure 3. For other lines, we have generally tried to include 2 RNAi or deficiency lines for each when feasible and include citations where there is data already available demonstrating their efficacy at lowering expression of the targeted genes.

Also, what does (1), (2) represent in the figure 2c. The terms haven't been explained in the text or legends.

We apologize for the confusion. These are separate siRNA lines- now more clearly specified in the figure (now Figure 3) and figure legend as well as spelled out in detail in the methods.

Are siSRSf1/dSF2 (1) and siSRSf1/dSF2 (2) different RNAi lines? If so, why do they show different degree of suppression? Are these siRNA or RNAi lines?

Yes, these are two different UAS-RNAi lines that activate RNAi expression targeted to dSF2 in response to UAS binding by GAL4.

6. Survival assay in Figure 2E does not show drastic rescue of lifespan of CGG repeat expressing flies by SRSF1 knockdown as argued by the authors.

We have removed any words claiming drastic rescue and agree that any such claim was inaccurate. The results are modest, but statistically significant, which we now state. Equating effect sizes in *Drosophila* to physiological relevance in humans is likely not appropriate. We do feel that the data supports a biological interaction in a simple model system that we extend and observe in additional model systems with both pharmacologic and genetic data. In addition, while survival effect sizes with ubiquitous expression in adult flies is not robust, effects on the rough eye phenotypes observed are clear (indeed, one of the strongest suppressors we have ever observed).

7. Does modulation of SR proteins affect the CGG or G4C2 repeat RNA or RAN translation?

Please see our response to concern #8 below. In short- it impacts both CGG repeat RNA behavior and CGG or G4C2 repeat associated RAN translation (directly through translational inhibition and indirectly through impaired nucleocytoplasmic RNA transport).

8. The effect of SRPK1 on repeat expansion- mediated toxicity/RAN translation is either directly through modulating SR proteins or some other pathways? The authors did not provide sufficient evidence to support the correlation between SRPK1, SR proteins and CGG/G4C2 expansion mediated toxicity.

We agree with the reviewer that a more mechanistic understanding of how SR proteins and SRPK inhibition alters CGG repeat toxicity and RAN translation is needed. To this end, we have now conducted a series of additional experiments to address this question.

1) We confirmed a physical interaction and co-localization between SRSF1 and the repeat RNA by RNA IP, and by combinatorial Repeat RNA hybridization Chain Reaction (R-HCR) and immunohistochemistry to SRSF1 (Figure 2).
2) We demonstrate that SRPK inhibition leads to a change in SRSF phosphorylation and distribution within cells and that this correlates with nuclear retention of the CGG repeat RNA. Lastly we demonstrate that SRPK inhibitors can directly impair RAN translation *in vitro* in a rabbit reticulocyte lysate and in repeat RNA reporter transfected cells (which bypass any impact of SRSF on nucleocytoplasmic transport of the RNAs). (Figure 7 and Extended View Figure 4)

Taken together, these data suggest that SRPK inhibition indirectly precludes CGG repeat RNA transport from the nucleus to the cytoplasm by preventing SRSF phosphorylation. However, SRPK inhibition also directly and selectively suppresses CGG and G4C2 RAN translation. It is not yet clear whether this inhibition of RAN translation *in vitro* directly involves SRSF1.

9. The paper started with finding the potential interactors of CGG repeat and studying the effect of some of the interactors such as SR protein on modulating the repeat expansion-mediated toxicity. However, the authors choose not to study the in-depth mechanism involving the SR protein and repeat expansion-toxicity and instead shifted the course of study towards SRPK1 without providing the sufficient correlation between SRPK1 and SR proteins and repeat expansion-toxicity.

While we agree that our story did not take the most linear of routes, that is the way science works sometimes!
In all seriousness, the reasons we pivoted from SRSF proteins to evaluation of SRPK1 inhibitors derives from the recognition that SRSF proteins are critical for many cellular processes and from the recognition that another group already studied SRSF proteins in the context of G4C2 repeats- as pointed out by this reviewer. This pivot revealed a novel target that has multiple compounds already in development in humans- compounds that we tested here in both human cells and rodent neurons. I personally feel that the first author should be commended for his creativity and insight.

To make this connection more robust and to specifically address the reviewer concerns, we now demonstrate that these inhibitors directly impact SRSF1 phosphorylation and distribution in cells (Figure 7 and EV4), that this altered distribution correlates with retention of the CGG repeat RNA in the nucleus (Figure 7), and that this retention is part of (but not the only) reason why SRPK inhibitors suppress RAN translation suppress CGG repeat toxicity in neurons.

20th Aug 2021

Dear Dr. Todd,

Thank you for the submission of your revised manuscript to EMBO Molecular Medicine. I am pleased to inform you that we will be able to accept your manuscript pending the following final amendments:

1) Please implement all suggestions made by referee 2.
2) Figures: Please upload individual, high-resolution file for each main and EV figure. Place EV figure legends in the main manuscript file after main figure legends. For more information on figure presentation please check "Author Guidelines".
https://www.embopress.org/page/journal/17574684/authorguide#datapresentationformat
3) In the main manuscript file, please do the following:
- Correct/answer the track changes suggested by our data editors by working from the attached/uploaded document.
- Limit keywords to max. 5.
- Make sure that all special characters display well.
- Remove the impact statement.
- Add callouts for Figure 4E and F.
- Add author contributions. The nature of every author's contribution must be specified both in the manuscript submission system (using the CRediT contributor role taxonomy) and in the manuscript under the heading "Author Contributions". Please check "Author Guidelines" for more information.
https://www.embopress.org/page/journal/17574684/authorguide#authorshipguidelines
- Add "Conflict of Interest" statement.
- In M&M, statistical paragraph should reflect all information that you have filled in the Authors Checklist, especially regarding randomization, blinding, replication etc.
- In addition to the accession number please provide URL for all deposited datasets. Please be aware that all datasets should be made freely available upon acceptance, without restriction. Use the following format to report the accession number of your data:

The datasets produced in this study are available in the following databases:
[data type]: [full name of the resource] [accession number/identifier] ([doi or URL or identifiers.org/DATABASE:ACCESSION])

Please check "Author Guidelines" for more information.
https://www.embopress.org/page/journal/17574684/authorguide#availabilityofpublishedmaterial
4) Appendix: Please and table of content and rename Figure S1 to "Appendix Figure S1".
5) Tables: Please move EV tables to Appendix and rename them "Appendix Table S1" etc. also in the main text.
6) Funding: Please fuse this section with "Acknowledgement" and make sure that information about all sources of funding are complete in both our submission system and in the manuscript.
7) The Paper Explained: Please provide "The Paper Explained" and add it to the main manuscript text. Please check "Author Guidelines" for more information.
https://www.embopress.org/page/journal/17574684/authorguide#researcharticleguide
8) Synopsis: Every published paper now includes a 'Synopsis' to further enhance discoverability. Synopses are displayed on the journal webpage and are freely accessible to all readers. They include separate synopsis image and synopsis text.

- Synopsis image: Please provide a striking image or visual abstract as a high-resolution jpeg file 550 px-wide x (250-400)-px high to illustrate your article.
- Synopsis text: Please provide a short standfirst (maximum of 300 characters, including space) as well as 2-5 one sentence bullet points that summarise the paper as a .doc file. Please write the bullet points to summarise the key NEW findings. They should be designed to be complementary to the abstract - i.e. not repeat the same text. We encourage inclusion of key acronyms and quantitative information (maximum of 30 words / bullet point). Please use the passive voice.
- Please check your synopsis text and image before submission with your revised manuscript. Please be aware that in the proof stage minor corrections only are allowed (e.g., typos).
9) For more information: There is space at the end of each article to list relevant web links for further consultation by our readers. Could you identify some relevant ones and provide such information as well? Some examples are patient associations, relevant databases, OMIM/proteins/genes links, author's websites, etc...
10) As part of the EMBO Publications transparent editorial process initiative (see our Editorial at http://embomolmed.embopress.org/content/2/9/329), EMBO Molecular Medicine will publish online a Review Process File (RPF) to accompany accepted manuscripts. This file will be published in conjunction with your paper and will include the anonymous referee reports, your point-by-point response and all pertinent correspondence relating to the manuscript. Let us know whether you agree with the publication of the RPF and as here, if you want to remove or not any figures from it prior to publication. Please note that the Authors checklist will be published at the end of the RPF.
11) Please provide a point-by-point letter INCLUDING my comments as well as the reviewer's reports and your detailed responses (as Word file).

I look forward to reading a new revised version of your manuscript as soon as possible.

Yours sincerely,

Zeljko Durdevic

Zeljko Durdevic
Editor
EMBO Molecular Medicine

***** Reviewer's comments *****

Referee #1 (Comments on Novelty/Model System for Author):

This is a revised manuscript with several additional experiments that strengthen the authors' conclusions and increase the novelty and impact of the paper

Referee #1 (Remarks for Author):

The authors have made impressive efforts to thoughtfully address all of the reviewers' concerns and the revised manuscript, in my opinion, is substantially improved and this work will make an important contribution to the field's understanding of molecular mechanisms of RAN translation regulation. I recommend that this manuscript is now suitable for publication.

Referee #2 (Comments on Novelty/Model System for Author):

The manuscript has been much improved with details that support the above selections.

Referee #2 (Remarks for Author):

The authors have largely addressed this reviewer's concerns. However, there remains language in the description of the effect of modifying expression levels of SRSF1 and SRPK1 in Drosophila models that overstates the results. "Significantly enhanced lifespan" implies a substantial effect. "A modest but statistically significant effect on lifespan" would be a more accurate description of several of these results in Figures 3 and EV3.

Minor comment: The description of the reduced GFP inclusions produced by RAN translation in the fly eye refers to Figure 3E,F. This should be corrected to Figure 4E,F.

Figure EV3. The Y-axis title in part D needs correction.

Referee #3 (Remarks for Author):

None

2nd Revision - Editorial Decision 30th Aug 2021

We are pleased to inform you that your manuscript is accepted for publication and is now being sent to our publisher to be included in the next available issue of EMBO Molecular Medicine.

EMBO PRESS

YOU MUST COMPLETE ALL CELLS WITH A PINK BACKGROUND

PLEASE NOTE THAT THIS CHECKLIST WILL BE PUBLISHED ALONGSIDE YOUR PAPER

Corresponding Author Name: Peter K Todd
Journal Submitted to: EMBO Molecular Medicine
Manuscript Number: EMM-2021-14163

Reporting Checklist For Life Sciences Articles (Rev. June 2017)

This checklist is used to ensure good reporting standards and to improve the reproducibility of published results. These guidelines are consistent with the Principles and Guidelines for Reporting Preclinical Research issued by the NIH in 2014. Please follow the journal's authorship guidelines in preparing your manuscript.

A- Figures
1. Data
The data shown in figures should satisfy the following conditions:

- the data were obtained and processed according to the field's best practice and are presented to reflect the results of the experiments in an accurate and unbiased manner.
- figure panels include only data points, measurements or observations that can be compared to each other in a scientifically meaningful way.
- graphs include clearly labeled error bars for independent experiments and sample sizes. Unless justified, error bars should not be shown for technical replicates.
- if n< 5, the individual data points from each experiment should be plotted and any statistical test employed should be justified
- Source Data should be included to report the data underlying graphs. Please follow the guidelines set out in the author ship guidelines on Data Presentation.

2. Captions
Each figure caption should contain the following information, for each panel where they are relevant:

- a specification of the experimental system investigated (eg cell line, species name).
- the assay(s) and method(s) used to carry out the reported observations and measurements
- an explicit mention of the biological and chemical entity(ies) that are being measured.
- an explicit mention of the biological and chemical entity(ies) that are altered/varied/perturbed in a controlled manner.

- the exact sample size (n) for each experimental group/condition, given as a number, not a range;
- a description of the sample collection allowing the reader to understand whether the samples represent technical or biological replicates (including how many animals, litters, cultures, etc.).
- a statement of how many times the experiment shown was independently replicated in the laboratory.
- definitions of statistical methods and measures:
  - common tests, such as t-test (please specify whether paired vs. unpaired), simple χ2 tests, Wilcoxon and Mann-Whitney tests, can be unambiguously identified by name only, but more complex techniques should be described in the methods section;
  - are tests one-sided or two-sided?
  - are there adjustments for multiple comparisons?
  - exact statistical test results, e.g., P values = x but not P values < x;
  - definition of 'center values' as median or average;
  - definition of error bars as s.d. or s.e.m.

Any descriptions too long for the figure legend should be included in the methods section and/or with the source data.

In the pink boxes below, please ensure that the answers to the following questions are reported in the manuscript itself. Every question should be answered. If the question is not relevant to your research, please write NA (non applicable). We encourage you to include a specific subsection in the methods section for statistics, reagents, animal models and human subjects.

USEFUL LINKS FOR COMPLETING THIS FORM

http://www.antibodypedia.com
http://1degreebio.org
http://www.equator-network.org/reporting-guidelines/improving-bioscience-research-repor
http://grants.nih.gov/grants/olaw/olaw.htm
http://www.mrc.ac.uk/Ourresearch/Ethicsresearchguidance/Useofanimals/index.htm
http://ClinicalTrials.gov
http://www.consort-statement.org
http://www.consort-statement.org/checklists/view/32-consort/66-title
http://www.equator-network.org/reporting-guidelines/reporting-recommendations-for-tumo
http://datadryad.org
http://figshare.com
http://www.ncbi.nlm.nih.gov/gap
http://www.ebi.ac.uk/ega
http://biomodels.net/
http://biomodels.net/miriam/
http://jjj.biochem.sun.ac.za
https://osp.od.nih.gov/biosafety-biosecurity-and-emerging-biotechnology/
http://www.selectagents.gov/

B- Statistics and general methods

Please fill out these boxes (Do not worry if you cannot see all your text once you press return)

1.a. How was the sample size chosen to ensure adequate power to detect a pre-specified effect size?	We used a mimimum of three independent biological samples (n >3) with technical replication of results from each sample. We usually had >6 biological replicates. We did not prespecify the number of technical or biological replicates.
1.b. For animal studies, include a statement about sample size estimate even if no statistical methods were used.	For Fly eye screening sample sizes were based on earlier published standards.
2. Describe inclusion/exclusion criteria if samples or animals were excluded from the analysis. Were the criteria pre-established?	N/A
3. Were any steps taken to minimize the effects of subjective bias when allocating animals/samples to treatment (e.g. randomization procedure)? If yes, please describe.	In order to minimize the effects of subjective bias, fly screening was done by at least two independent investigators with some of the raters blinded to genotype.
For animal studies, include a statement about randomization even if no randomization was used.	Fly pheotypes were generated from multiple crosses. No formal randomization was used.
4.a. Were any steps taken to minimize the effects of subjective bias during group allocation or/and when assessing results (e.g. blinding of the investigator)? If yes please describe.	In order to minimize the effects of subjective bias, major fly experiments were done by multiple people independently.
4.b. For animal studies, include a statement about blinding even if no blinding was done	Eye necrosis/width meaurements were done by blind data analysis
5. For every figure, are statistical tests justified as appropriate?	Data are presented as mean ± standard deviation (SD) of minimally three independent samples. P-values were calculated with Graphpad Prism with appropriate statistics
Do the data meet the assumptions of the tests (e.g., normal distribution)? Describe any methods used to assess it.	For studies with sufficent N's to confirm normality, we used the confirmed that the data was technically normal using a D'Agostino-Pearson Normality test
Is there an estimate of variation within each group of data?	We did not assume equal variance between groups

Is the variance similar between the groups that are being statistically compared?	Variance was generally comparable but we did not assume equal variance between groups when we analyzed them statistically.

C- Reagents

6. To show that antibodies were profiled for use in the system under study (assay and species), provide a citation, catalog number and/or clone number, supplementary information or reference to an antibody validation profile. e.g., Antibodypedia (see link list at top right), 1DegreeBio (see link list at top right).	FLAG-M2 at 1:1000 dilution (mouse, Sigma F1804), 1:2500 β-Actin (mouse, Sigma A1978), 1:1000 SRSF1 (Rabbit, Proteintech 12929-2-AP), 1:1000 SRSF2 (Rabbit, Proteintech 20371-1-AP), 1:1000 Anti-Phosphoepitope SR proteins, clone 1H4 mouse (MABE50, Millipore Sigma), GAPDH (mouse, Santa Cruz sc-32233), 1:1000 eIF2α/EIF2S1 (phospho S51) (rabbit, Abcam ab32157) and 1:1000 GFP (mouse, Roche/Sigma 11814460001). HRP-conjugated goat-anti-mouse (115-035-146) or goat-anti-rabbit (111-035-144) secondary antibodies (Jackson ImmunoResearch Laboratories)
7. Identify the source of cell lines and report if they were recently authenticated (e.g., by STR profiling) and tested for mycoplasma contamination.	HEK293T and U2OS cell lines were purchased from ATCC and regularly chacked for mycoplasma contamination

* for all hyperlinks, please see the table at the top right of the document

D- Animal Models

8. Report species, strain, gender, age of animals and genetic modification status where applicable. Please detail housing and husbandry conditions and the source of animals.	Drosophila resources are presented in Appendix Table 2
9. For experiments involving live vertebrates, include a statement of compliance with ethical regulations and identify the committee(s) approving the experiments.	All Animal studies were approved by and complied with the ethical regulations of UCUCA.
10. We recommend consulting the ARRIVE guidelines (see link list at top right) (PLoS Biol. 8(6), e1000412, 2010) to ensure that other relevant aspects of animal studies are adequately reported. See author guidelines, under 'Reporting Guidelines'. See also: NIH (see link list at top right) and MRC (see link list at top right) recommendations. Please confirm compliance.	N/A

E- Human Subjects

11. Identify the committee(s) approving the study protocol.	N/A
12. Include a statement confirming that informed consent was obtained from all subjects and that the experiments conformed to the principles set out in the WMA Declaration of Helsinki and the Department of Health and Human Services Belmont Report.	N/A
13. For publication of patient photos, include a statement confirming that consent to publish was obtained.	N/A
14. Report any restrictions on the availability (and/or on the use) of human data or samples.	N/A
15. Report the clinical trial registration number (at ClinicalTrials.gov or equivalent), where applicable.	N/A
16. For phase II and III randomized controlled trials, please refer to the CONSORT flow diagram (see link list at top right) and submit the CONSORT checklist (see link list at top right) with your submission. See author guidelines, under 'Reporting Guidelines'. Please confirm you have submitted this list.	N/A
17. For tumor marker prognostic studies, we recommend that you follow the REMARK reporting guidelines (see link list at top right). See author guidelines, under 'Reporting Guidelines'. Please confirm you have followed these guidelines.	N/A

F- Data Accessibility

18: Provide a "Data Availability" section at the end of the Materials & Methods, listing the accession codes for data generated in this study and deposited in a public database (e.g. RNA-Seq data: Gene Expression Omnibus GSE39462, Proteomics data: PRIDE PXD000208 etc.) Please refer to our author guidelines for 'Data Deposition'.<br><br>Data deposition in a public repository is mandatory for:<br>a. Protein, DNA and RNA sequences<br>b. Macromolecular structures<br>c. Crystallographic data for small molecules<br>d. Functional genomics data<br>e. Proteomics and molecular interactions	Mass spectrometry data is deposited to the ProteomeXchange Consortium via the PRIDE partner repository with the dataset identifier PXD027000.
19. Deposition is strongly recommended for any datasets that are central and integral to the study; please consider the journal's data policy. If no structured public repository exists for a given data type, we encourage the provision of datasets in the manuscript as a Supplementary Document (see author guidelines under 'Expanded View' or in unstructured repositories such as Dryad (see link list at top right) or Figshare (see link list at top right).	Raw mass spectrometry data is provided in Data Set EV1. Raw data for each figure is presented in Data Set EV2.
20. Access to human clinical and genomic datasets should be provided with as few restrictions as possible while respecting ethical obligations to the patients and relevant medical and legal issues. If practically possible and compatible with the individual consent agreement used in the study, such data should be deposited in one of the major public access-controlled repositories such as dbGAP (see link list at top right) or EGA (see link list at top right).	N/A
21. Computational models that are central and integral to a study should be shared without restrictions and provided in a machine-readable form. The relevant accession numbers or links should be provided. When possible, standardized format (SBML, CellML) should be used instead of scripts (e.g. MATLAB). Authors are strongly encouraged to follow the MIRIAM guidelines (see link list at top right) and deposit their model in a public database such as Biomodels (see link list at top right) or JWS Online (see link list at top right). If computer source code is provided with the paper, it should be deposited in a public repository or included in supplementary information.	N/A

G- Dual use research of concern

22. Could your study fall under dual use research restrictions? Please check biosecurity documents (see link list at top right) and list of select agents and toxins (APHIS/CDC) (see link list at top right). According to our biosecurity guidelines, provide a statement only if it could.	No
